# Supplementary material for: Phosphatase specificity principles uncovered by MRBLE:Dephos and global substrate identification
Source: Mol Syst Biol. 2023 Nov 2;19(12):e11782. doi: 10.15252/msb.202311782 (PMC10698503; doi:10.15252/msb.202311782)
Supplement: Supplementary file 1 — Appendix S1 [file MSB-19-e11782-s005.pdf]

Appendix for Hein et al.

Content: Appendix Figure S1-12.

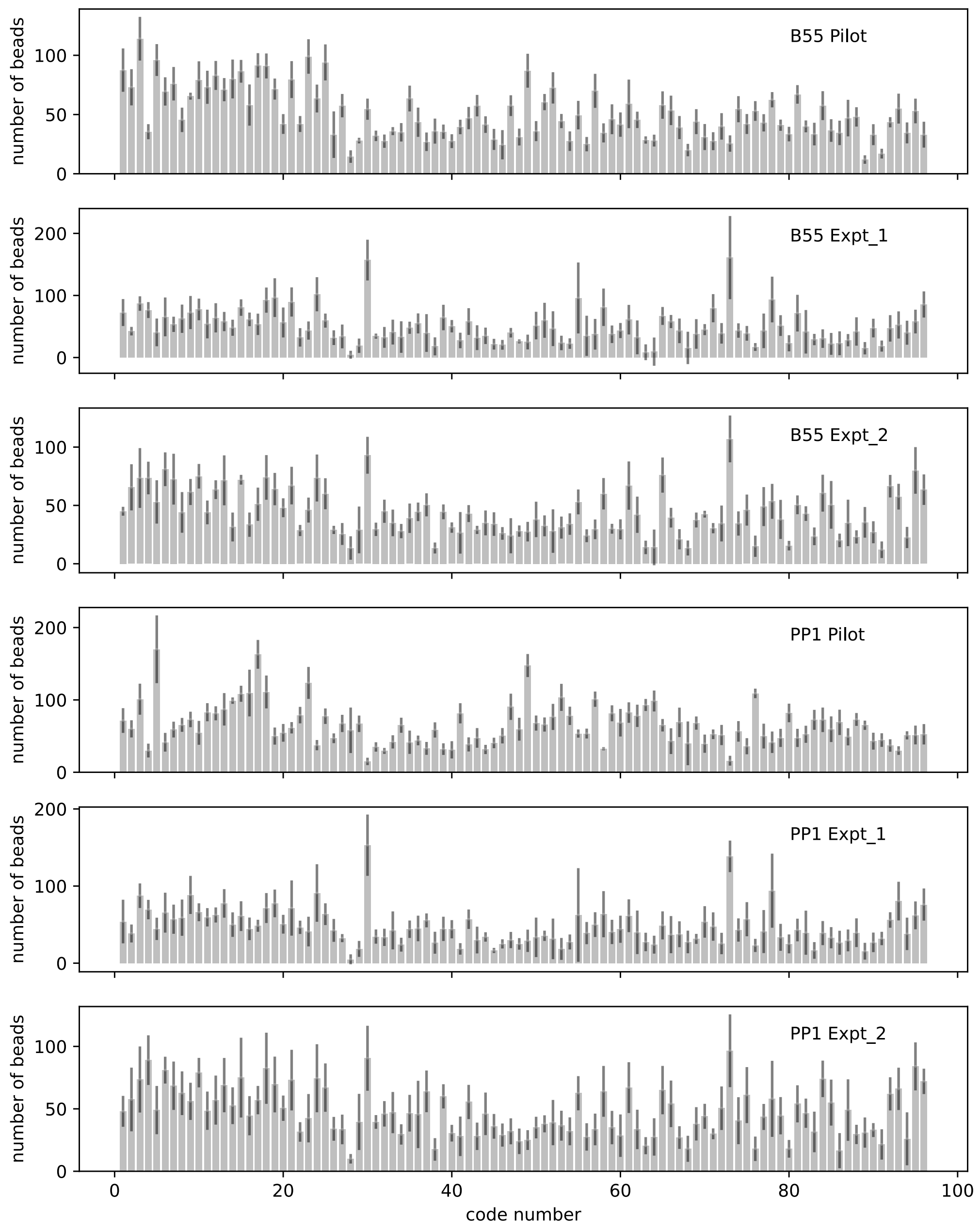

Appendix Figure S1. Number of MRBLEs beads profiled per code per experiment. Each bar represents the median number of beads for a given code profiled over all timepoints within an experiment; the error bar denotes the standard deviation.

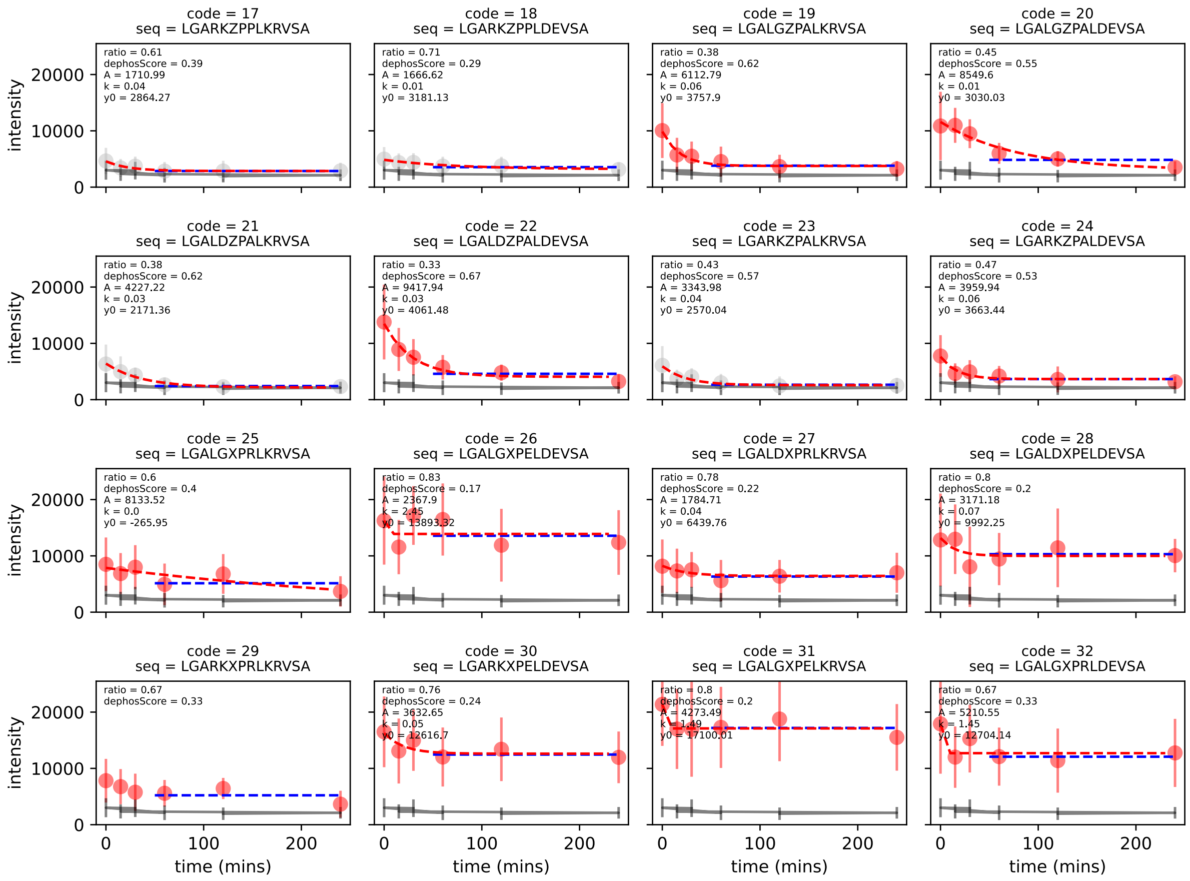

Appendix Figure S2. Example raw Cy5 intensity traces over time for a subset of codes within PP1 experiment #1. Red markers denote the median intensity for all beads at a given timepoint; error bars denote the standard deviation; the red dashed line indicates the best fit to a single exponential; the blue line denotes the median signal across the final 3 timepoints, and the black line and associated error bars denotes the signal for negative control beads presenting an unphosphorylated peptide. Codes for which the initial signal is not substantially greater than the signal for the negative control peptide were filtered out from downstream analysis (indicated by light gray markers, see Materials and Methods for details).

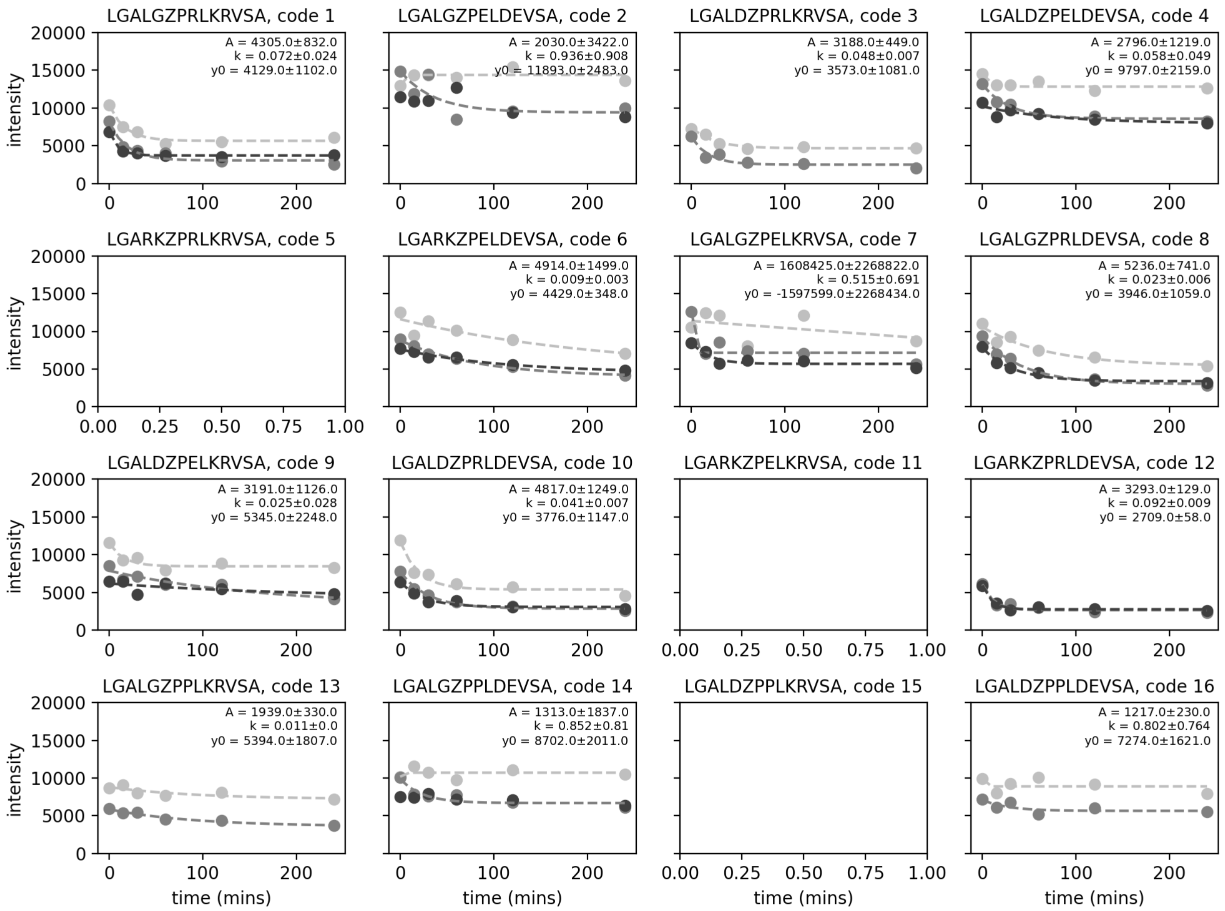

Appendix Figure S3. Measured DyLight 650 intensities over time for MRBLE-bound peptides after incubation with PP1. Each marker shade denotes a different experimental replicate; dotted lines indicate a single exponential fit within each experimental replicate. Annotations report mean exponential fit parameters ± standard deviation across at least 2 independent replicates. To filter out any codes for which phosphopeptide synthesis was unsuccessful, we report data for a given code and experiment only if the median bead intensity for that code at the first timepoint exceeds the median + 2 standard deviations of the bead intensity for a negative control code.

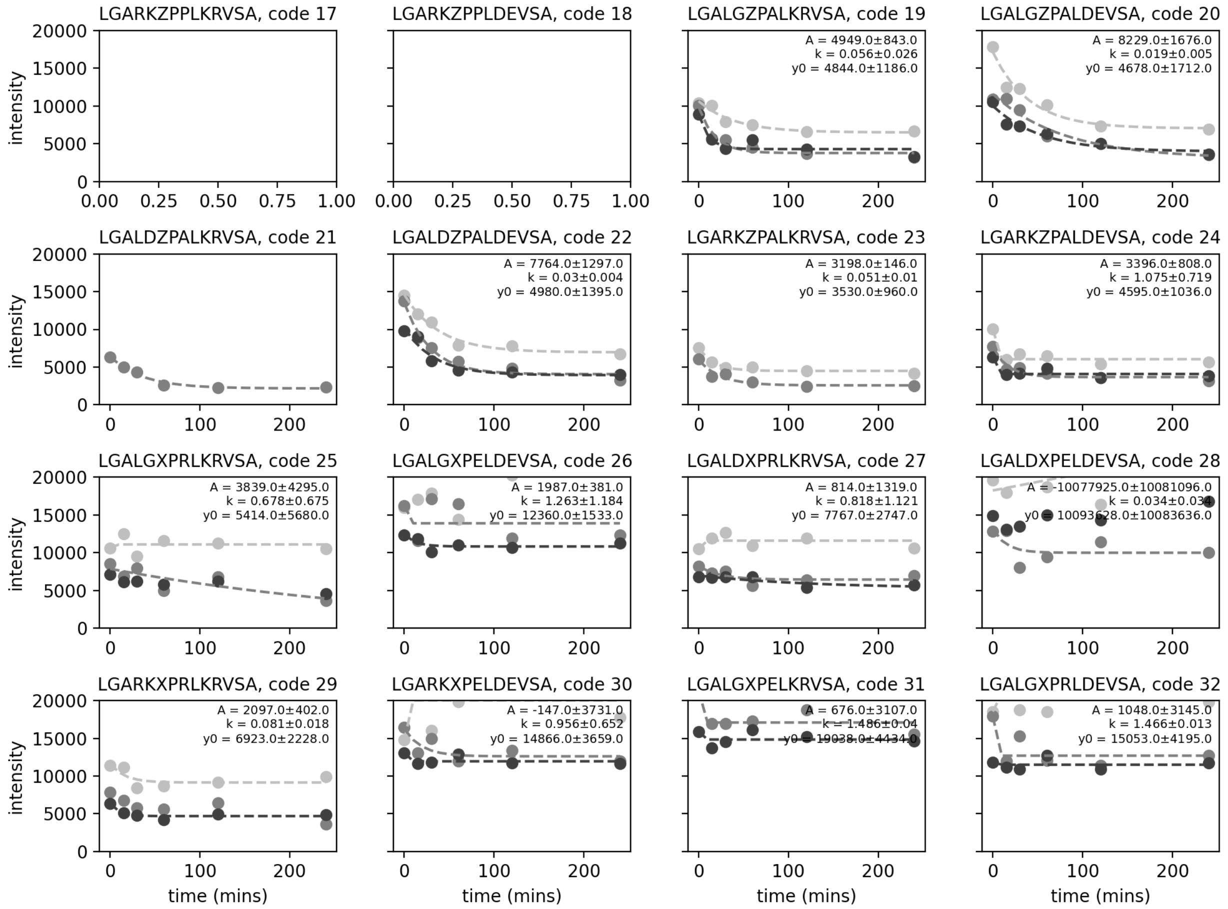

Appendix Figure S3 (continued).

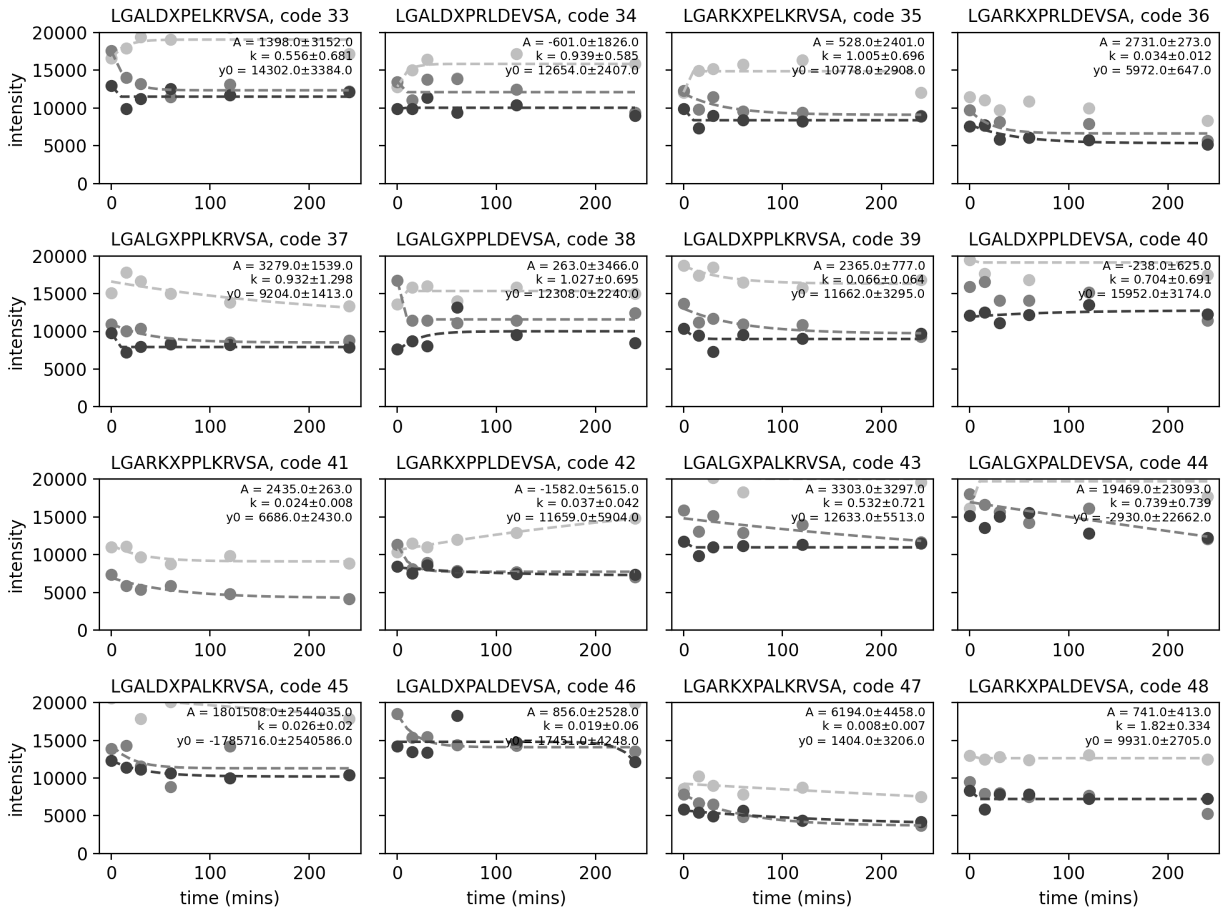

Appendix Figure S3 (continued).

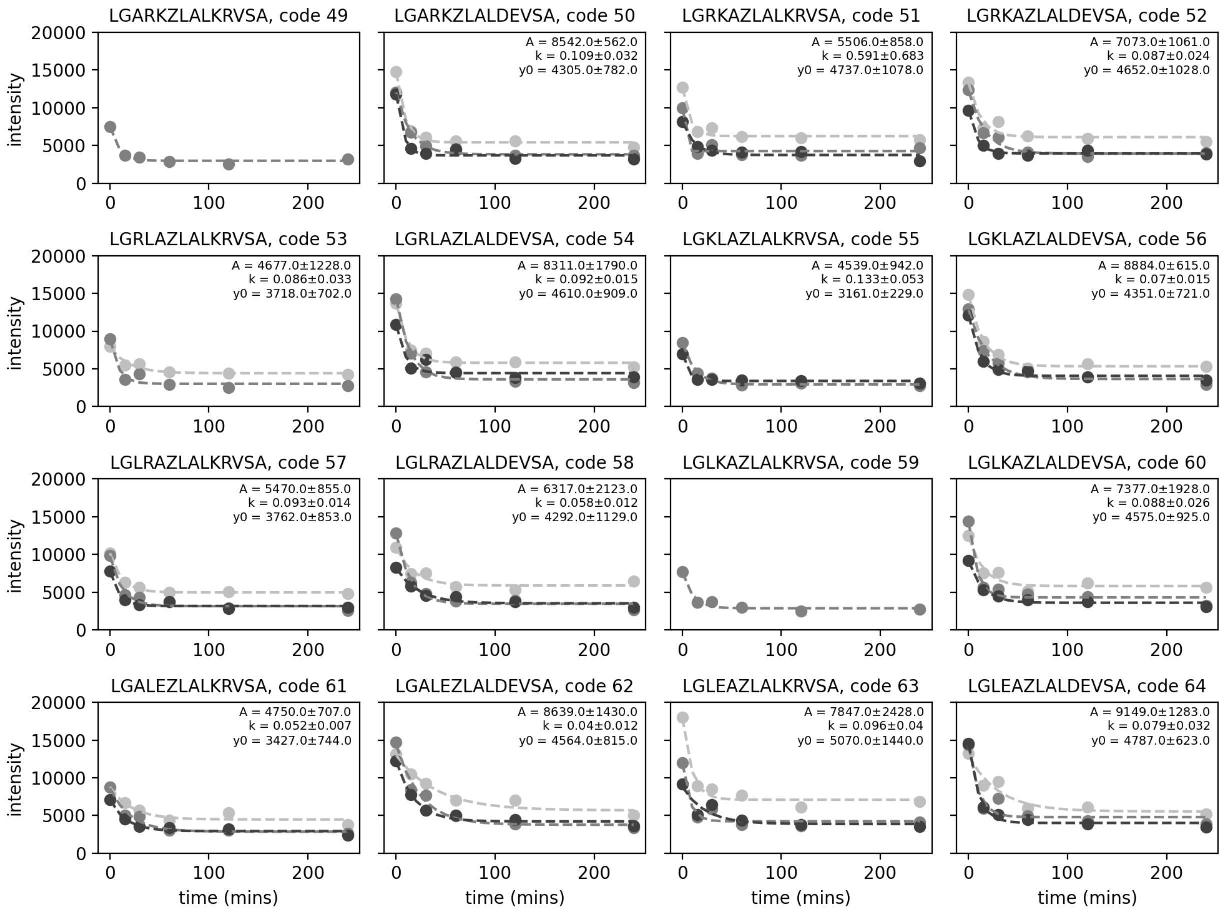

Appendix Figure S3 (continued).

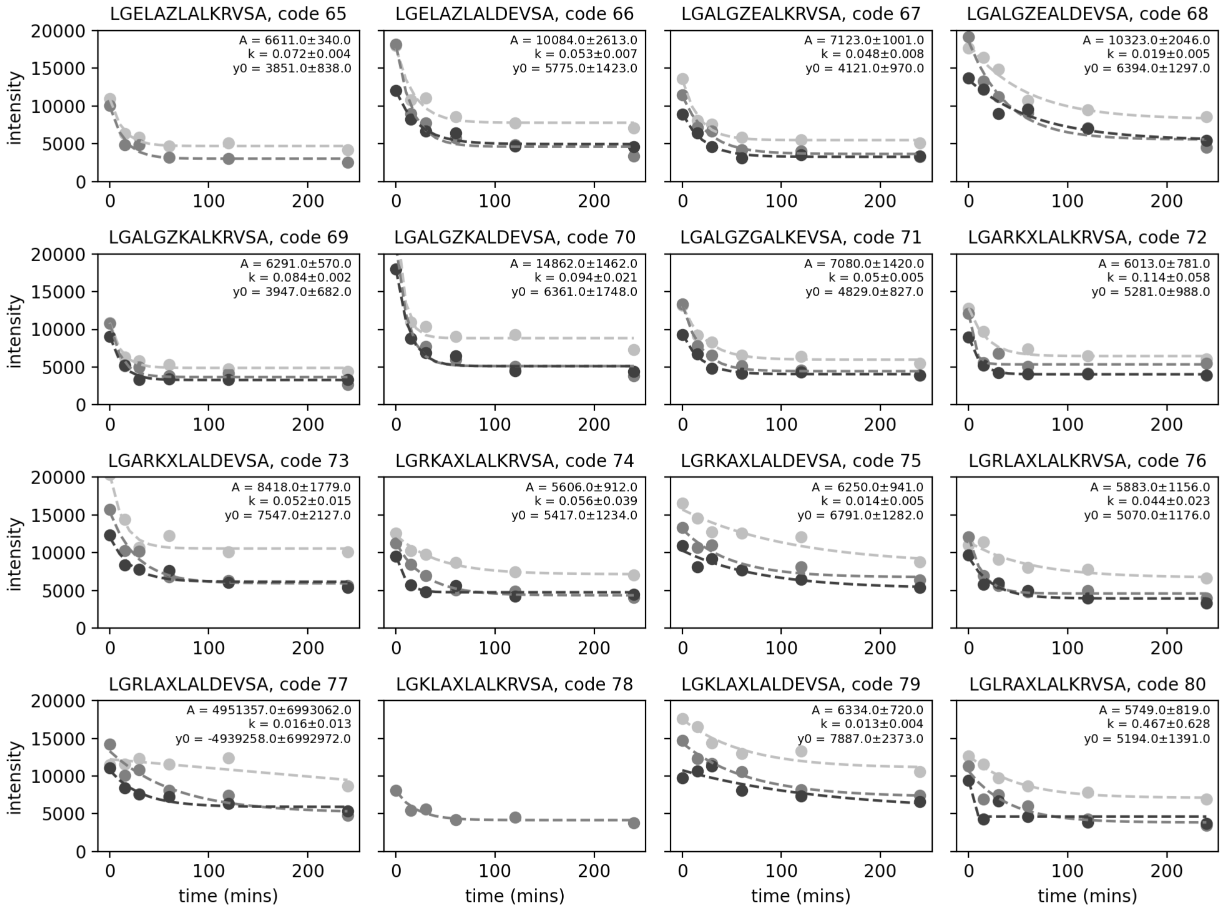

Appendix Figure S3 (continued).

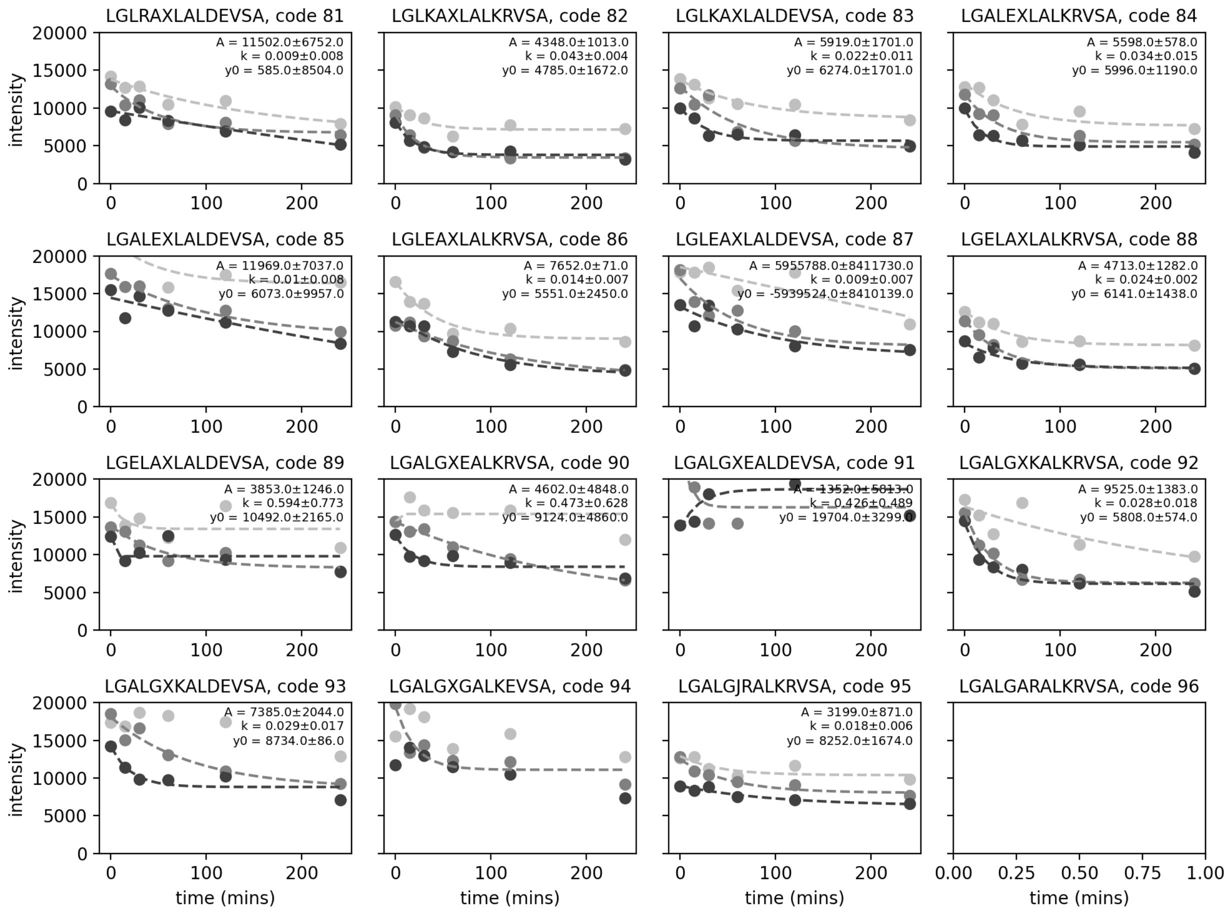

Appendix Figure S3 (continued).

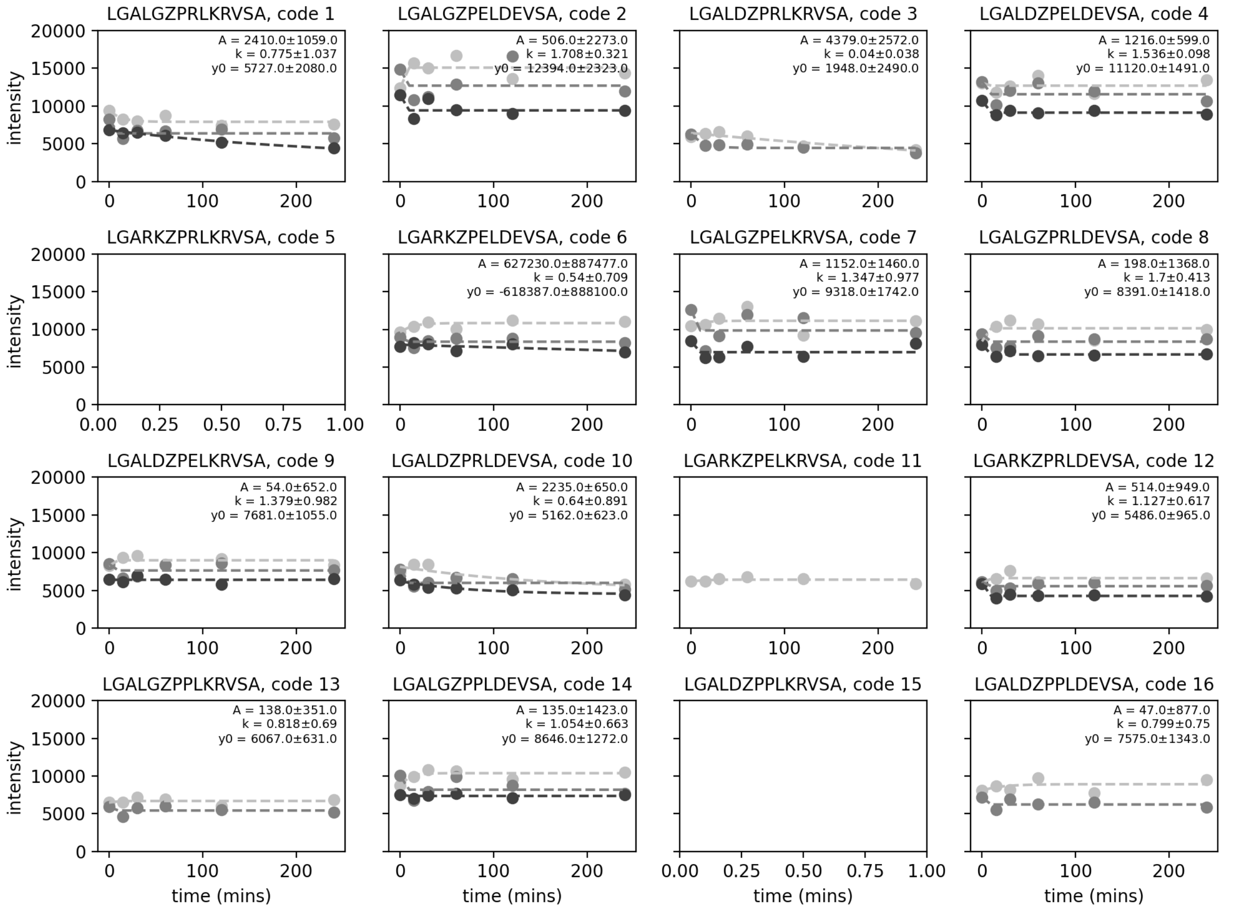

Appendix Figure S4. Measured DyLight 650 intensities over time for MRBLE-bound peptides after incubation with PP2A-B55. Each marker shade denotes a different experimental replicate; dotted lines indicate a single exponential fit within each experimental replicate. Annotations report mean exponential fit parameters across 3 independent replicates. To filter out any codes for which phosphopeptide synthesis was unsuccessful, we report data for a given code and experiment only if the median bead intensity for that code at the first timepoint exceeds the median + 2 standard deviations of the bead intensity for a negative control code.

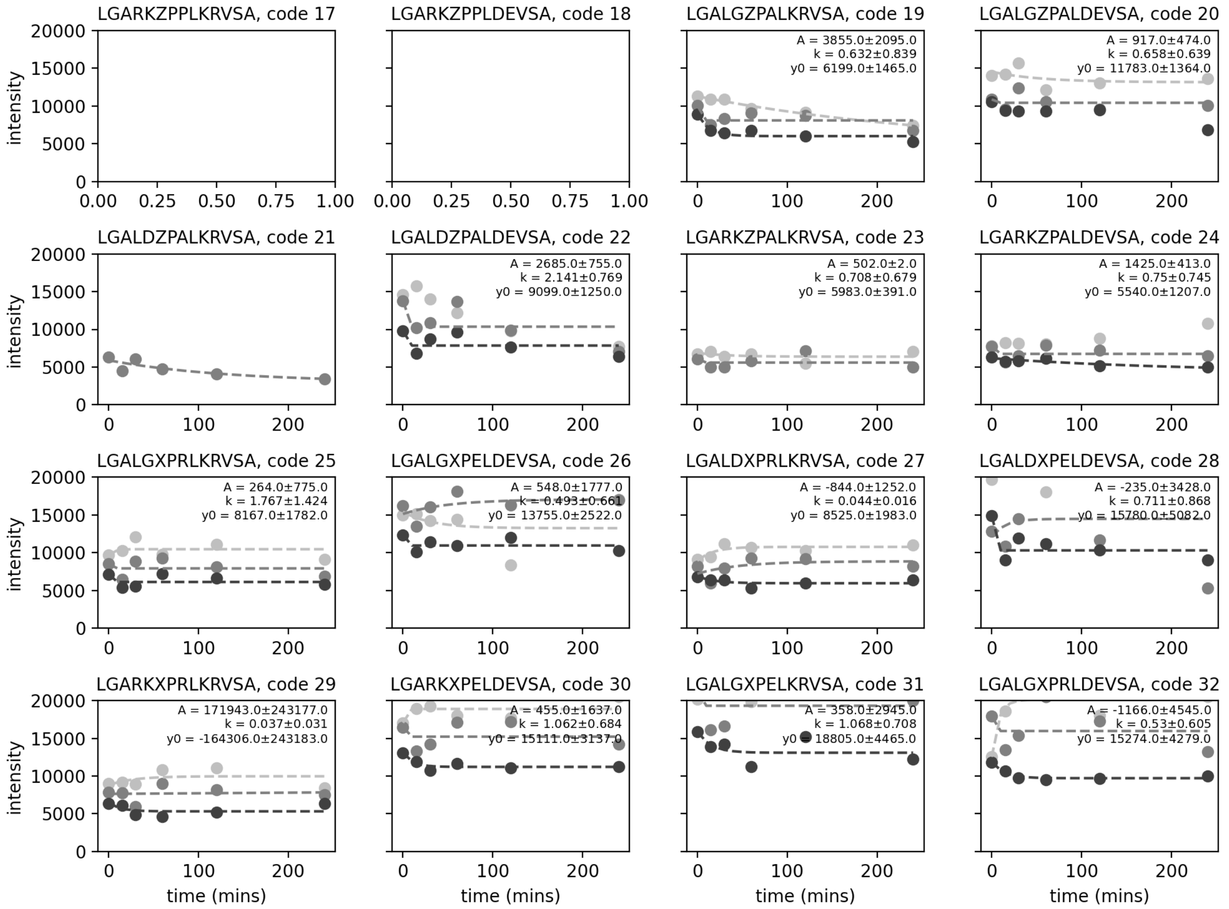

Appendix Figure S4 (continued).

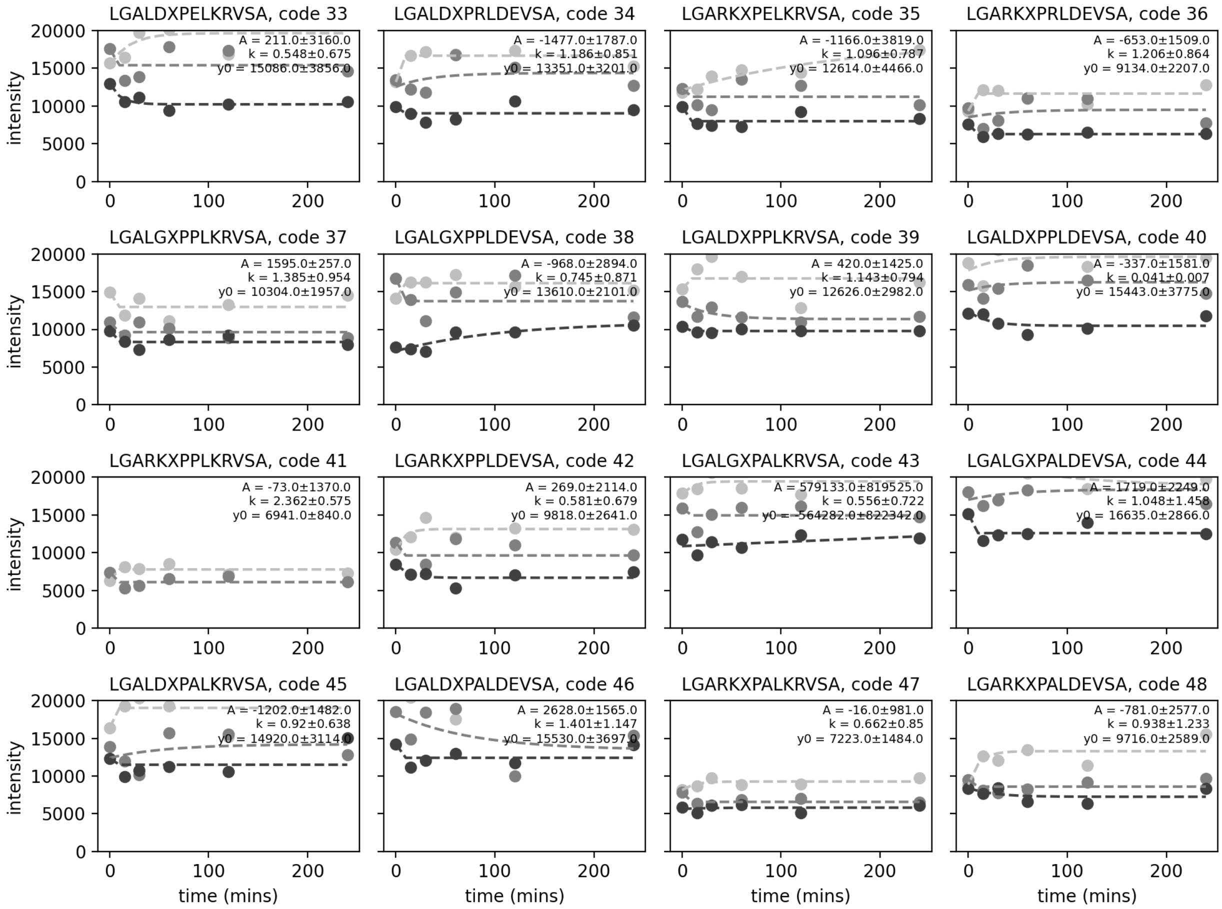

Appendix Figure S4 (continued).

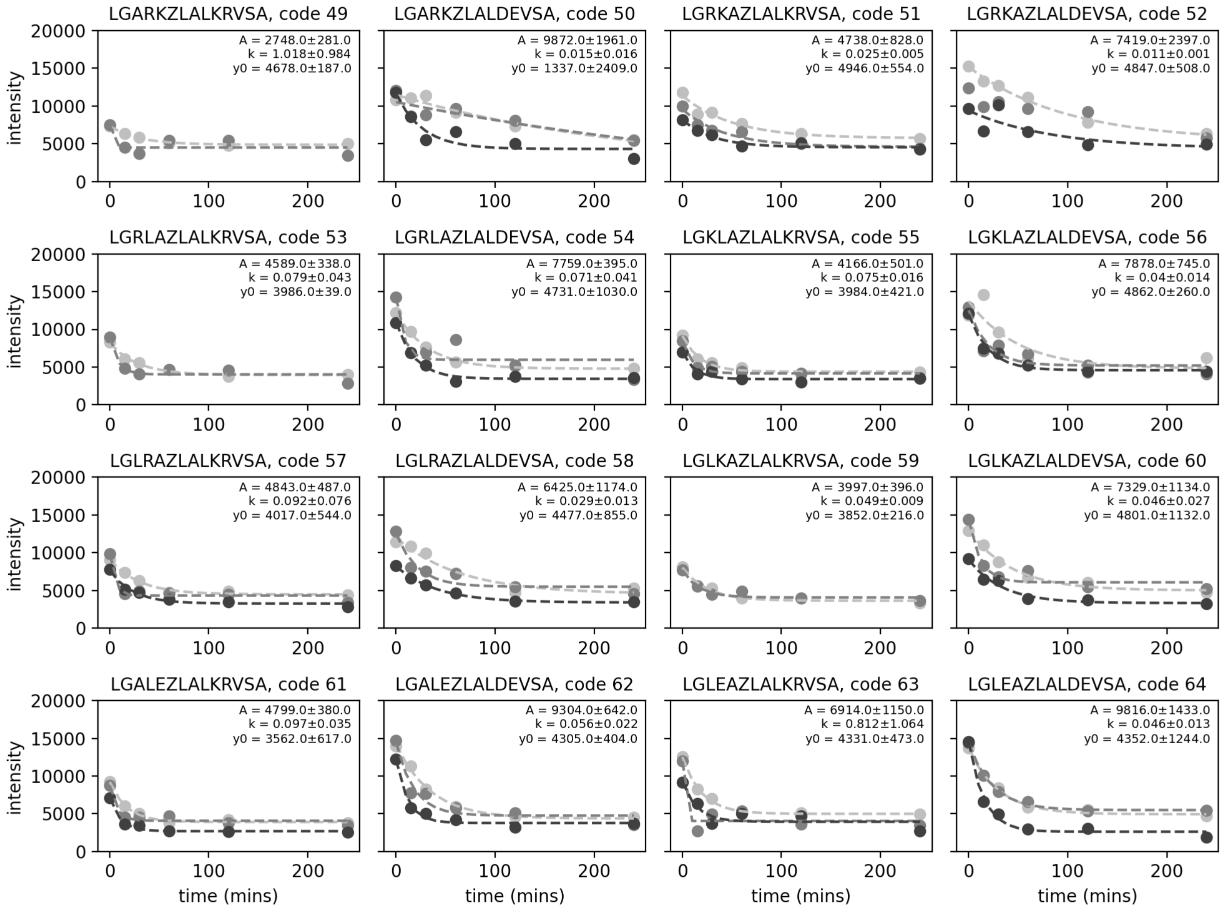

Appendix Figure S4 (continued).

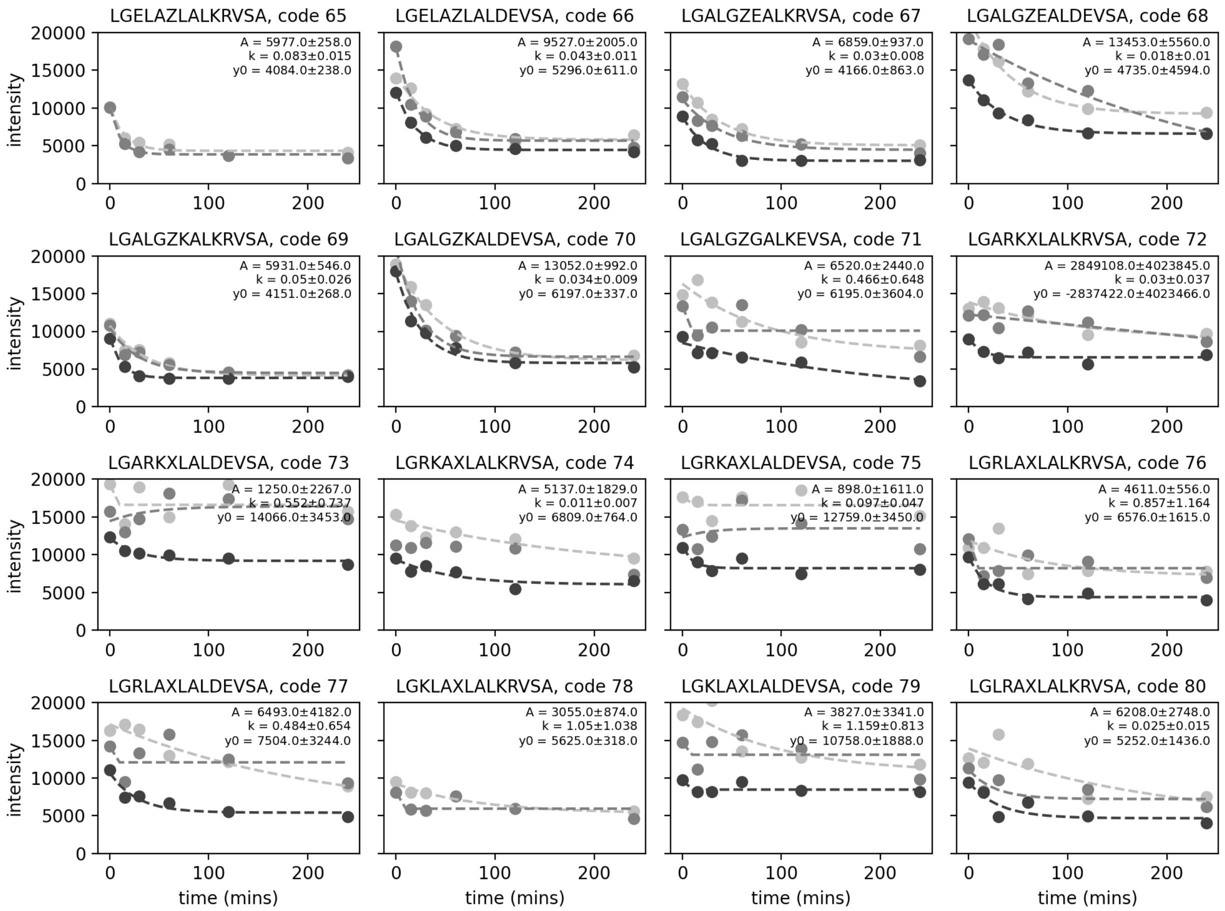

Appendix Figure S4 (continued).

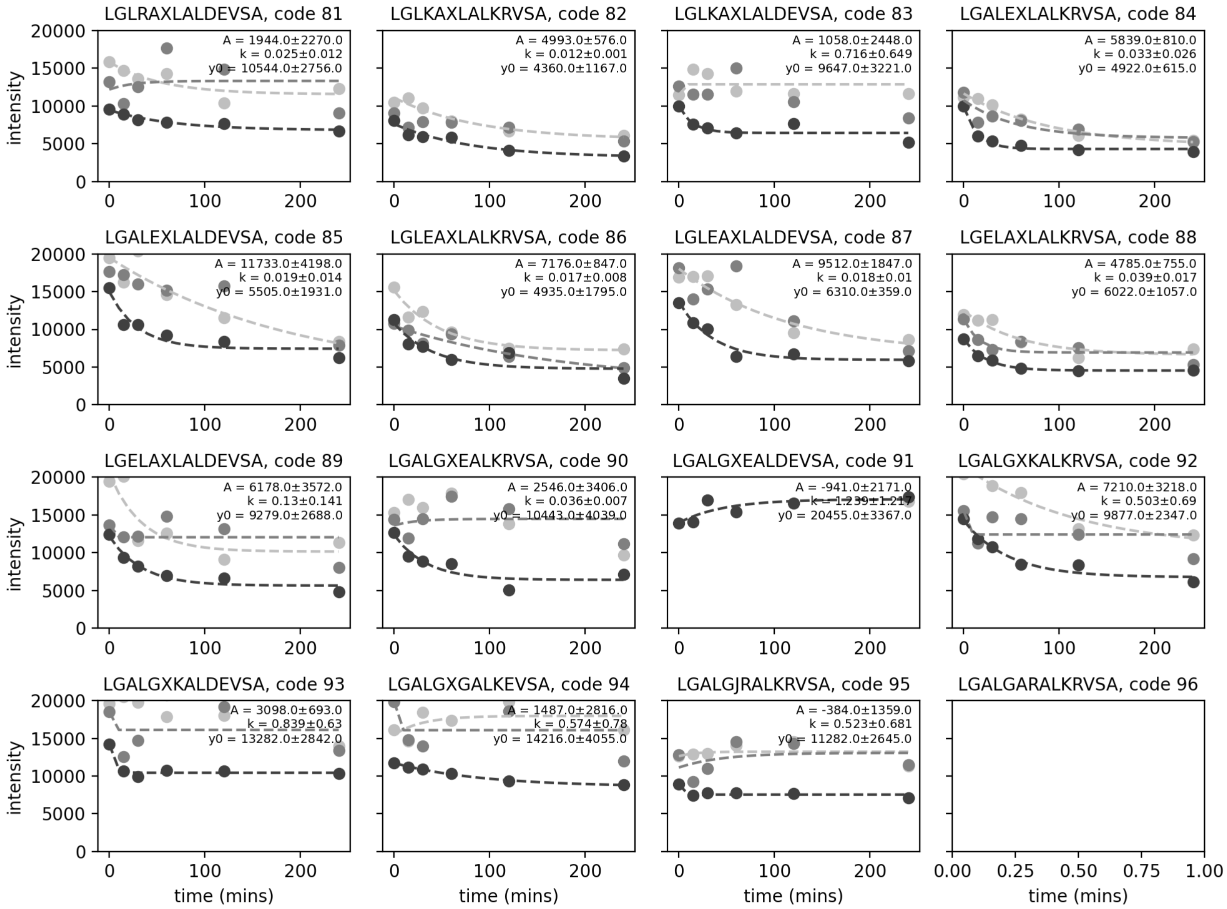

Appendix Figure S4 (continued).

A. (PP1)

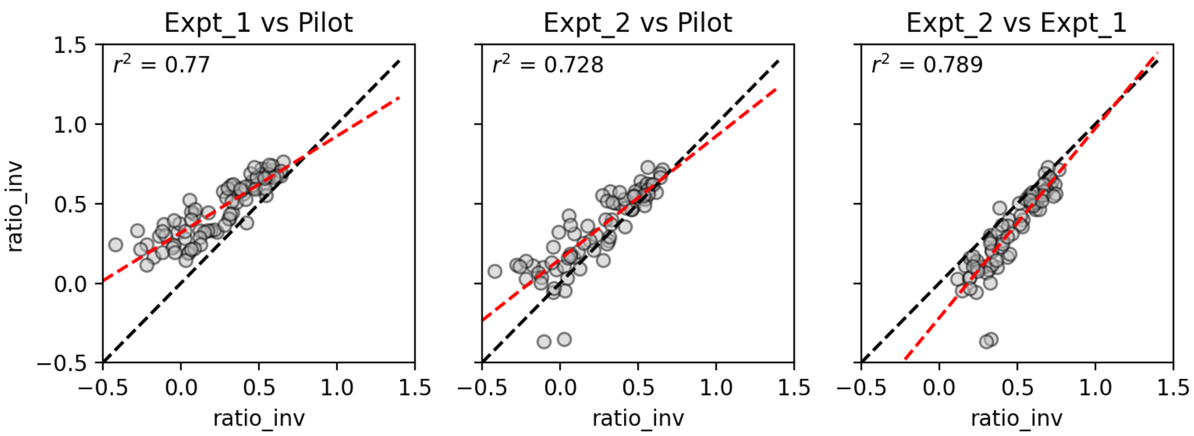

B. (B55)

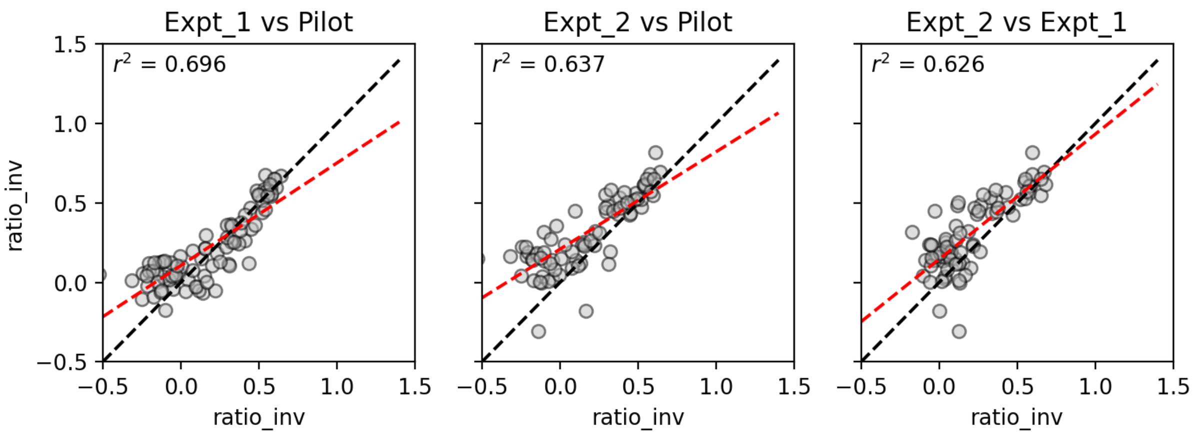

Appendix Figure S5. Pairwise comparisons of dephosphorylation scores ('ratio_inv') measured across 3 independent experimental replicates for PP1 **(A)** and PP2A-B55 (**B**). Each marker indicates the dephosphorylation score for a given peptide within a single experiment. Dashed black line signifies the identity line; red dashed line shows a linear regression; annotation specifies the Pearson correlation coefficient.

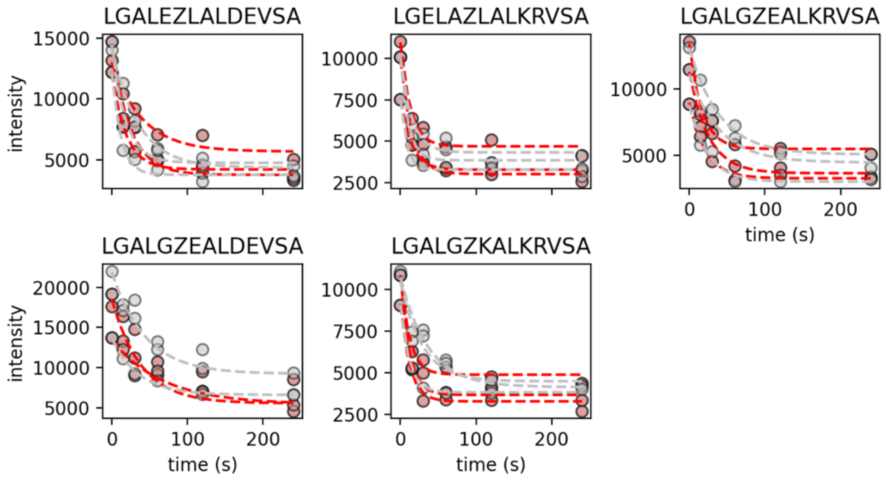

Appendix Figure S6. Example progress curves showing measured DyLight 650 fluorescence (normalized to starting intensity) over time for PP1 (red) and B55 (grey) proteins for 5 peptides. Dashed lines indicate single exponential fit and highlight differences in relative kinetics of dephosphorylation across the 2 proteins.

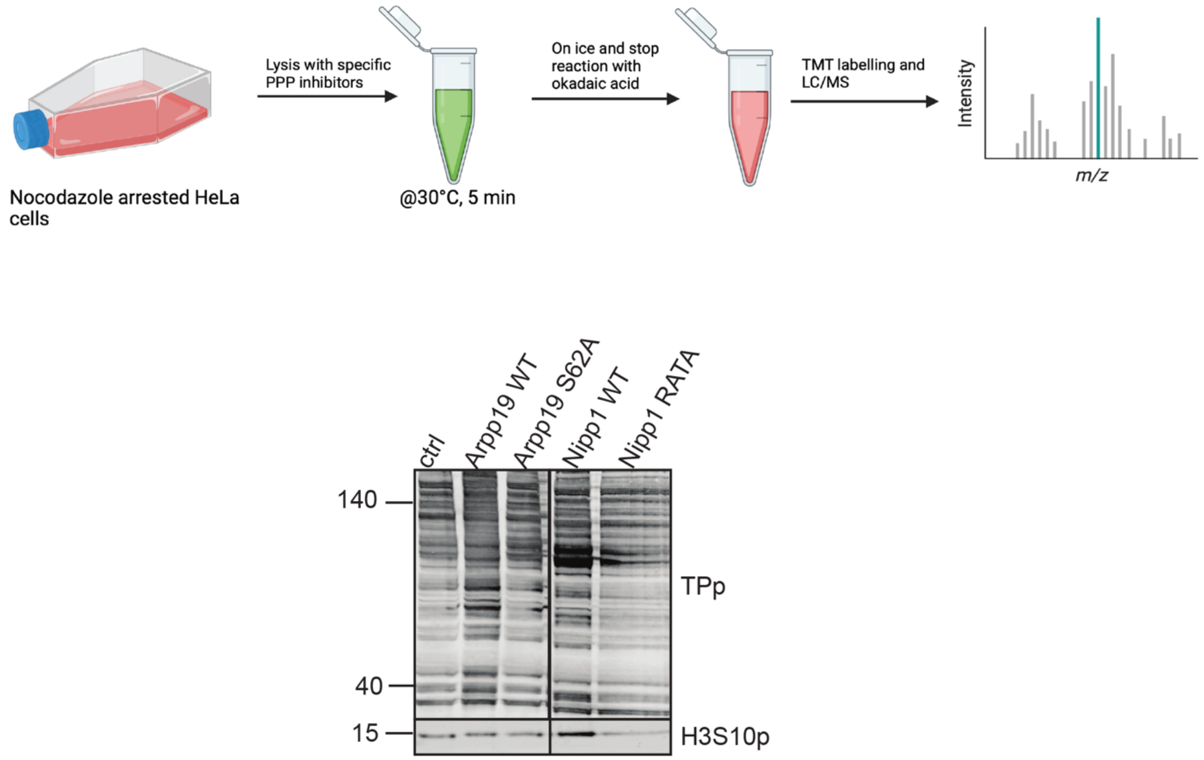

Appendix Figure S7. Experimental setup of PPP inhibition in mitotic lysate and westernblot of total cell extract treated as indicated and probed for total phosphor TP and H3S10p.

A

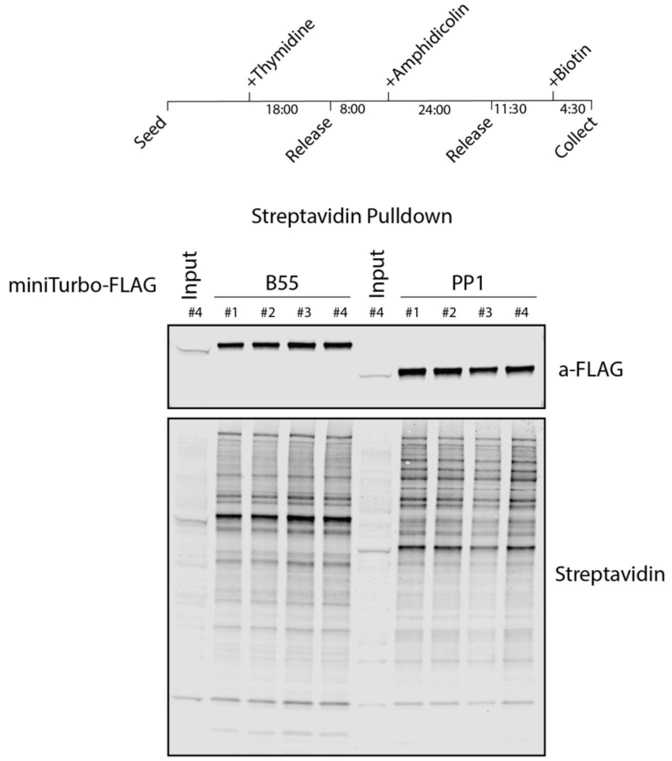

B

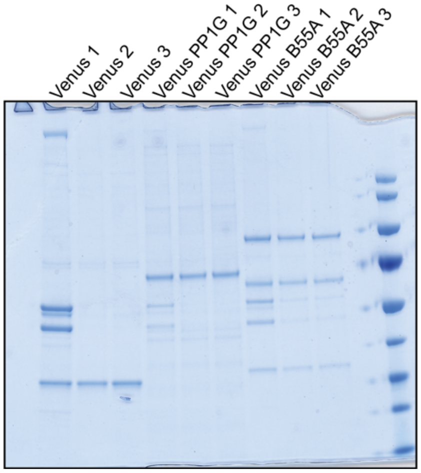

Appendix Figure S8. Interactomes for PP1 and PP2A-B55. A) Synchronisation protocol used for miniTurboID experiments and analysis of samples by westernblot. B) Affinity purified YFP-tagged samples analysed by coomassie gel.

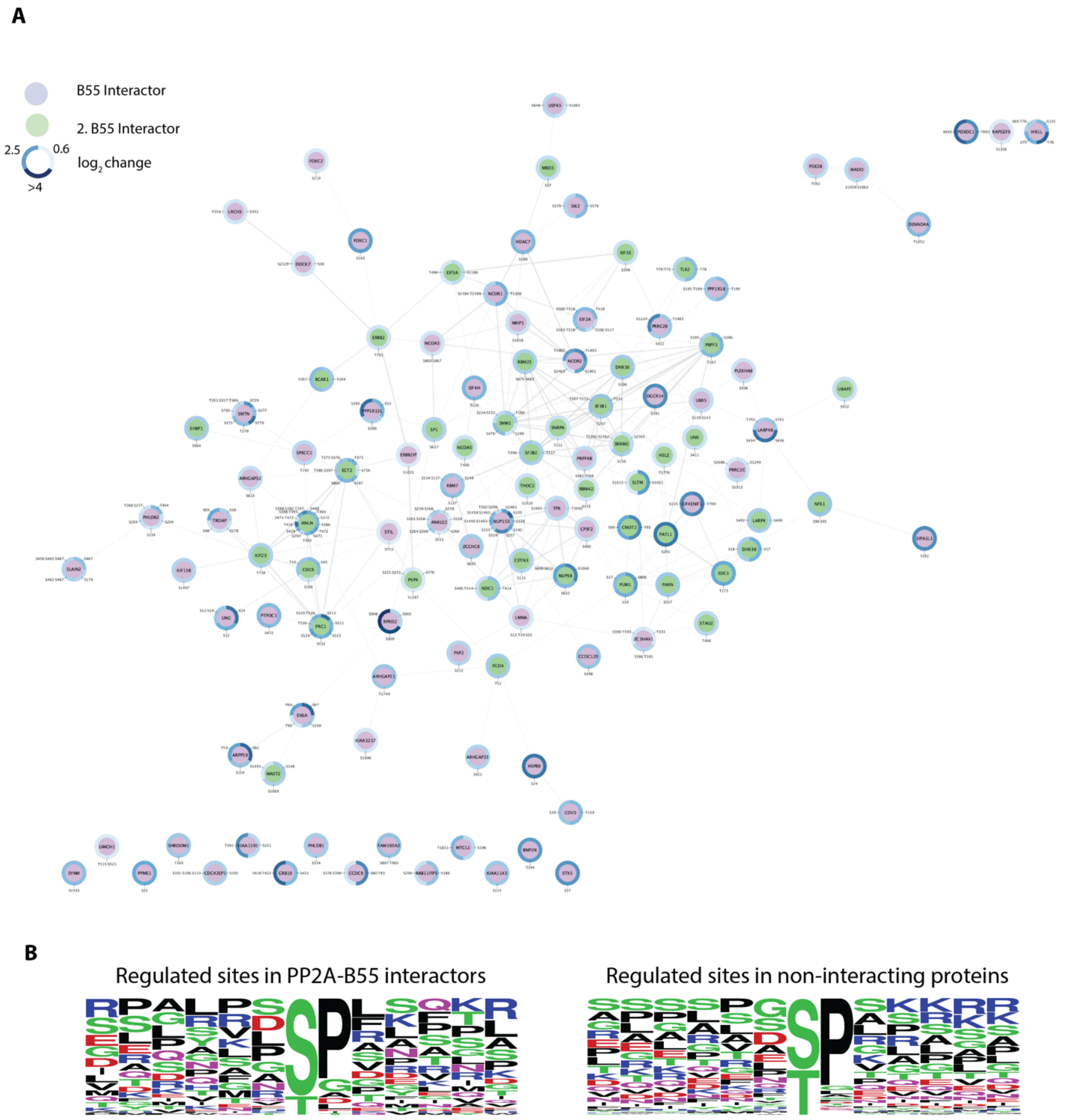

Appendix Figure S9. A) B55 dephosphorylation network obtained by integrating interactors and mapped regulated sites. B) Analysis of regulated sites in PP2A-B55 interactors and in non-interacting proteins.

Regulated sites in RVxF containing proteins   Regulated sites in proteins without RVxF motif

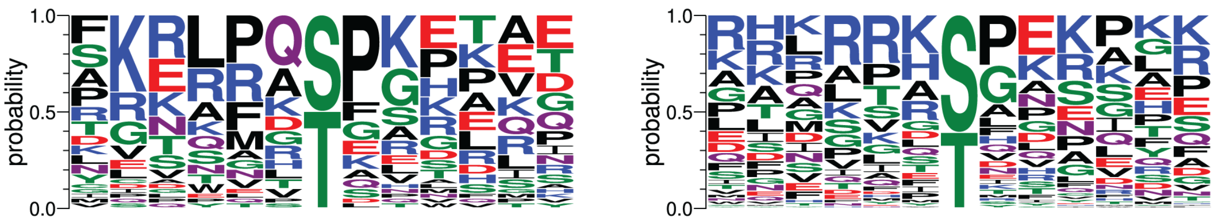

Appendix Figure S10. Analysis of phosphorylation sites regulated by PP1 and whether the sites where present in proteins with or without an RVxF motif.

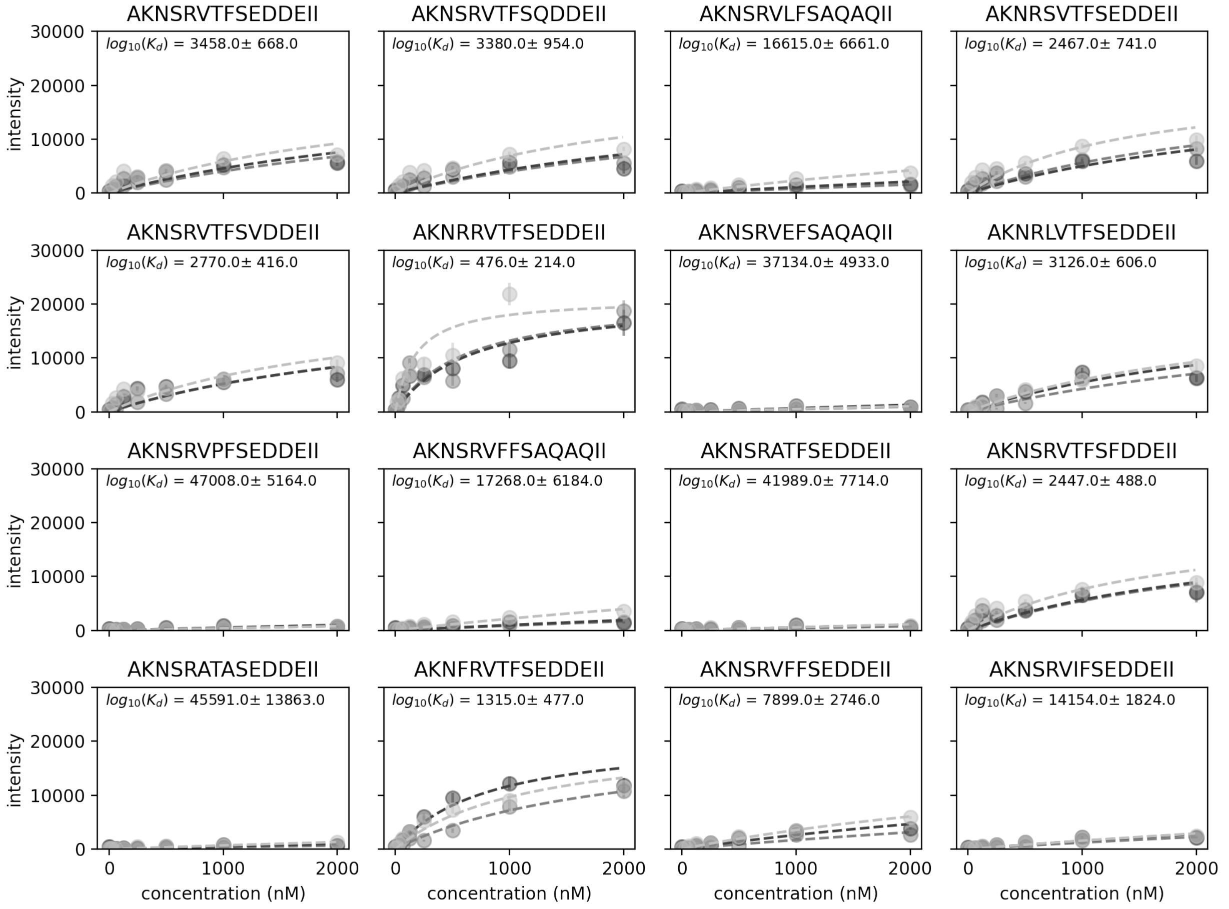

Appendix Figure S11. MRBLE-pep concentration-dependent peptide binding curves for PP1. Each shade (markers and fits) indicates a different experimental replicate; dashed lines indicate a Langmuir isotherm fit ($y = y_{max}*[PP1]/([PP1] + K_d)$); annotated values are the mean ± standard deviation across 3 independent experimental replicates.

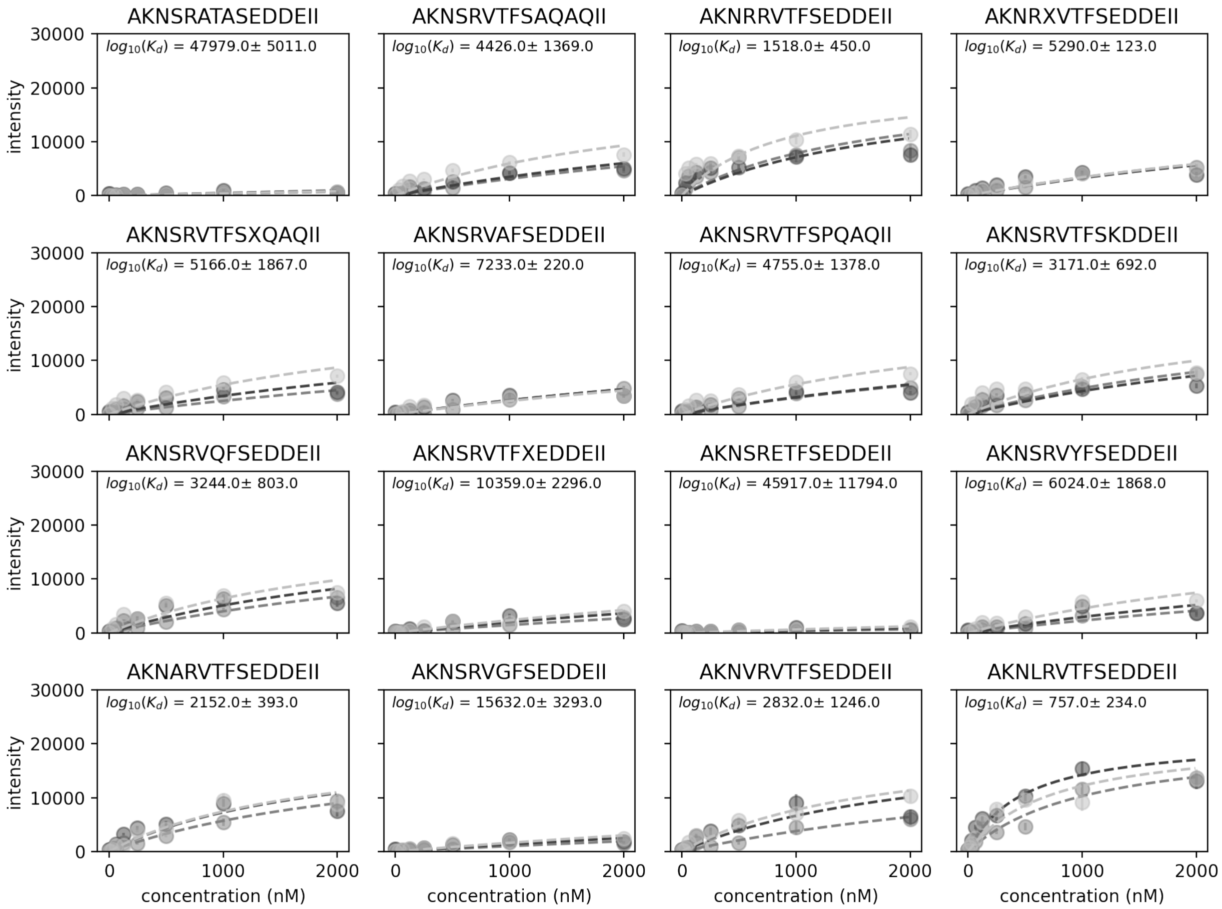

Appendix Figure S11 (continued).

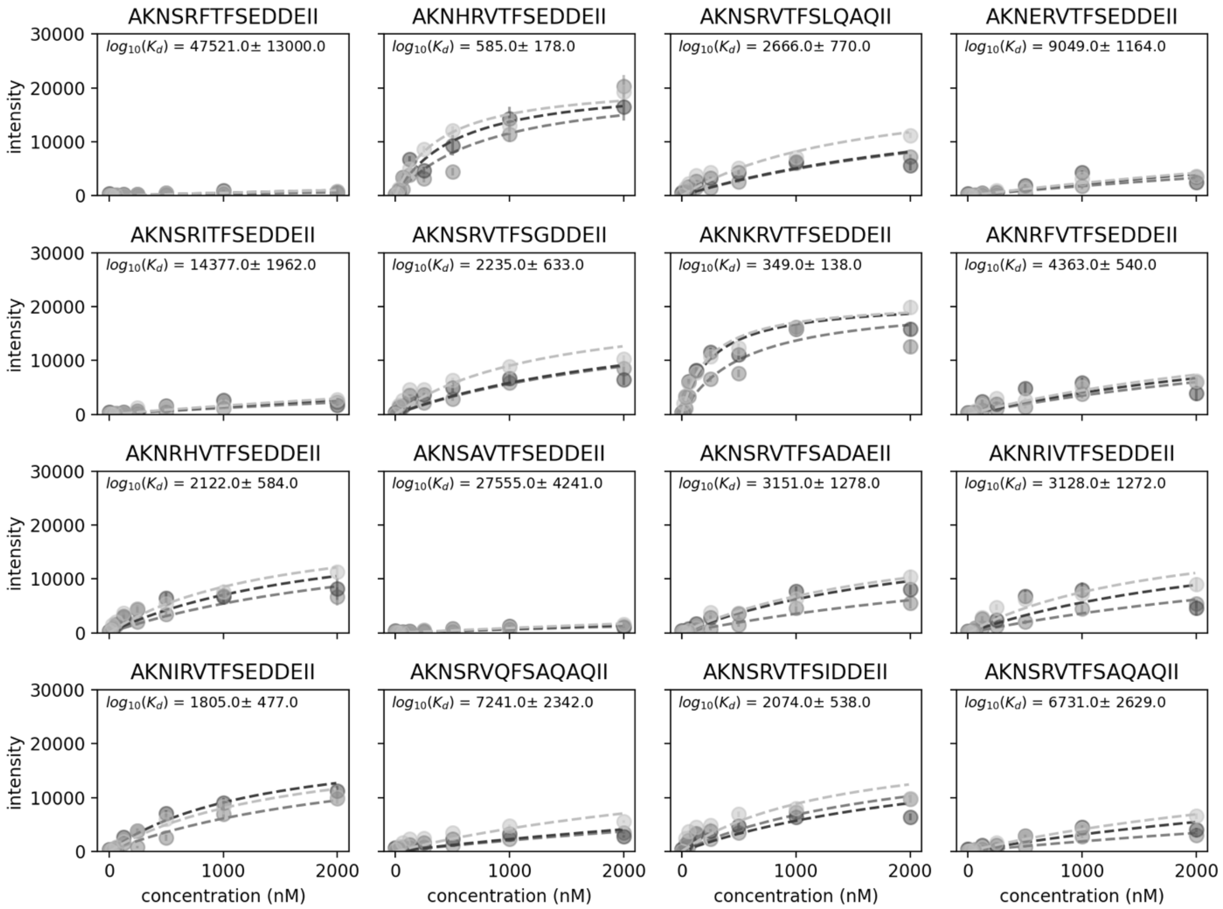

Appendix Figure S11 (continued).

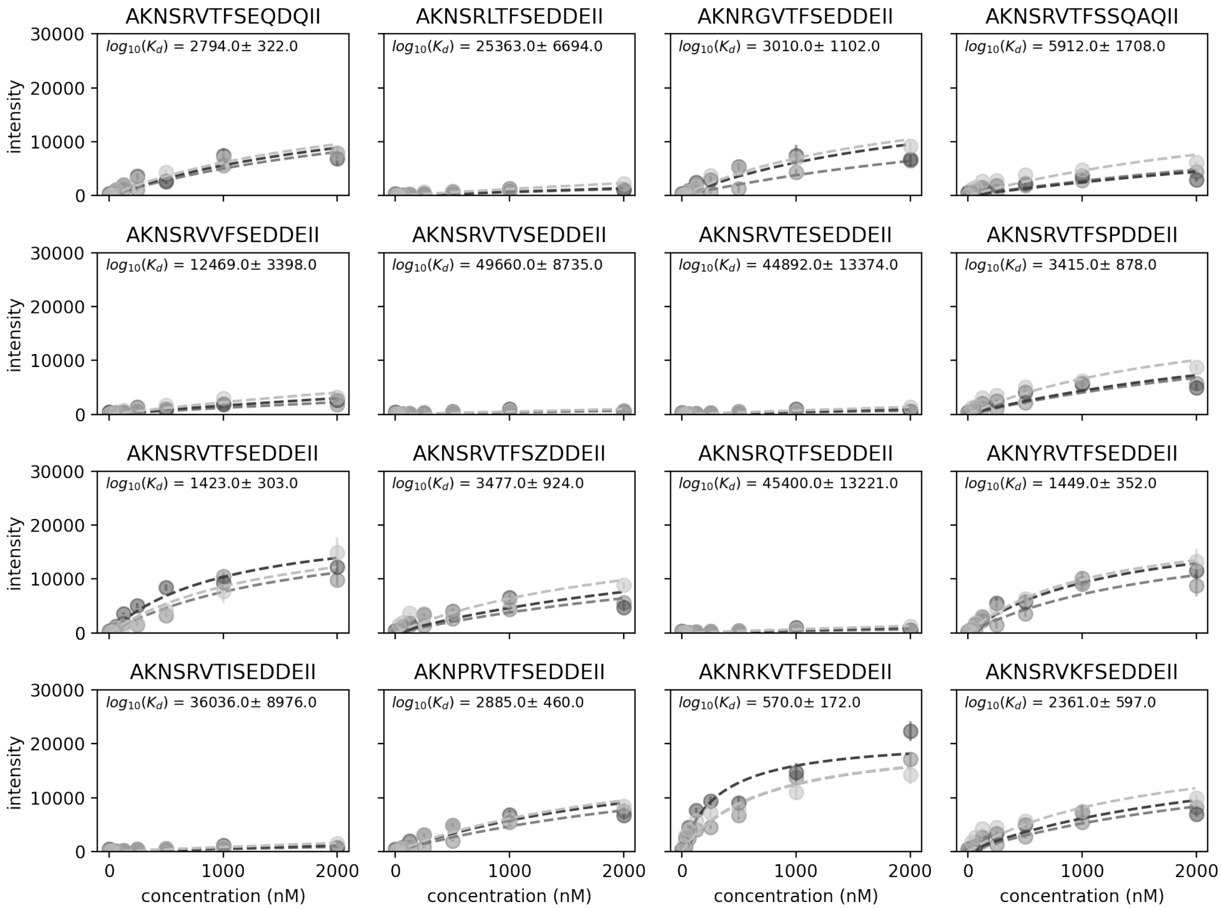

Appendix Figure S11 (continued).

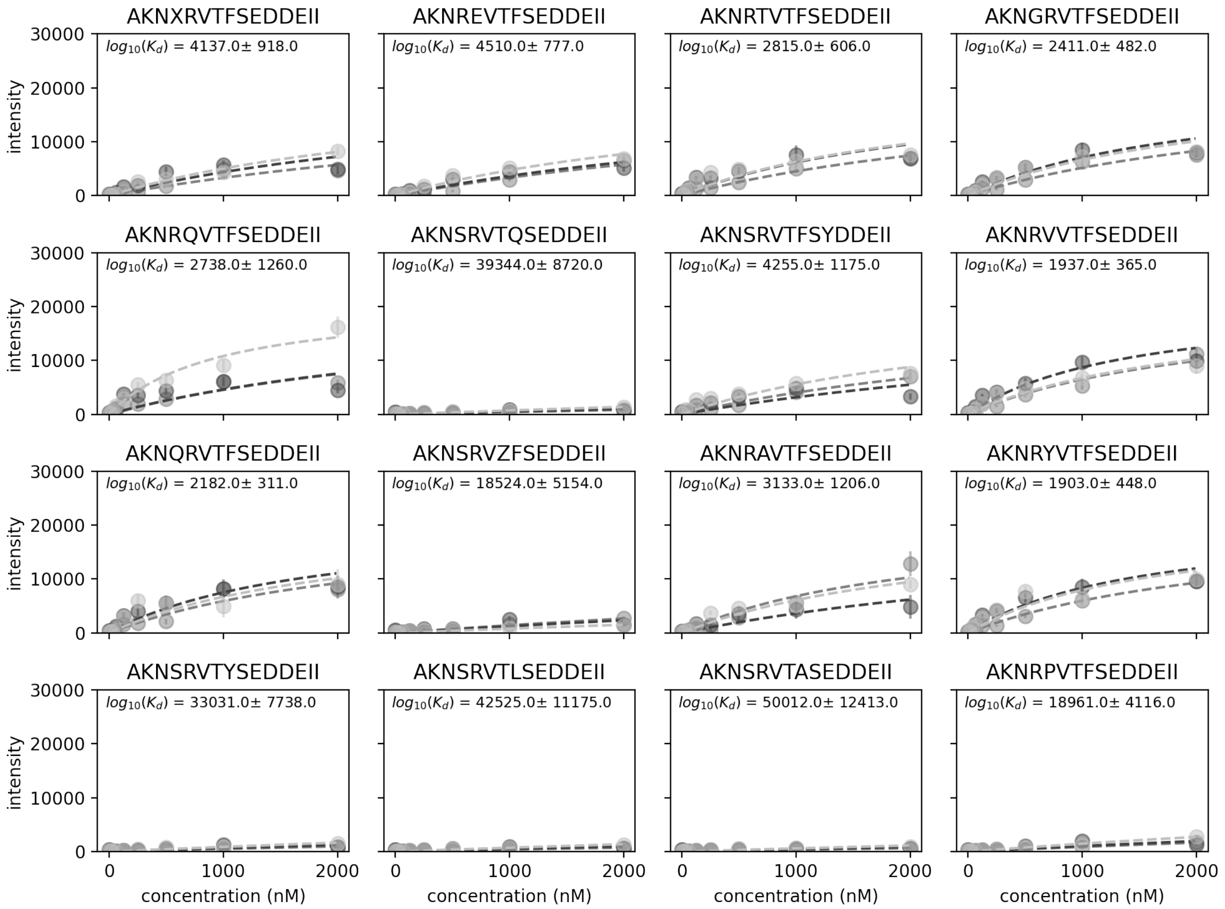

Appendix Figure S11 (continued).

A

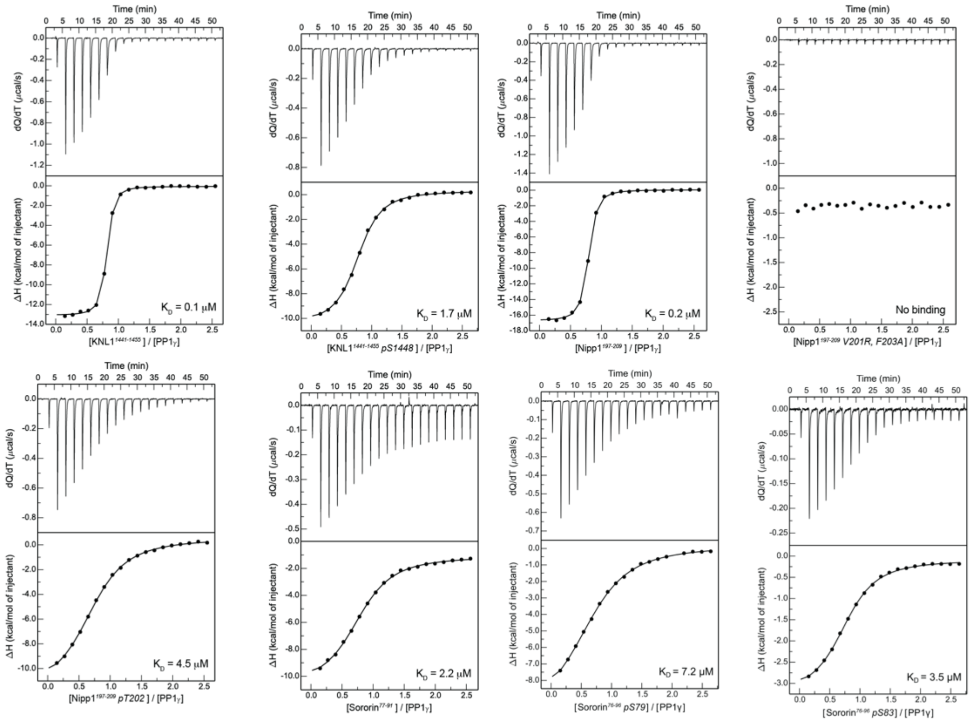

B

Impact of RVxF motif phosphorylation on PP1 binding

PP1γ complex	Peptide sequence	K_D (μM)
PP1γ/Nipp1$^{197-209}$	AKNSRVTFSEDDEIW	0.19 ± 0.01
PP1γ/Nipp1$^{197-209}$ pT202	AKNSRV(pT)FSEDDEIW	4.51 ± 0.20
PP1γ/Nipp1$^{197-209}$ V201R, F203A	AKNSRATASEDDEIW	No binding
PP1γ/Nipp1$^{197-209}$ V201R, pT202, F203A	AKNSRA(pT)ASEDDEIW	No binding
PP1γ/KNL1$^{1441-1455}$	KLNSKRVSFKLPKDQW	0.12 ± 0.01
PP1γ/KNL1$^{1441-1455}$ pS1448	KLNSKRV(pS)FKLPKDQW	1.73 ± 0.01
PP1γ/Sororin$^{77-91}$ pS83	RRSPRI(pS)FFLEKENEW	1.36 ± 0.01
PP1γ/Sororin$^{76-96}$	PRRSPRISFFLEKENEPPGREW	2.53 ± 0.11
PP1γ/Sororin$^{76-96}$ pS79	PRR(pS)PRISFFLEKENEPPGREW	7.24 ± 0.40
PP1γ/Sororin$^{76-96}$ pS83	PRRSPRI(pS)FFLEKENEPPGREW	3.46 ± 0.18

C

PP1 dependent phosphosites in RVxF motif

Protein	Modsite	RVXF	Position
NOC3L	S116	GQRVSFL	103, 110
CDCA5	S75, S83	SPRISFF	78, 85
PGM2	S165	GYKVYWD	172, 179
ARHGH	S383	VAKVSFP	371, 378
NOL10	S475	RFKVMFE	483, 490
SCAPE	S199	ARRSLNFG	196, 204
FRM4B	S940	SQRCLGFA	925, 933
TACC3	T59	AMKVTFQ	51, 58
KI67	S507	KRRRVSFG	501, 509
RBP2	T1944	NGRGVIFG	1931, 1939
RL24	T83	NPRQINWT	71, 79
PP1R8	S199, T202, S204	NSRVTFS	197, 204
PGM2L	S175	GYKVYWE	182, 189
RIF1	S2205	KVRRVSFA	2199, 2207
CASC5	S60, S1448	NSRRVSFA - NSKRVSFK	54,62 - 1442, 1450

D

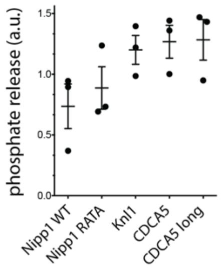

Appendix Figure S12. A) ITC curves for the indicated peptides using PP1γ. B) Table of Kd values for the indicated peptides. C) PP1 regulated sites identified in our mitotic exit screen that resides in RVxF sequences. D) Dephosphorylation of RVxF model peptides by PP1.
