# Supplementary figures and images for: Phosphatase specificity principles uncovered by MRBLE:Dephos and global substrate identification
Source: Mol Syst Biol. 2023 Nov 2;19(12):e11782. doi: 10.15252/msb.202311782 (PMC10698503; doi:10.15252/msb.202311782)

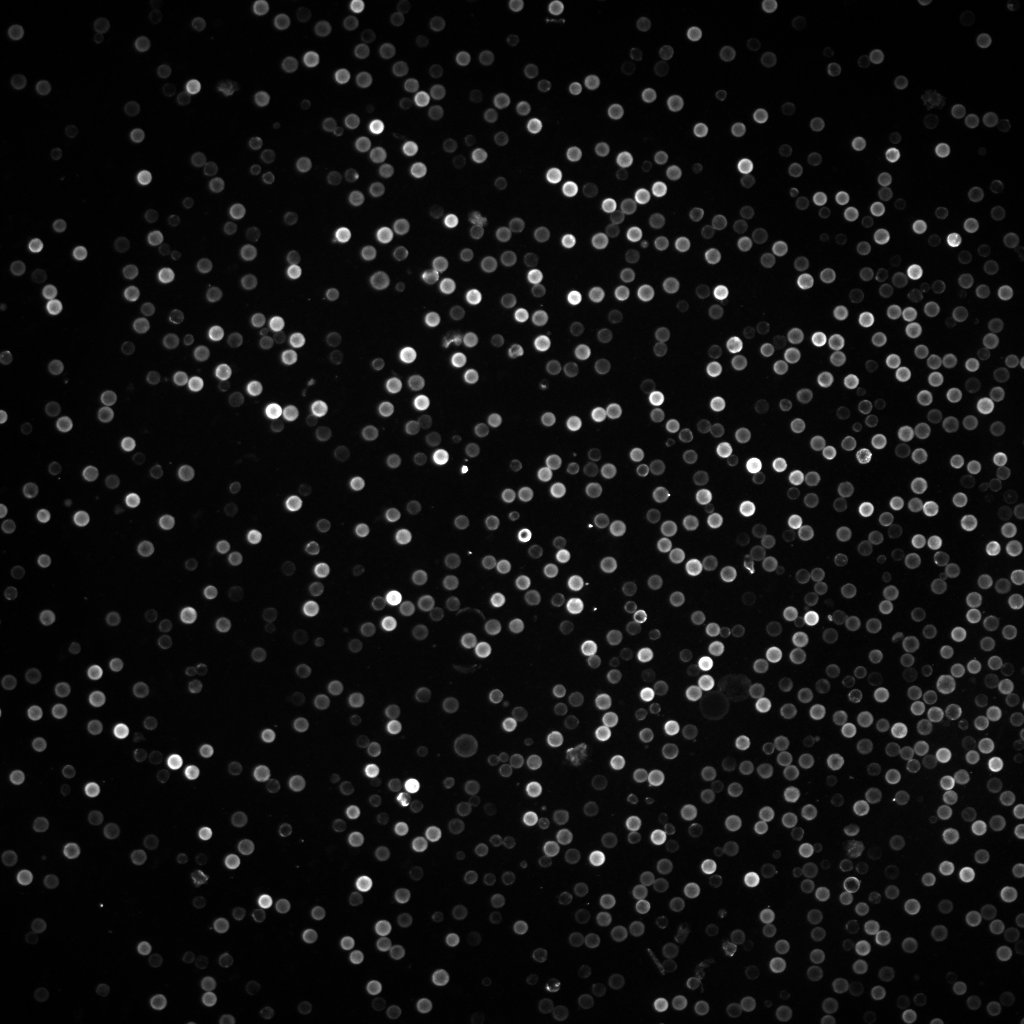

Supplement: Supplementary file 4 — Source Data for Figure 1 [file MSB-19-e11782-s002.zip › Figure 1/1B/Bead_Images/20200911_Lambda_17_MMStack_Pos0.ome-0003.tif]

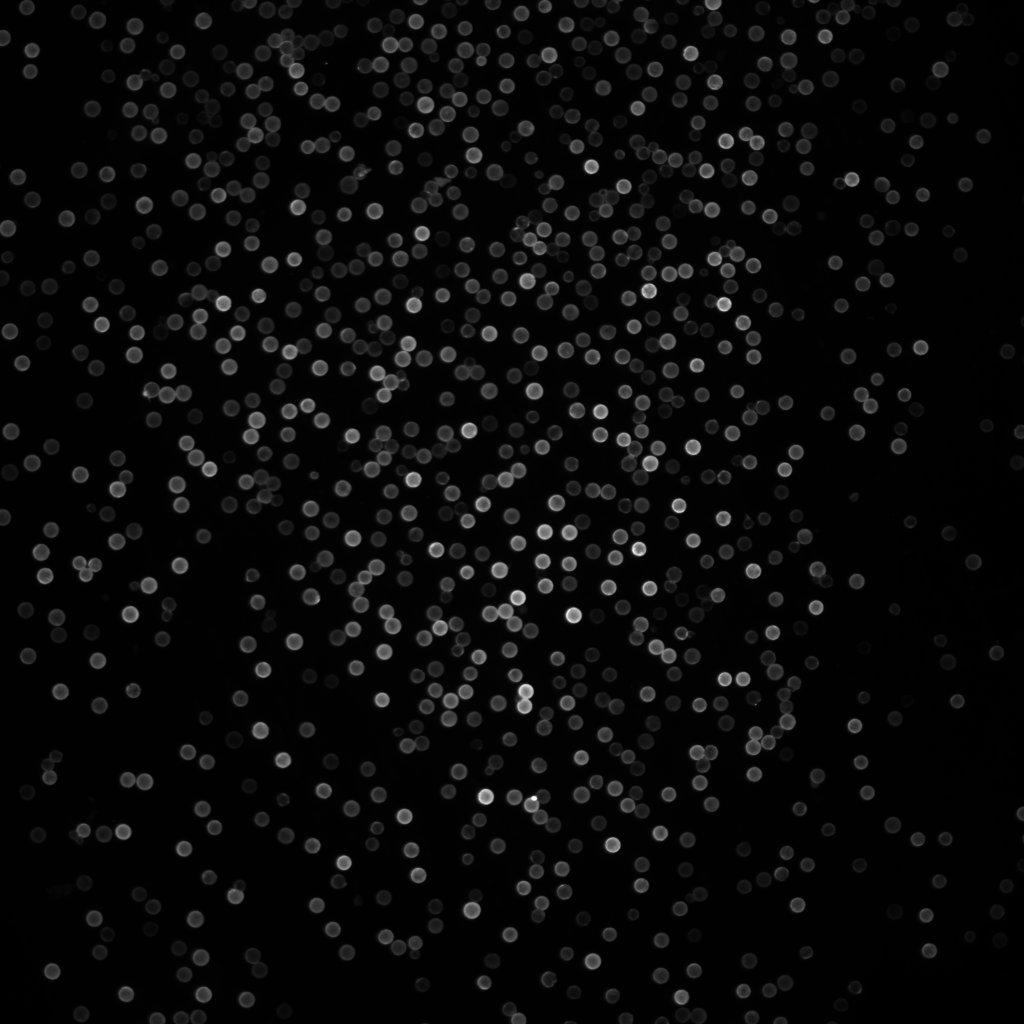

Supplement: Supplementary file 4 — Source Data for Figure 1 [file MSB-19-e11782-s002.zip › Figure 1/1B/Bead_Images/20200911_A_Bckg_9_MMStack_Pos0.ome-0003.jpg]

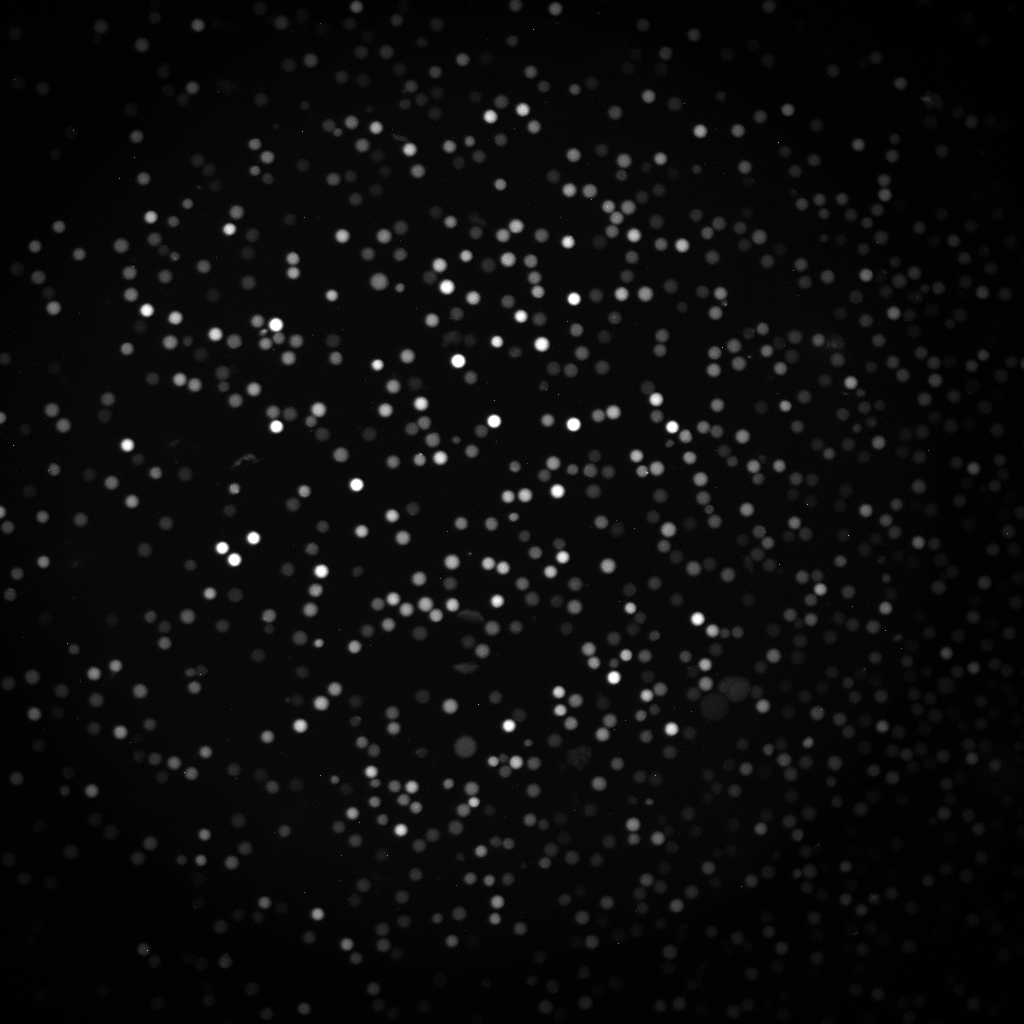

Supplement: Supplementary file 4 — Source Data for Figure 1 [file MSB-19-e11782-s002.zip › Figure 1/1B/Bead_Images/20200911_Lambda_17_MMStack_Pos0.ome-0006.tif]

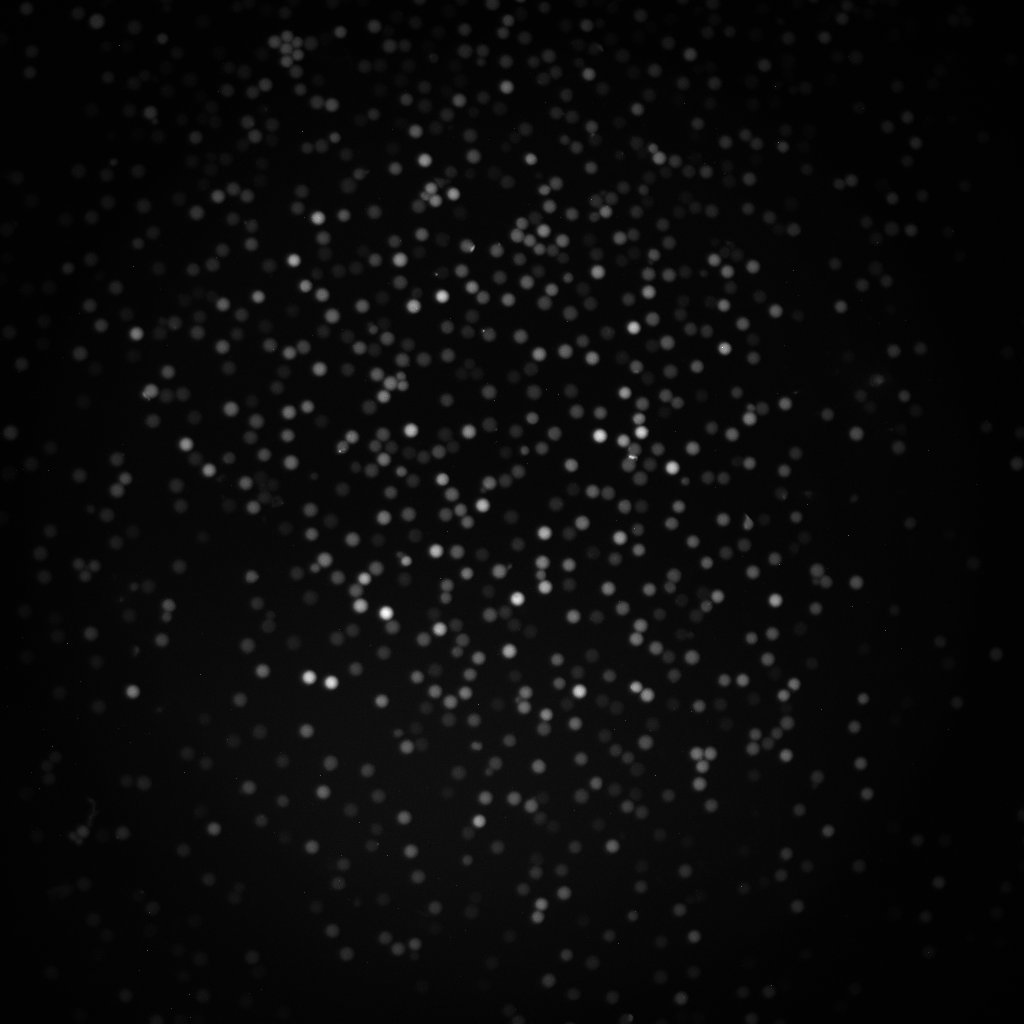

Supplement: Supplementary file 4 — Source Data for Figure 1 [file MSB-19-e11782-s002.zip › Figure 1/1B/Bead_Images/20200911_A_Bckg_9_MMStack_Pos0.ome-0006.jpg]

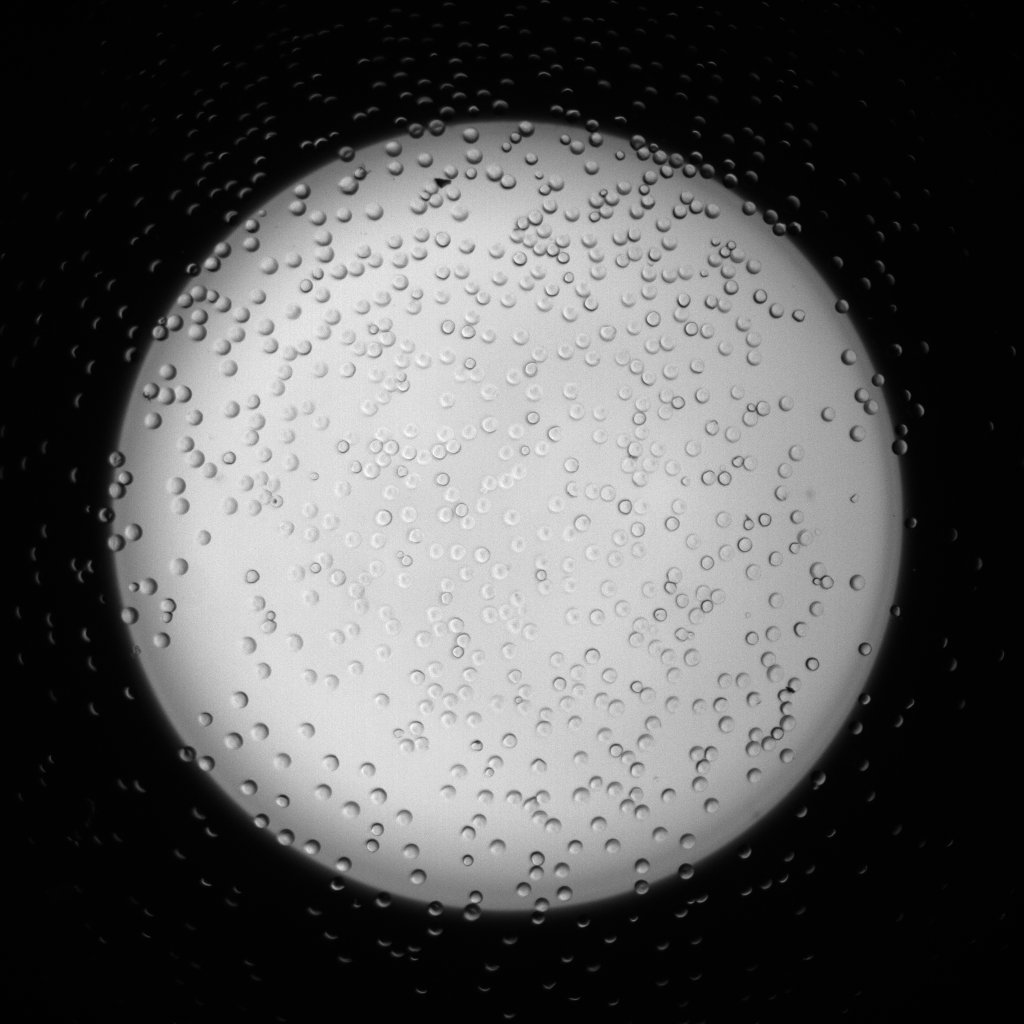

Supplement: Supplementary file 4 — Source Data for Figure 1 [file MSB-19-e11782-s002.zip › Figure 1/1B/Bead_Images/20200911_A_Bckg_9_MMStack_Pos0.ome-0004.jpg]

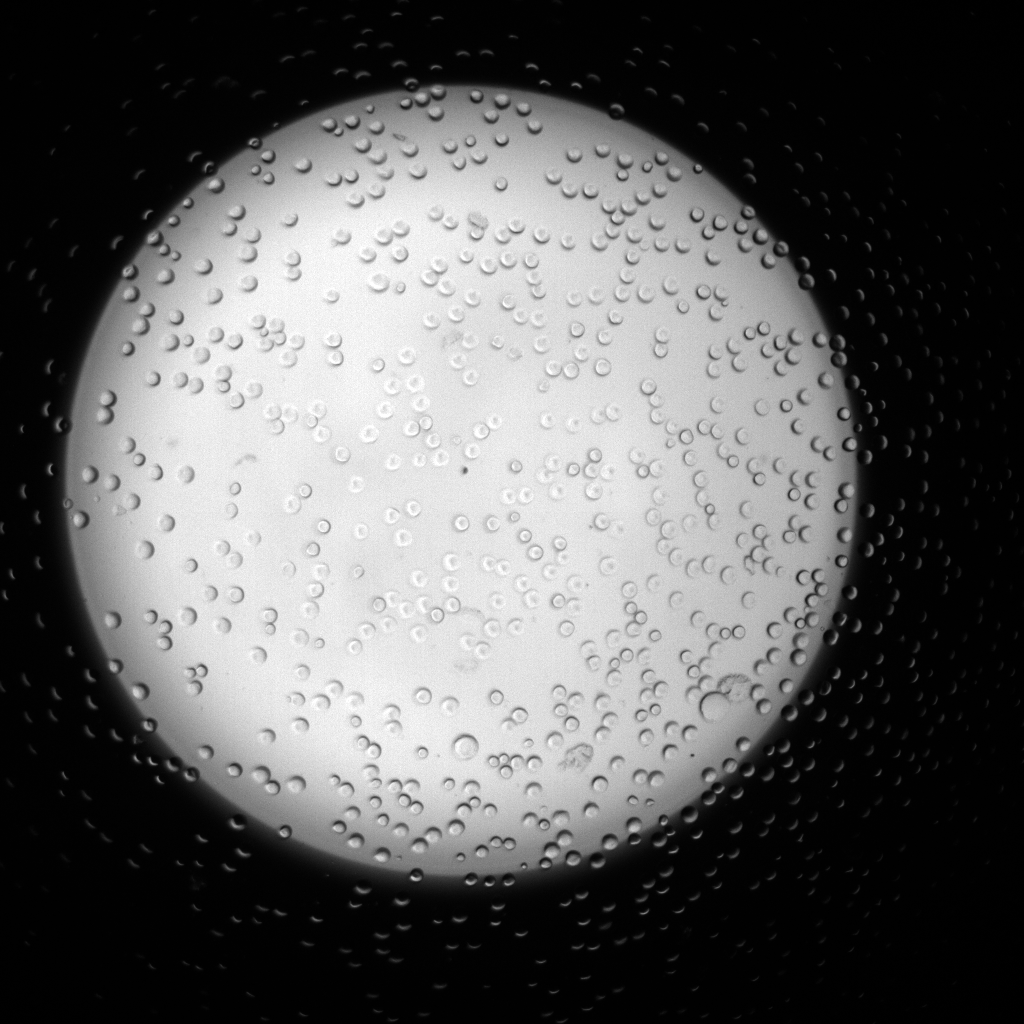

Supplement: Supplementary file 4 — Source Data for Figure 1 [file MSB-19-e11782-s002.zip › Figure 1/1B/Bead_Images/20200911_Lambda_17_MMStack_Pos0.ome-0004.tif]

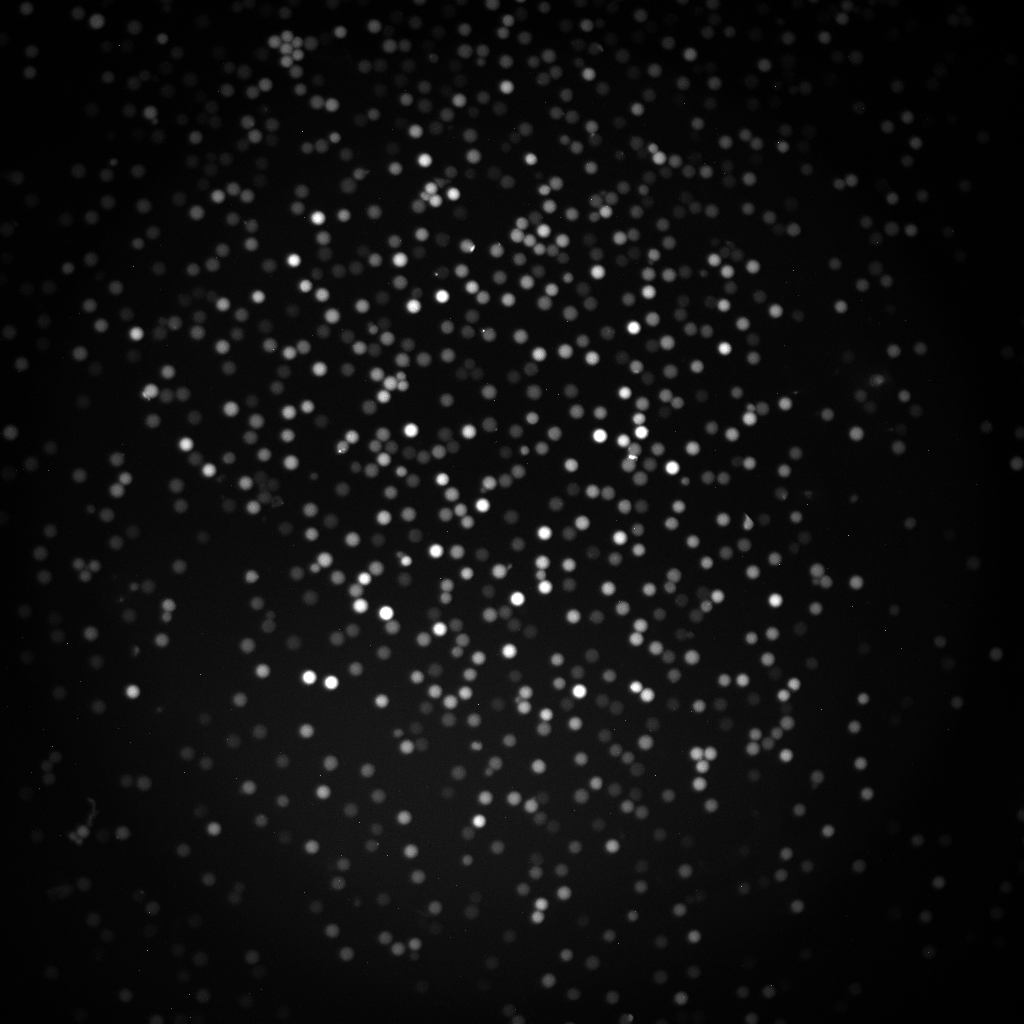

Supplement: Supplementary file 4 — Source Data for Figure 1 [file MSB-19-e11782-s002.zip › Figure 1/1B/Bead_Images/20200911_A_Bckg_9_MMStack_Pos0.ome-0006.tif]

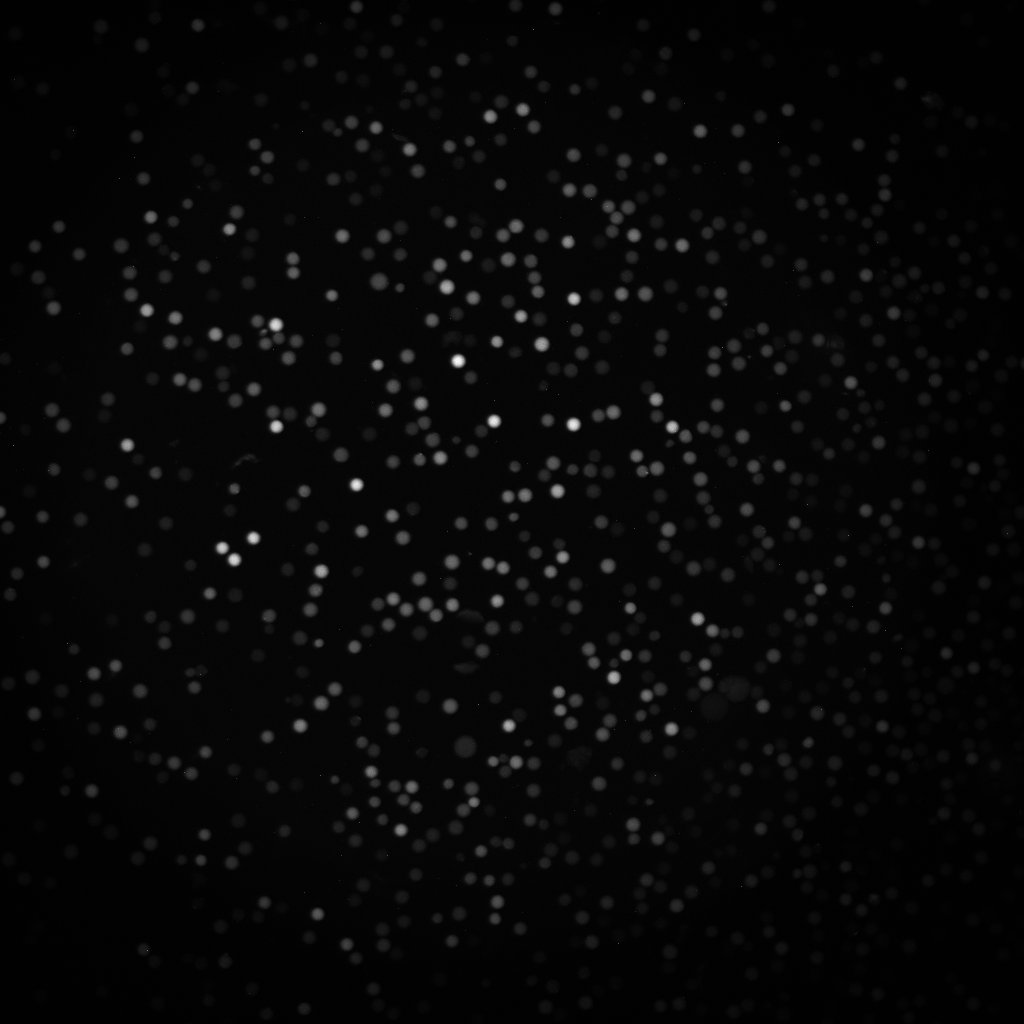

Supplement: Supplementary file 4 — Source Data for Figure 1 [file MSB-19-e11782-s002.zip › Figure 1/1B/Bead_Images/20200911_Lambda_17_MMStack_Pos0.ome-0006.jpg]

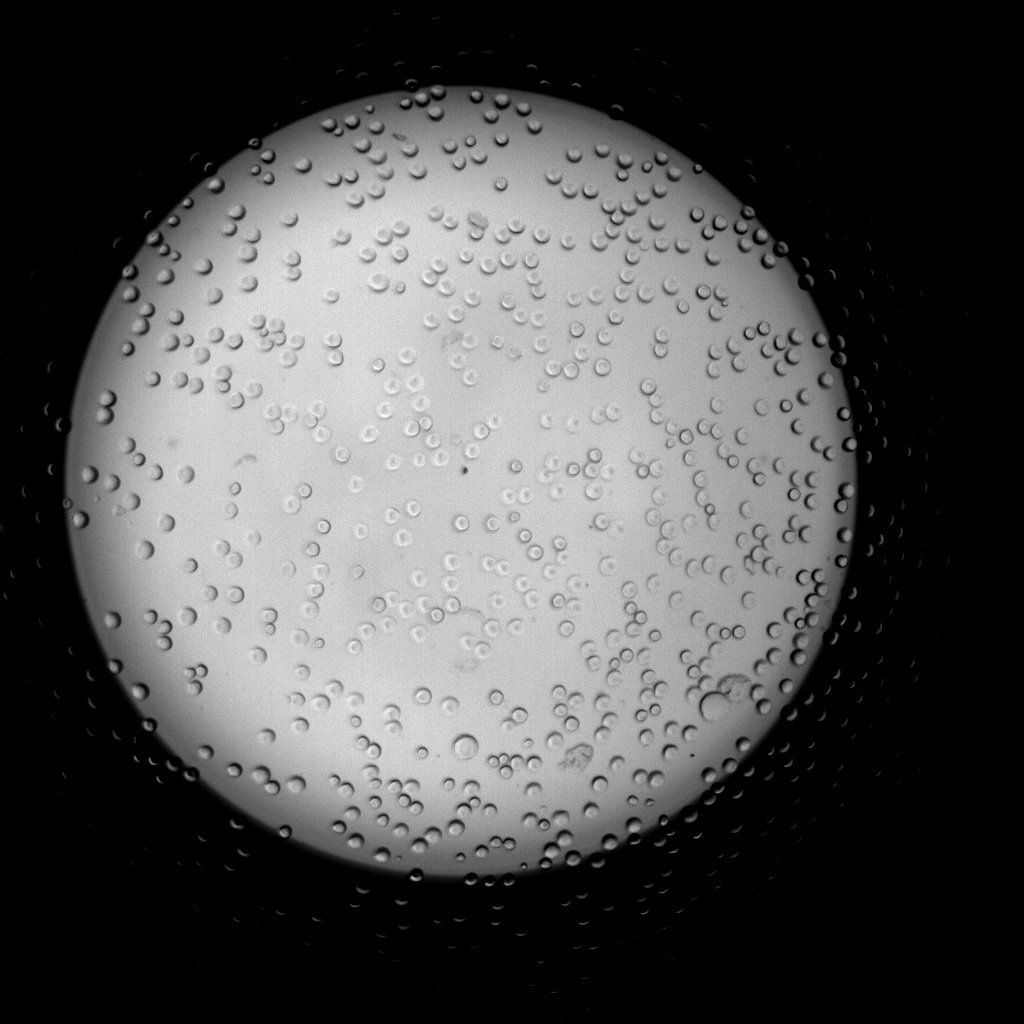

Supplement: Supplementary file 4 — Source Data for Figure 1 [file MSB-19-e11782-s002.zip › Figure 1/1B/Bead_Images/20200911_Lambda_17_MMStack_Pos0.ome-0004.jpg]

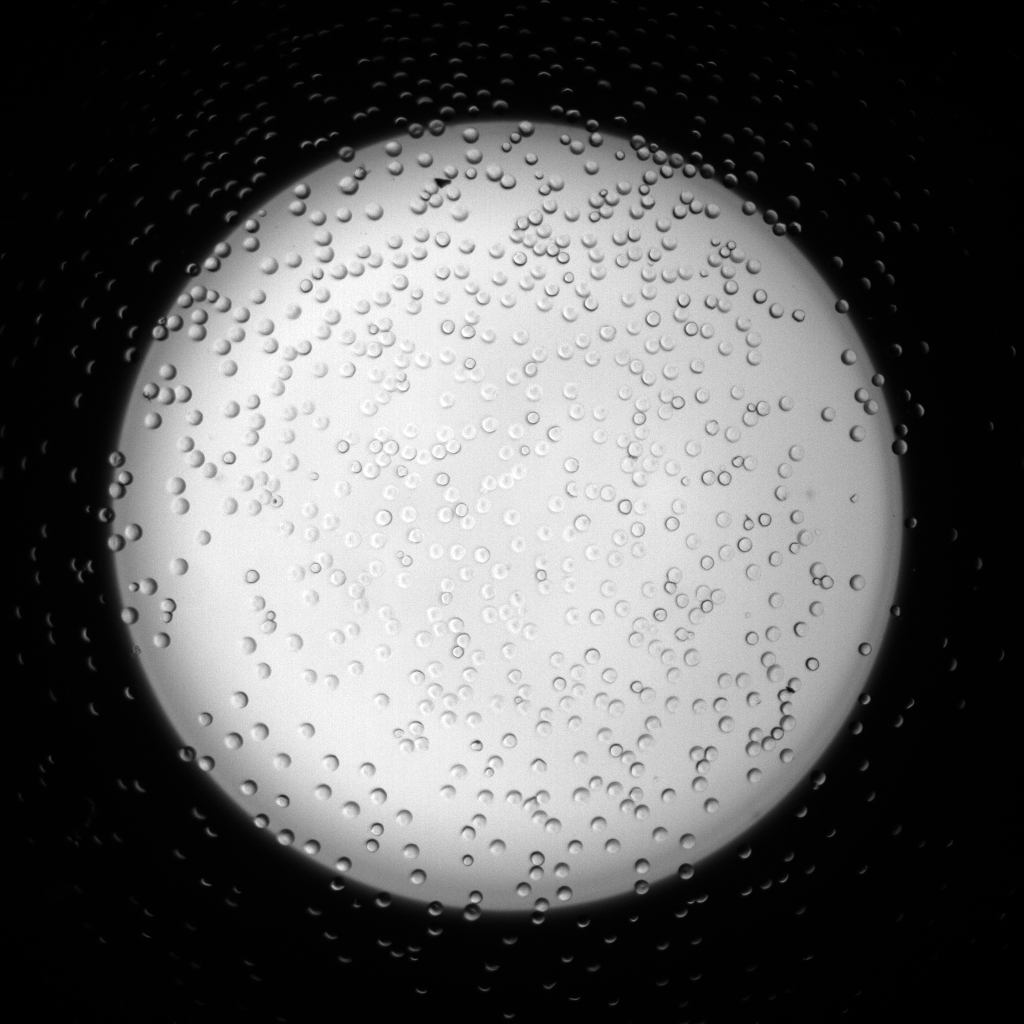

Supplement: Supplementary file 4 — Source Data for Figure 1 [file MSB-19-e11782-s002.zip › Figure 1/1B/Bead_Images/20200911_A_Bckg_9_MMStack_Pos0.ome-0004.tif]

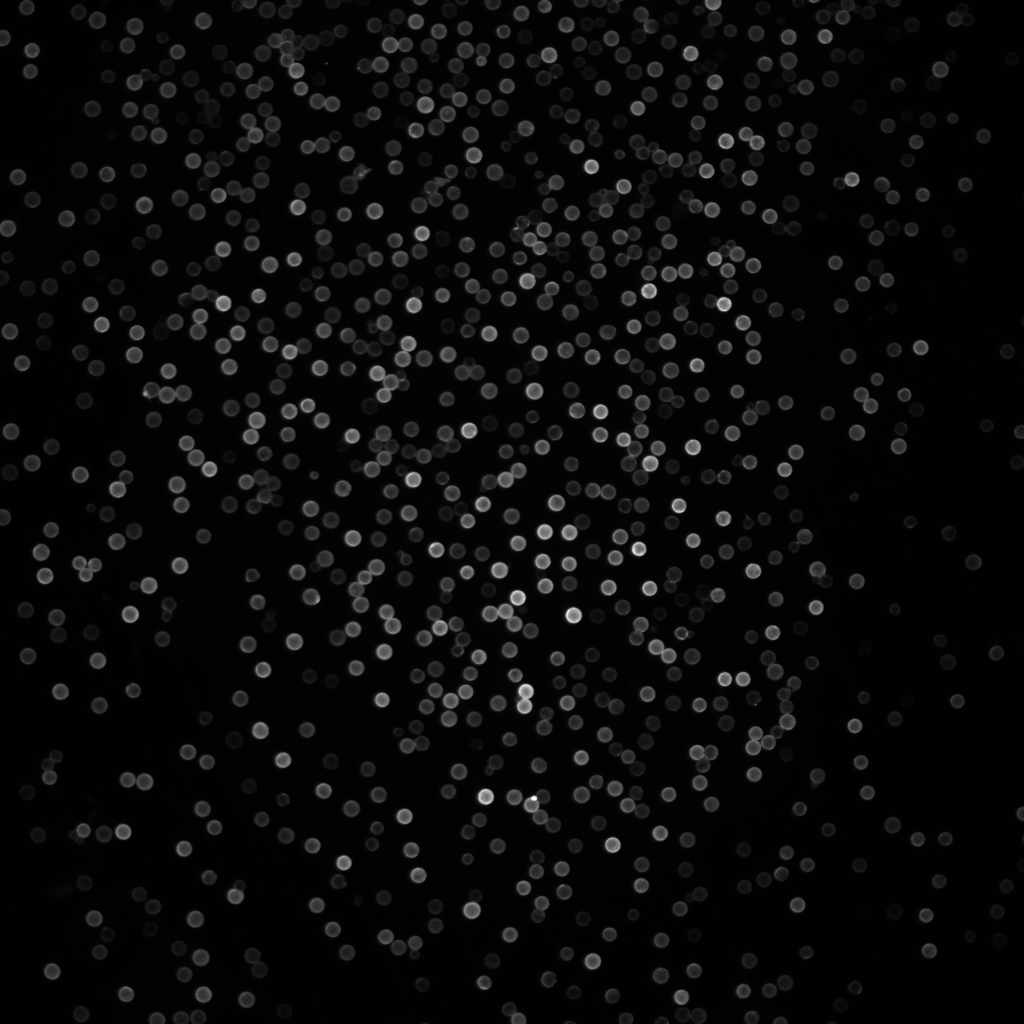

Supplement: Supplementary file 4 — Source Data for Figure 1 [file MSB-19-e11782-s002.zip › Figure 1/1B/Bead_Images/20200911_A_Bckg_9_MMStack_Pos0.ome-0003.tif]

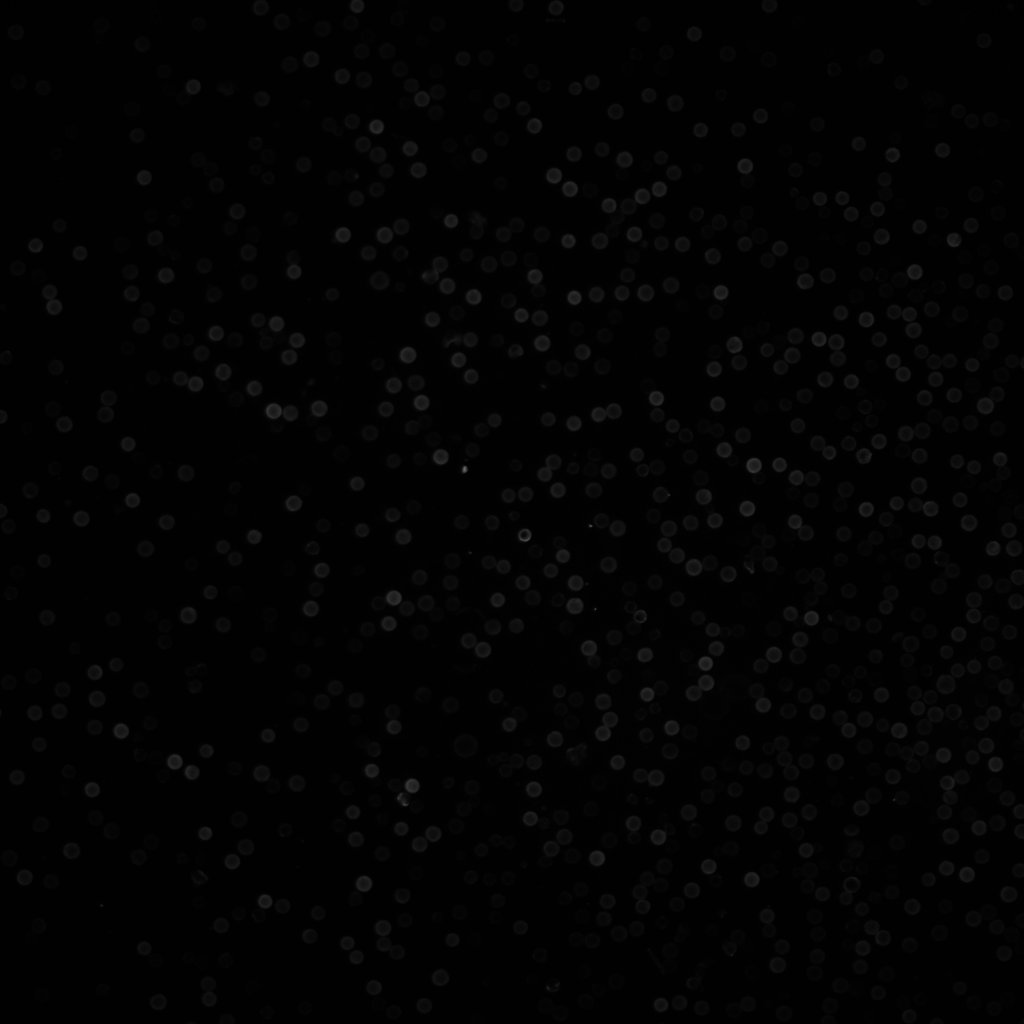

Supplement: Supplementary file 4 — Source Data for Figure 1 [file MSB-19-e11782-s002.zip › Figure 1/1B/Bead_Images/20200911_Lambda_17_MMStack_Pos0.ome-0003.jpg]

Uncropped INCENP phosphorylation assay

P32

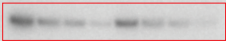

Coomassie

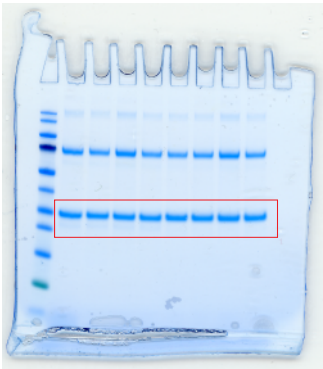

Supplement: Supplementary file 5 — Source Data for Figure 3 [file MSB-19-e11782-s004.zip › Figure 3/3C/Uncropped_INCENP Phosphorylation assayai copy.pdf]

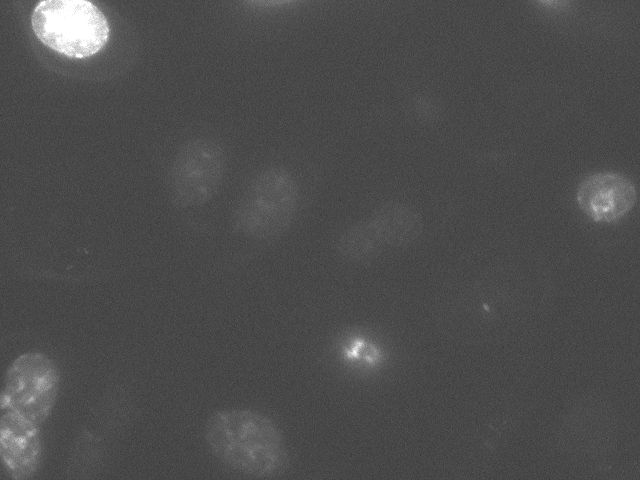

Supplement: Supplementary file 5 — Source Data for Figure 3 [file MSB-19-e11782-s004.zip › Figure 3/3E/Images/20220505_IncenpBiNuFinal_39_76_2.tif]

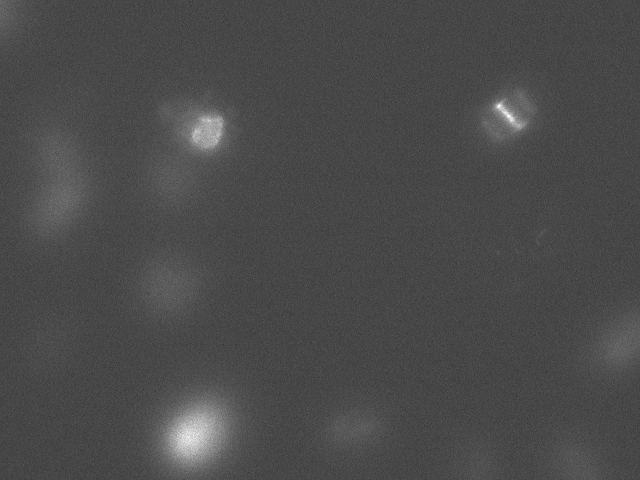

Supplement: Supplementary file 5 — Source Data for Figure 3 [file MSB-19-e11782-s004.zip › Figure 3/3E/Images/20220505_IncenpBiNuFinal_12_23_2.tif]

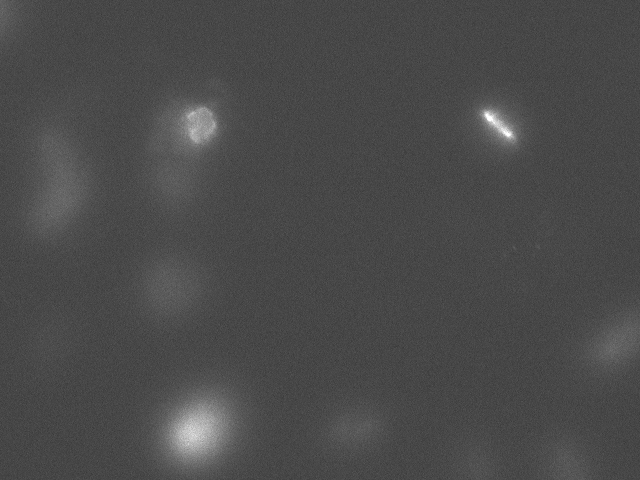

Supplement: Supplementary file 5 — Source Data for Figure 3 [file MSB-19-e11782-s004.zip › Figure 3/3E/Images/20220505_IncenpBiNuFinal_12_21_2.tif]

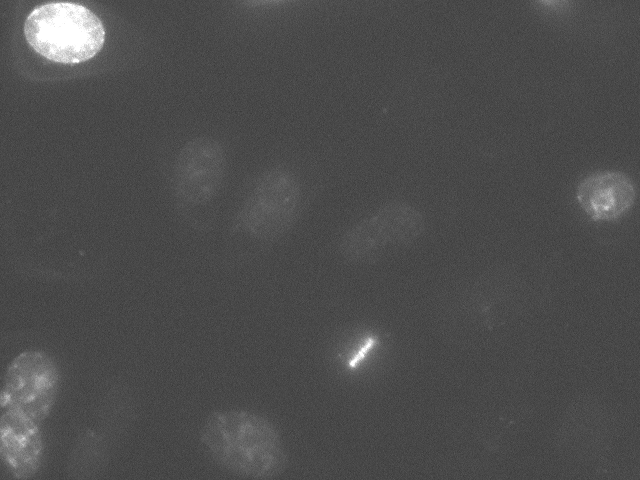

Supplement: Supplementary file 5 — Source Data for Figure 3 [file MSB-19-e11782-s004.zip › Figure 3/3E/Images/20220505_IncenpBiNuFinal_39_74_2.tif]

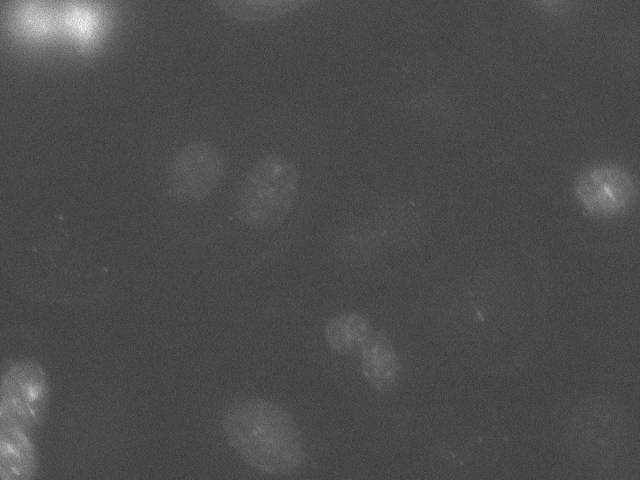

Supplement: Supplementary file 5 — Source Data for Figure 3 [file MSB-19-e11782-s004.zip › Figure 3/3E/Images/20220505_IncenpBiNuFinal_39_90_2.tif]

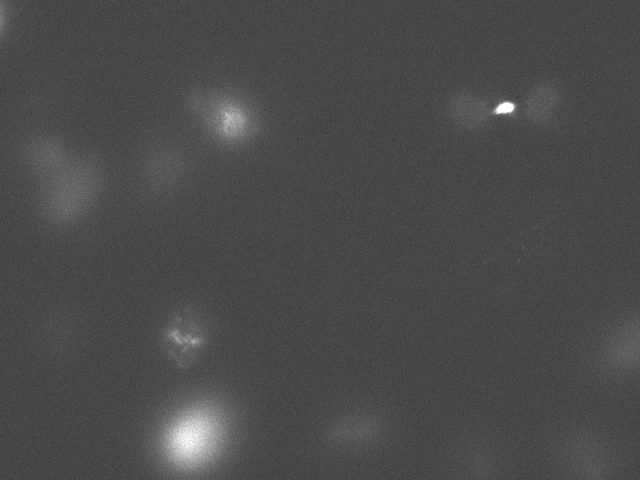

Supplement: Supplementary file 5 — Source Data for Figure 3 [file MSB-19-e11782-s004.zip › Figure 3/3E/Images/20220505_IncenpBiNuFinal_12_27_2.tif]

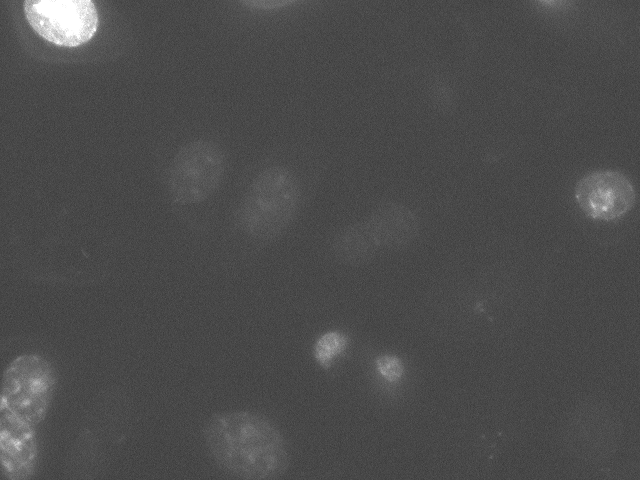

Supplement: Supplementary file 5 — Source Data for Figure 3 [file MSB-19-e11782-s004.zip › Figure 3/3E/Images/20220505_IncenpBiNuFinal_39_80_2.tif]

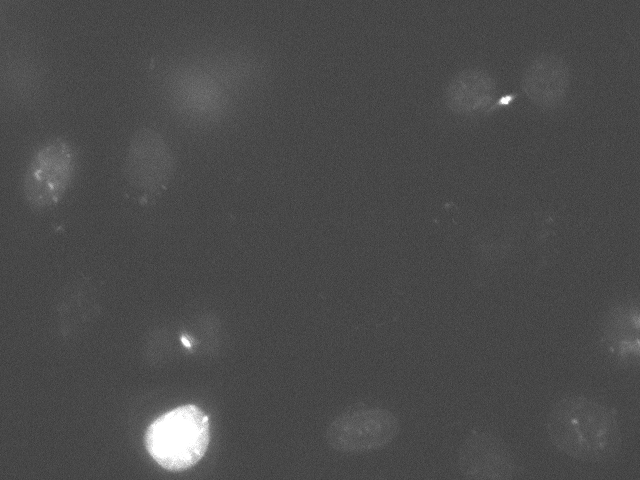

Supplement: Supplementary file 5 — Source Data for Figure 3 [file MSB-19-e11782-s004.zip › Figure 3/3E/Images/20220505_IncenpBiNuFinal_12_37_2.tif]

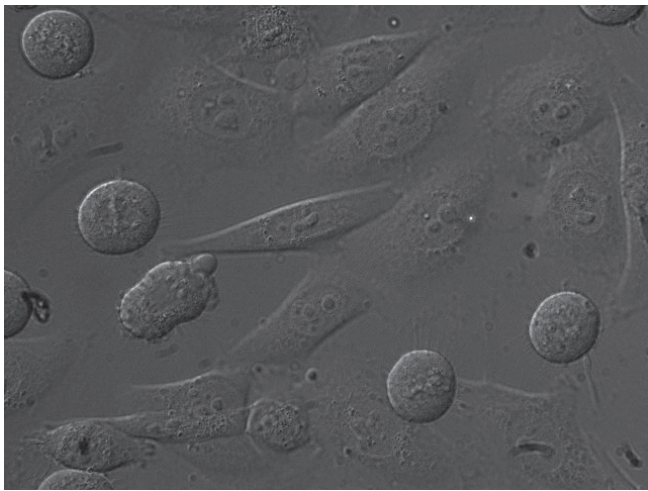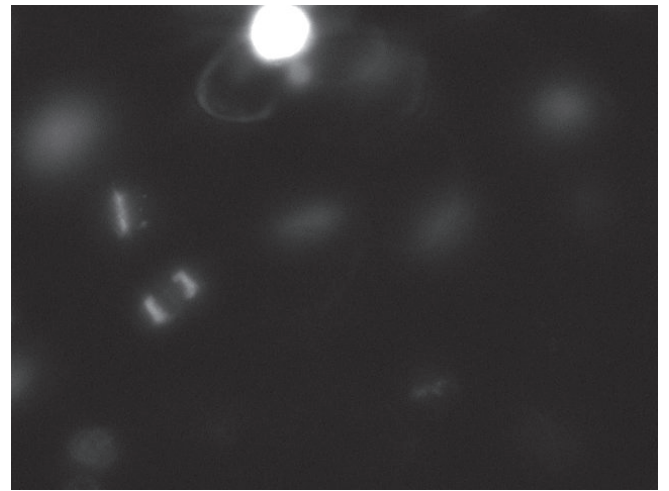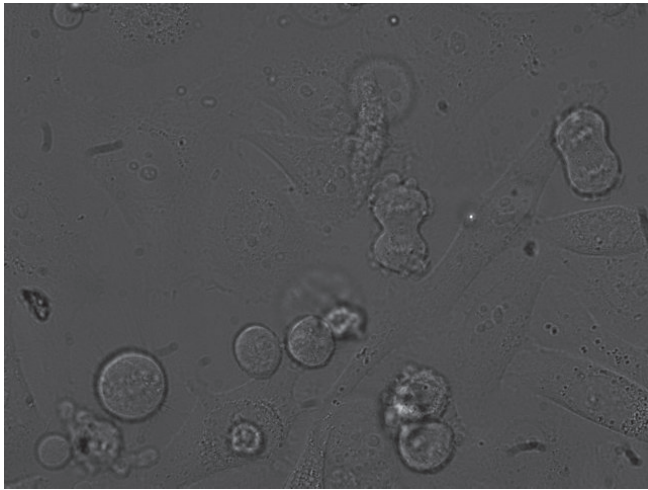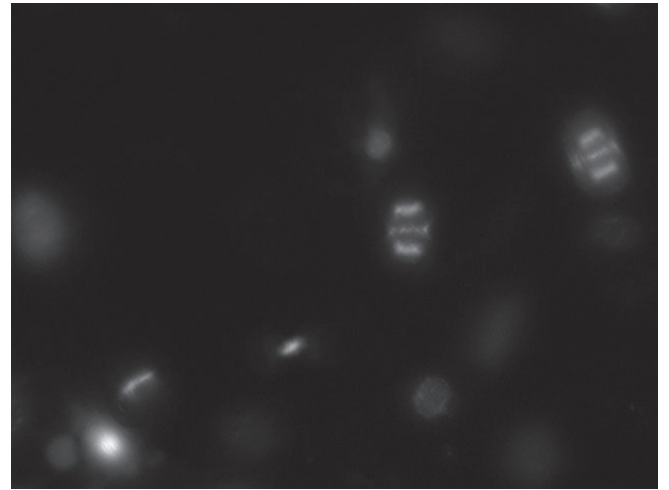

Supplement: Supplementary file 5 — Source Data for Figure 3 [file MSB-19-e11782-s004.zip › Figure 3/3D/Images/Untitled-3.pdf]
