# Supplementary material for: Phosphatase specificity principles uncovered by MRBLE:Dephos and global substrate identification
Source: Mol Syst Biol. 2023 Nov 2;19(12):e11782. doi: 10.15252/msb.202311782 (PMC10698503; doi:10.15252/msb.202311782)
Supplement: Supplementary file 7 [file MSB-19-e11782-s003.pdf]

molecular systems biology

Phosphatase specificity principles uncovered by MRBLE: Dephos and global substrate identification

Jamin Hein, Hieu Nguyen, Dimitriya Garvanska, Isha Nasa, Thomas Kruse, Yinnian Feng, Blanca Lopez-Mendez, Norman Davey, Arminja Kettenbach, Polly Fordyce, and Jakob Nilsson
DOI: 10.15252/msb.202311782

Corresponding author(s): Jakob Nilsson (jakob.nilsson@cpr.ku.dk) , Arminja Kettenbach (arminja.n.kettenbach@dartmouth.edu), Polly Fordyce (pfordyce@stanford.edu)

Review Timeline:

Submission Date:	21st May 23
Editorial Decision:	19th Jun 23
Revision Received:	31st Aug 23
Editorial Decision:	9th Oct 23
Revision Received:	14th Oct 23
Accepted:	16th Oct 23

Editor: Maria Polychronidou

Transaction Report:

(Note: With the exception of the correction of typographical or spelling errors that could be a source of ambiguity, letters and reports are not edited. Depending on transfer agreements, referee reports obtained elsewhere may or may not be included in this compilation. Referee reports are anonymous unless the Referee chooses to sign their reports.)

19th Jun 2023

Manuscript Number: MSB-2023-11782
Title: Phosphatase specificity principles uncovered by MRBLE-Dephos and global substrate identification

Dear Jakob,

Thank you again for submitting your work to Molecular Systems Biology. We have now heard back from the three reviewers who agreed to evaluate your study. As you will see below, the reviewers think that the study is a relevant contribution to the phosphatase field. They raise however a series of concerns, which we would ask you to address in a revision.

I think that the reviewers' recommendations are rather clear so I do not see the need to repeat any of the points listed below. All issues raised by the reviewers need to be satisfactorily addressed. As you may already know, our editorial policy allows in principle a single round of major revision, so it is essential to provide responses to the reviewers' comments that are as complete as possible. I understand that the required revisions are substantive. Please feel free to contact me in case you would like to discuss in further detail any of the issues raised or if you would like to share your revision plan with me. I would be happy to schedule a call.

On a more editorial level, we would ask you to address the following points:

- Please provide a .doc version of the manuscript text (including legends for the main figures) and individual production quality figure files for the main Figures (one file per figure).

- Please include 5 keywords.

- We have replaced Supplementary Information by the Expanded View (EV format). If additional figures are provided during revision they can be included in a PDF called Appendix. Appendix figures should be labeled and called out as: "Appendix Figure S1, Appendix Figure S2... Appendix Table S1..." etc. Each legend should be below the corresponding Figure/Table in the Appendix. Please include a Table of Contents in the beginning of the Appendix. For detailed instructions regarding expanded view please refer to our Author Guidelines: .

- Supplementary Tables 1-2 should be provided as Datasets EV1-EV2. Please provide one file per dataset and include a description of the dataset in the .xls file as a separate tab.

- Please provide a "standfirst text" summarizing the study in one or two sentences (approximately 250 characters), three to four "bullet points" highlighting the main findings and a "synopsis image" (550px width and max 400px height, jpeg format) to highlight the paper on our homepage.

- All Materials and Methods need to be described in the main text. We would encourage you to use 'Structured Methods', our new Materials and Methods format. According to this format, the Material and Methods section should include a Reagents and Tools Table (listing key reagents, experimental models, software and relevant equipment and including their sources and relevant identifiers) followed by a Methods and Protocols section in which we encourage the authors to describe their methods using a step-by-step protocol format with bullet points, to facilitate the adoption of the methodologies across labs. More information on how to adhere to this format as well as downloadable templates (.doc or .xls) for the Reagents and Tools Table can be found in our author guidelines: . An example of a Method paper with Structured Methods can be found here:

- Please include a "Disclosure and Competing Interests Statement" in the main text.

- Please include a Data availability section describing how the data, code etc. have been made available. This section needs to be formatted according to the example below:
The datasets and computer code produced in this study are available in the following databases:
- Chip-Seq data: Gene Expression Omnibus GSE46748 (https://www.ncbi.nlm.nih.gov/geo/query/acc.cgi?acc=GSE46748)
- Modeling computer scripts: GitHub (https://github.com/SysBioChalmers/GECKO/releases/tag/v1.0)
- [data type]: [full name of the resource] [accession number/identifier] ([doi or URL or identifiers.org/DATABASE:ACCESSION])

- The References should be formatted according to the Molecular Systems Biology reference style (i.e., ordered alphabetically and listing the first 10 authors followed by et al.)

- For data quantification: please specify the name of the statistical test used to generate error bars and P values, the number (n) of independent experiments (specify technical or biological replicates) underlying each data point and the test used to calculate p-values in each figure legend. The figure legends should contain a basic description of n, P and the test applied. Graphs must

include a description of the bars and the error bars (s.d., s.e.m.).

- When you resubmit your manuscript, please download our CHECKLIST (https://bit.ly/EMBOPressAuthorChecklist) and include the completed form in your submission.
Please note that the Author Checklist will be published alongside the paper as part of the transparent process (https://www.embopress.org/page/journal/17444292/authorguide#transparentprocess).

If you feel you can satisfactorily deal with these points and those listed by the referees, you may wish to submit a revised version of your manuscript. Please attach a covering letter giving details of the way in which you have handled each of the points raised by the referees. A revised manuscript will be once again subject to review and you probably understand that we can give you no guarantee at this stage that the eventual outcome will be favorable.

Kind regards,

Maria

Maria Polychronidou, PhD
Senior Editor
Molecular Systems Biology

We realize that it is difficult to revise to a specific deadline. In the interest of protecting the conceptual advance provided by the work, we recommend a revision within 3 months (17th Sep 2023). Please discuss the revision progress ahead of this time with the editor if you require more time to complete the revisions. Use the link below to submit your revision:

https://msb.msubmit.net/cgi-bin/main.plex

IMPORTANT: When you send your revision, we will require the following items:
1. the manuscript text in LaTeX, RTF or MS Word format
2. a letter with a detailed description of the changes made in response to the referees. Please specify clearly the exact places in the text (pages and paragraphs) where each change has been made in response to each specific comment given
3. three to four 'bullet points' highlighting the main findings of your study
4. a short 'blurb' text summarizing in two sentences the study (max. 250 characters)
5. a 'thumbnail image' (550px width and max 400px height, Illustrator, PowerPoint or jpeg format), which can be used as 'visual title' for the synopsis section of your paper.
6. Please include an author contributions statement after the Acknowledgements section (see https://www.embopress.org/page/journal/17444292/authorguide)
7. Please complete the CHECKLIST available at (https://bit.ly/EMBOPressAuthorChecklist).
Please note that the Author Checklist will be published alongside the paper as part of the transparent process (https://www.embopress.org/page/journal/17444292/authorguide#transparentprocess).
8. When assembling figures, please refer to our figure preparation guideline in order to ensure proper formatting and readability in print as well as on screen:
https://bit.ly/EMBOPressFigurePreparationGuideline
See also figure legend guidelines: https://www.embopress.org/page/journal/17444292/authorguide#figureformat
9. Please note that corresponding authors are required to supply an ORCID ID for their name upon submission of a revised manuscript (EMBO Press signed a joint statement to encourage ORCID adoption).
(https://www.embopress.org/page/journal/17444292/authorguide#editorialprocess)
Currently, our records indicate that the ORCID for your account is 0000-0003-4100-1125.

Please click the link below to modify this ORCID:
Link Not Available

10. At EMBO Press we ask authors to provide source data for the main manuscript figures. Our source data coordinator will contact you to discuss which figure panels we would need source data for and will also provide you with helpful tips on how to upload and organize the files.

The system will prompt you to fill in your funding and payment information. This will allow Wiley to send you a quote for the article processing charge (APC) in case of acceptance. This quote takes into account any reduction or fee waivers that you may be eligible for. Authors do not need to pay any fees before their manuscript is accepted and transferred to the publisher.

EMBO Press participates in many Publish and Read agreements that allow authors to publish Open Access with reduced/no publication charges. Check your eligibility: https://authorservices.wiley.com/author-resources/Journal-Authors/open-access/affiliation-policies-payments/index.html

As a matter of course, please make sure that you have correctly followed the instructions for authors as given on the submission website.

*** PLEASE NOTE *** As part of the EMBO Press transparent editorial process initiative (see our Editorial at https://dx.doi.org/10.1038/msb.2010.72), Molecular Systems Biology publishes online a Review Process File with each accepted manuscripts. This file will be published in conjunction with your paper and will include the anonymous referee reports, your point-by-point response and all pertinent correspondence relating to the manuscript. If you do NOT want this File to be published, please inform the editorial office at msb@embo.org within 14 days upon receipt of the present letter.

Reviewer #1:

This manuscript Hein et al. presents a very exciting high-throughput method to characterize the substrate sequence specificities of protein phosphatases: MRBLE-Dephos. This method builds on the MRBLE platform developed by the Fordyce lab with the added detection of phosphopeptides dephosphorylation as a function of time. The method allows for rapid, quantitative comparison of dephosphorylation rates for 94 peptides at a time, and this was applied with a tailored peptide library to analyze pT/pS-proximal sequence preferences for the phosphatases PP1 and PP2A-B55. Juxtaposition of the sequence preferences identified using MRBLE-Dephos with proteomics data from this study and published studies allow for a clear assessment of the extent to which catalytic domain sequence specificity governs substrate recognition in a cellular context. The manuscript ends by examining the contributions of a secondary binding motif (RVxF) in PP1 substrate selection, by juxtaposing interactomics experiments, phosphoproteomics data, and MRBLE binding assays.

Overall, this is a large body of work that makes significant contributions to our understanding of phosphoprotein phosphatase biochemistry and signaling. As currently presented, however, the second half of the manuscript, focused on the RVxF interactions, is hard to follow. In particular, the descriptions of the analysis describing how RVxF motif binding overrides catalytic domain preferences are not clear. Several major and minor revisions (largely non-experimental) are suggested in order to improve the depth of the analysis and also the clarity of the manuscript. Given the scope and significance of this study, both in terms of methods and concepts, a refined version of this manuscript would be a very welcome contribution to the phosphatase signaling field.

Suggested major points to address:
1. Can the authors clarify how many beads they are imaging for each pooled dephosphorylation reaction? Are there multiple beads per peptide, and if so, how is this treated in the data analysis? Related to this, does this depth matter? In other words, if you have many beads in one reaction corresponding to the same peptide, and the rates for these beads are averaged, does this improve the method? This would be distinct from doing multiple separate dephosphorylation reactions (almost like technical vs "biological" replicates).
2. In Figure 1F, it's very difficult to evaluate how well the dephos score correlates with data from the orthogonal assay, as currently plotted. It might be helpful to either plot a scatterplot of the comparison and/or report a correlation coefficient between the datasets. Since the rank-order of substrates via dephos score and phosphate release score don't appear to be identical for the panel tested (although they are close), can the authors discuss the potential sources of these discrepancies?
3. Can the authors explain how they know that Nipp1 and thio-Arpp19 are selective inhibitors of PP1 and PP2A-B55, respectively, and not of other PPPs? Is it possible to do an IP or other experiment that might show if the two inhibitors are binding to other phosphatases in the cell lysates, or perhaps such an experiment has been published previously? This is particularly important, given the very low overlap between the PP1 dataset in this paper and that from references 10 and 11.
4. It is surprising that, in panel 2D, those sequences that are biased toward PP1 over PP2A-B55 appear to have a +1 proline, but that a +1 Pro is a preferred residue for PP2A-B55 and a disfavored residue for PP1 in the proteomics experiments (Figure 2E). Can the authors explain/discuss this discrepancy more? It is already suggested in the text that this might be due to prolyl isomerization, or do other cellular interactors driving co-localization and overriding the anti-proline preference observed in vitro. Another possibility is that the favoring of a proline at the +1 position is dependent on some other sequence feature that is not present in the MRBLE library but is present in the subset of the proteome that is enriched. This should be examined. Yet another possibility is that PP2A-B55 is not being selectively inhibited in the proteomics experiment, and the observed +1 P signal is coming from another phosphatase. Finally, as hinted at by the authors, it may simply be the case that the cellular substrates of PP2A-B55 are not enzymatically optimal, because that might not be what is optimal for cell signaling (i.e. a faster dephosphorylation reaction might not be ideal for what the cell needs in order to function properly). Thus, other recognition features of the substrates are sufficient to select the substrates, and the +1 P keeps the rate of dephosphorylation low. Overall, these points need further discussion.
5. Given the issues posed in the previous point, it would be worthwhile to examine INCENP phosphorylation by PP2A-B55 at the peptide level with a mutation at P60 (perhaps P60A). Cellular assays with this mutant would also be interesting.
6. For Figure 3C, the S61E mutation is tested in cells, and it is suggested that Cdk1 can still phosphorylate this mutant. The figure legend indicates that the data shown are one representative dataset from three replicates. For the quantification, data from all three replicates, along with error bars, should be shown.

7. For all of the proteomics analysis, the authors should include a full description of the statistical analysis used to identify significant phosphosites or interactors (specifying number of replicates, fold-change cutoffs, and p-value cutoffs). Related to this, it would be helpful to see volcano plots of all of the data, where relevant. And the full volcano plots corresponding to the data in figure 4A,B should be shown in the SI.
8. For the analysis in Figure 4D, the authors are asserting that phosphosites in Ki67 near the RVxF motif more frequently have the SP motif than the TP motif, whether regulated by PP1 or not. By contrast, sites away from Ki67 that are PP1-regulated more frequently have a TP motif instead of SP motif. What is unclear from this analysis, and from the figure, is whether this bias actually reflects an effect of RVxF-mediated localization vs. intrinsic catalytic domain preferences, as suggested, or if this can be explained by the frequency of TP vs SP phosphosites in the protein. What might help make this distinction is to know how many sequences were used to make up each logo.
9. Related to the previous point, a similar analysis could be done for the data in Figures S7 and S8 - The sequence preferences for PP1- and PP2A-regulated sites across the proteome (with and without an associated RVxF site) should be normalized to the all phosphosites on RVxF-associated vs not-associated sites. This would really tell illustrate whether presence of the RVxF motif impacts the substrate sequence tolerance of PP1- and PP2A. In other words, is the dephosphorylation of pSer residues by PP1 actually due to the presence of an RVxF motif nearby, or is it the case that phosphorylation sites near RVxF tend to be pSer sites rather than pThr sites? Same goes for net charge around the phosphosite. What is the distribution of net charge around phosphosites near RVxF vs away from RVxF sites?
10. Were any peptides in the RVxF MRBLE experiment also characterized by ITC or any other classical titration binding experiment? It would be helpful to see how well the Kd values from two orthogonal methods correlate.
11. It is unclear

Suggested minor revisions:
1. Very minor point: In the intro, paragraph 2, it might be useful to clarify whether PP1 and PP2A are the primary PPPs in just eukaryotic cells, or also organismal clades. The current text just says that they are responsible for the majority of Ser/Thr dephosphorylation in cells.
2. For the sequences in Figure 1D, it appears that the phospho-Ser and phospho-Thr residues are labeled as Sp or Tp, whereas these phospho-residues are most conventionally written as pS/pT, which makes reading the sequences a bit confusing.
3. It would be helpful if the logos in figure 2E were in the same color scheme as the logos in the rest of Figure 2.
4. Similar to the previous point, the coloring of the sequences in Figure 2D is the opposite of what is in the logos: in the logos (as is often conventional), D/E are red, and K/R are blue, but in the listed sequences the opposite color scheme is used.
5. This may be a file processing issue, but Figure S7 is too blurry to read the labels on the nodes in the network diagram.
6. In general, it is unclear what the reader stands to learn from the two interaction network diagrams. Related to this, it is quite difficult to discern where PP1 and PP2A-B55 are in these maps. Perhaps the authors could highlight/explain parts of the interaction maps that would be of significance/value to the reader?
7. For non-specialists, it may be helpful to define INCENP and explain its role in mitosis.
8. Minor question: is "dephosphorylome" a common term? If not, while this is a purely stylistic consideration, it may by simpler for the readers if some more common phrasing is used.
9. In the last sentence of the first paragraph, there is a typo: "phosphoyraltion"

Reviewer #2:

In this study, Hein and co-workers address the complex topic of cellular protein phosphatase selectivity. To do this they use mass spectrometry approaches to analyse dephosphorylation of peptide libraries in vitro by PP1 and PP2A-B55, coupled with global identification of cellular substrates for these enzymes as an in vivo correlate. In summary, there is a considerable amount of high-quality work in the study, and the new data will provide a valuable additional resource for the field.

Specific points:

In Figure 3, INCENP is taken as an example to test a key feature of the dephosphorylation motif. Previous work has shown that INCENP relocalisation from pericentromeric chromatin to the anaphase spindle requires dephosphorylation at T59, and can be prevented with a T59E mutant. This forms a switch controlling binding to a motor protein KIF20A at the onset of anaphase. The charge introduced in the S61E mutant may directly prevent binding to KIF20A. Additional controls are necessary for Figure 3D-E, where a T59A/S61E mutant should be tested. If the authors are correct, then this should to the anaphase spindle since it cannot be phosphorylated. If it fails to localise then defective anaphase localisation is due to a blockade of KIF20A binding and not a dephosphorylation defect.

The authors note that while the overlap between PP2A-B55 substrates in vivo in their data and previous work is good, the overlap for PP1 is less clear. There are good reasons for this which need a little more explanation. This also highlights some caveats to the peptide approach, which is important since PP1 is generally thought to act in complex with other regulatory subunits that modify substrate selection. Using PP1 alone in the in vitro approach, and taking Nipp1 as the sole method to modulate PP1 in vivo may not address the full range of PP1 substrates. PP2A-B55 is probably only regulated by Arpp19/ENSA, so the approach would be more general in terms of the range of substrates.

Figure 4 is confusing and it is difficult to extract a clear message. Is there some other way to represent the choice of network diagram is not obvious to me, since the study isn't focussed on the interactions between proteins, but rather the sequences of individual phosphatase sites. Is there some way to strengthen the link to Figure 5 in terms of testing whether dephosphorylation alters behaviour of a key PP1 regulated protein or proteins. In that sense the data in Figure S10 is important and could be moved to a main figure.

Reviewer #3:

This manuscript describes an adaptation of the MRBLE peptide:protein interaction assay to a format (MRLBE-Dephos) that can be used to measure time-dependent peptide dephosphorylation by phosphatases. The authors have subsequently used this assay to compare the substrate selectivity of two key phosphatases (PP1 and PP2A-B55), either purified or as present in mitotic cell lysates. The MRLBE-Dephos assay is novel and generated interesting data, but needs to be better validated. Also, while the observed determinants of substrate selectivity of PP1 and PP2A-B55 are largely in agreement with previously established determinants, as derived from other experimental approaches, there are obvious differences that need to be further explored. My detailed comments are detailed below.

Specific comments

1. What are the concentrations of the peptides and phosphatases during the MRLBR-dephos assay? The method section only specifies volumes or amounts. This is essential information needed for for comparison with other phosphatase assays.

2. The MRLBE-Dephos assay measures remaining phosphate (high signal less high signal), which is less sensitive than classical assays that quantify phosphate release (no signal   signal). This should be commented upon.

3. In all MRLBE dephosphorylation assays (Fig. S1), the dephosphorylation apparently stops before completion. The reason for this is not explained. Also, since the dephosphorylation is not linear with time (Fig. S1), it is not clear how the dephosphorylation score can be accurately derived from the first and last time points.

4. A comparison of classical phosphatase assays (phosphate release) and the MRLBE-Dephos score (Fig. 1E) shows essentially no dephosphorylation for some peptides with the first assay but still very significant dephosphorylation with the second assay. This indicates that the MRLBE-Dephos assay not only measures phosphatase activity but, e.g., also loss of bead-associated peptide.

5. Since the peptides were synthesized on the beads, it should be explored whether binding to the beads affects their properties as substrates.

6. It is biologically not relevant to compare the substrate selectivity of the PP1 monomer, which does not exist in vivo, with a PP2A-B55 holoenzyme. Also, bacterially expressed PP1 has a substrate selectivity that is very different from that of native PP1 (https://doi.org/10.1111/j.1432-1033.1993), making a direct comparison even more irrelevant.

7. The most striking feature of the comparison between purified PP1 and PP2A-B55 is their similar activity on a broad range of peptide substrates (Fig. 1D; Fig. 2A-D). It is difficult to grasp how the observed minor differences can explain the clearly distinct, albeit somewhat overlapping functions, of these phosphatases in vivo. The differences in substrate specificity would most likely have been much more obvious if a specific PP1 holoenzyme and PP2A holoenzyme were compared, with the free catalytic subunits as reference points (see previous comment).

8. It is not correct to refer to 'benchmark in vitro data with in vivo substrates' (p8, line 1) when the actual comparison is purified phosphatases versus phosphatases present in mitotic lysates.

9. While thio-phosphorylated proteins are somewhat resistant to dephosphorylation, they will eventually also be dephosphorylated. Hence, the authors should examine to which extent thiophosphorylated Arpp19 is dephosphorylated in the adopted timeframe(s). Nipp1 is indeed a highly selective inhibitor of PP1 but it only prevents the dephosphorylation of a subset of substrates. This limitation should be indicated.

10. Less than 10% of the identified mitotic phosphorylation sites (˃ 38,000) were affected by PP1 or PP2A-B55 inhibition. This indicates that the inhibition of these phosphatases was incomplete and/or that it is incorrect to that these phosphatases represent the major mitotic-exit phosphatases.

11. p9, first line: H3S29 does not exist. Did the authors aim to refer to H3S28?

12. The limited overlap between PP1 substrates in mitotic lysates and previous substrate-mapping studies is worrysome, and is

further evidence that PP1 was only partially blocked in the mitotic lysates.

13. The identified PP1 and B55 interactors (Table S2) are poorly described and not independently validated. Are they regulatory subunits and/or substrates? How much overlap is there with known interactors?

14. The interaction of PP1 with RVxF motifs and its regulation by mitotic phosphorylation of residues within or next to the RVxF motif has already been studied in some detail by various research groups and is not properly referred to in the manuscript.

Reviewer #1:

This manuscript Hein et al. presents a very exciting high-throughput method to characterize the substrate sequence specificities of protein phosphatases: MRBLE-Dephos. This method builds on the MRBLE platform developed by the Fordyce lab with the added detection of phosphopeptides dephosphorylation as a function of time. The method allows for rapid, quantitative comparison of dephosphorylation rates for 94 peptides at a time, and this was applied with a tailored peptide library to analyze pT/pS-proximal sequence preferences for the phosphatases PP1 and PP2A-B55. Juxtaposition of the sequence preferences identified using MRBLE-Dephos with proteomics data from this study and published studies allow for a clear assessment of the extent to which catalytic domain sequence specificity governs substrate recognition in a cellular context. The manuscript ends by examining the contributions of a secondary binding motif (RVxF) in PP1 substrate selection, by juxtaposing interactomics experiments, phosphoproteomics data, and MRBLE binding assays.

Overall, this is a large body of work that makes significant contributions to our understanding of phosphoprotein phosphatase biochemistry and signaling. As currently presented, however, the second half of the manuscript, focused on the RVxF interactions, is hard to follow. In particular, the descriptions of the analysis describing how RVxF motif binding overrides catalytic domain preferences are not clear. Several major and minor revisions (largely non-experimental) are suggested in order to improve the depth of the analysis and also the clarity of the manuscript. Given the scope and significance of this study, both in terms of methods and concepts, a refined version of this manuscript would be a very welcome contribution to the phosphatase signaling field.

Suggested major points to address:

1. Can the authors clarify how many beads they are imaging for each pooled dephosphorylation reaction? Are there multiple beads per peptide, and if so, how is this treated in the data analysis? Related to this, does this depth matter? In other words, if you have many beads in one reaction corresponding to the same peptide, and the rates for these beads are averaged, does this improve the method? This would be distinct from doing multiple separate dephosphorylation reactions (almost like technical vs "biological" replicates).

We appreciate the reviewer's careful reading of the manuscript and apologize that the description of how we perform experiments was not sufficiently clear. In a typical MRBLE:Dephos experiment, we aim to profile ~10,000 beads (corresponding to an average of ~100 beads per code). As suspected by the Reviewer, individual bead measurements can be somewhat noisy and averaging over intensities across many beads increases our experimental accuracy.

To increase clarity, we now include an SI figure showing the number of beads profiled per code in each experiment (Fig. EV1), which we also reproduce here (Fig. R1).

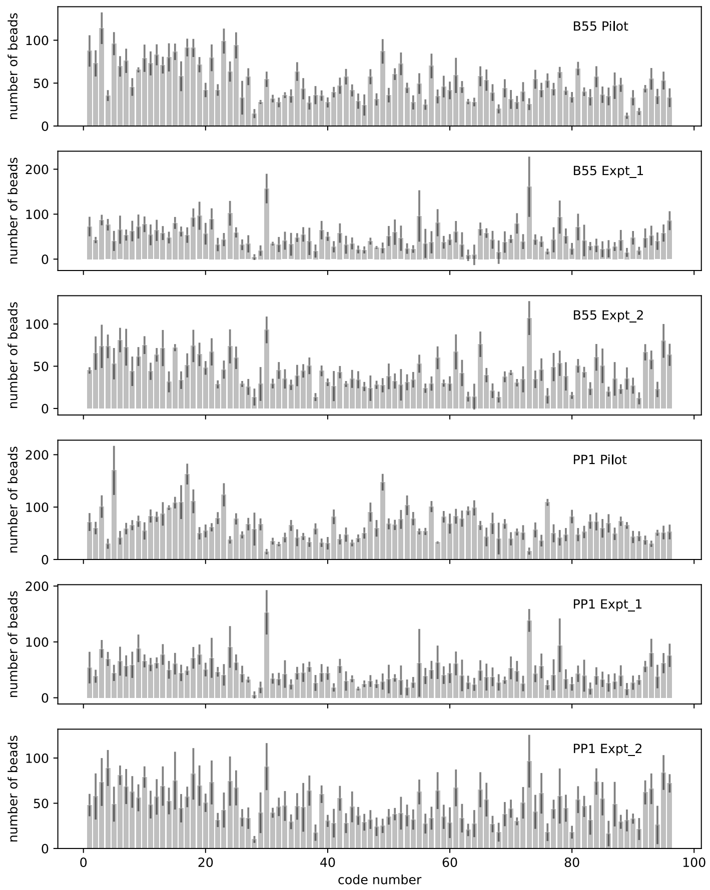

Fig. R1. Number of beads per code per experiment. *Bars and error bars represent the median and standard deviation of the number of beads profiled across all timepoints in each experiment.*

2. In Figure 1F, it's very difficult to evaluate how well the dephos score correlates with data from the orthogonal assay, as currently plotted. It might be helpful to either plot a scatterplot of the comparison and/or report a correlation coefficient between the datasets. Since the rank-order of substrates via dephos score and phosphate

release score don't appear to be identical for the panel tested (although they are close), can the authors discuss the potential sources of these discrepancies?

In response to the Reviewer's request, we now include a scatter plot directly comparing the MRBLE:dephos dephosphorylation score with the amount of phosphate released in orthogonal assays along with a linear regression to help visualize correlation. This plot is now included as Fig. 1F (reproduced here as Fig. R2 for convenience).

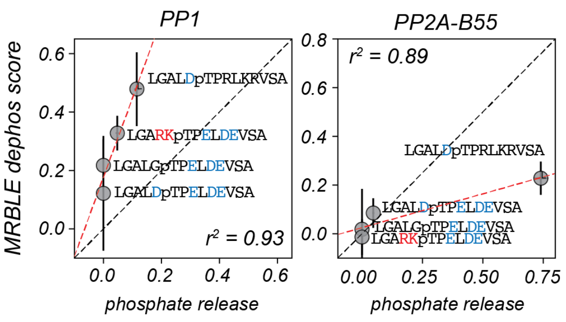

Fig. R2. Correlation between results of MRBLE:Dephos and traditional phosphate release experiments. *Markers and error bars indicate mean and standard deviation of dephos scores across experiments, black dashed line indicates the identity line, and red dashed line indicates a linear regression.*

3. Can the authors explain how they know that Nipp1 and thio-Arpp19 are selective inhibitors of PP1 and PP2A-B55, respectively, and not of other PPPs? Is it possible to do an IP or other experiment that might show if the two inhibitors are binding to other phosphatases in the cell lysates, or perhaps such an experiment has been published previously? This is particularly important, given the very low overlap between the PP1 dataset in this paper and that from references 10 and 11.

We appreciate these comments from the reviewer. Both Nipp1 and thio-Arpp19 has been extensively characterized as specific inhibitors of PP1 and PP2A-B55 by other labs. Nipp1 binds PP1 through and RVxF motif while thio-Arpp19 blocks the active site of PP2A-B55. The previous work characterizing Nipp1 and Arpp19 are cited in the manuscript. In the revised manuscript we write on page 9:

"We used the central domain of Nipp1 and thiophosphorylated Arpp19 (thio-Arpp19) as specific natural inhibitors of PP1 and PP2A-B55, respectively [23-25]. Nipp1 prevents binding of RVxF-containing proteins to PP1, in this way preventing a large subset of PP1 holoenzymes from forming and thus blocking PP1 dephosphorylation. In contrast, thio-Arpp19 blocks the active site of PP2A-B55, effectively blocking activity."

4. It is surprising that, in panel 2D, those sequences that are biased toward PP1 over PP2A-B55 appear to have a +1 proline, but that a +1 Pro is a preferred residue for PP2A-B55 and a disfavored residue for PP1 in the proteomics experiments (Figure 2E). Can the authors explain/discuss this discrepancy more? It is already suggested in the text that this might be due to prolyl isomerization, or do other cellular interactors driving co-localization and overriding the anti-proline preference observed in vitro. Another possibility is that the favoring of a proline at the +1 position is dependent on some other sequence feature that is not present in the MRBLE library but is present in the subset of the proteome that is enriched. This should be examined. Yet another possibility is that PP2A-B55 is not being selectively inhibited in the proteomics experiment, and the observed +1 P signal is coming from another phosphatase. Finally, as hinted at by the authors, it may simply be the case that the cellular substrates of PP2A-B55 are not enzymatically optimal, because that might not be what is optimal for cell signaling (i.e. a faster dephosphorylation reaction might not be ideal for what the cell needs in order to function properly). Thus, other recognition features of the substrates are sufficient to select the substrates, and the +1 P keeps the rate of dephosphorylation low. Overall, these points need further discussion.

The reviewer is correct. Dephosphorylation of proline-directed sites by PP1 depends on additional sequence features.

Consistently, the preference of PP1 for proline-directed sequence in both assays appears to be specific for TP. The TP sites favored by PP1 over B55 frequently have basic residues before and acidic residues after the phosphorylated TP (Figure R3 - panel A and B below). Indeed, the residues are deselected by B55 Figure 2E. The differences in the position of these amino acids relative to the TP (eg E in+2 proteomics, and DE in +4 and +5 MRBLE) might steam from the composition of the peptide library (Figure R3 – panel C).

The dephosphorylation preference of these TP sites by PP1 is not obvious in Figure 2E due to the large number of proline-directed phosphorylation sites in mitosis. To determine the enrichment of sequence elements, background correction is used for icelogos. Here, the background consisted of the non-regulated sites from our phosphoprotemics (Figure 2E). While the lack of dephosphorylation of SP sites by PP1 is

obvious, the dephosphorylation of specific "charged" TP sites does occur but not at a higher rate than the overall pool of TP phosphorylation sites.

We have discussed some of the aspects in the revised manuscript on page 10.

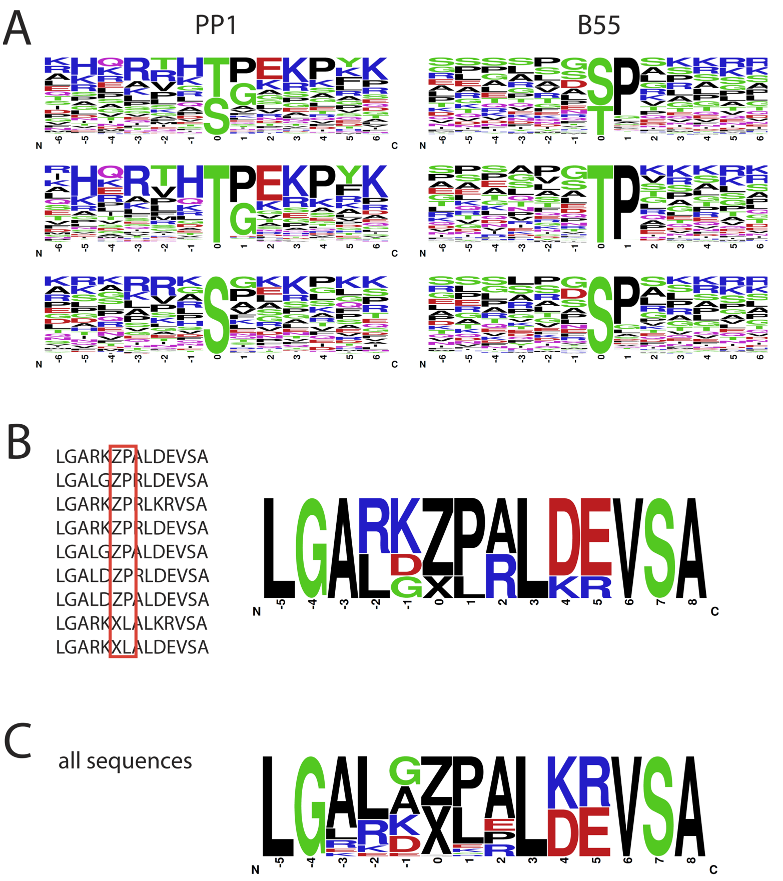

Figure R3. A. Weblogos of significantly increased phosphorylation upon Nipp1 (PP1) and thio-Arpp19 (PP2A-B55) addition. Top row: all sites, middle row: threonine sites, bottom row: serine sites. B. Weblogo of MRBLE peptides preferred by PP1 versus PP2A-B55 (reproduced from Figure 2D). C. Weblogo of all MRBLE peptides tested.

5. Given the issues posed in the previous point, it would be worthwhile to examine INCENP phosphorylation by PP2A-B55 at the peptide level with a mutation at P60 (perhaps P60A). Cellular assays with this mutant would also be interesting.

We have performed this experiment in vitro and as expected the P60A peptide is dephosphorylated more efficiently by PP2A-B55 consistent with MRBLE-Dephos and previous work showing that a Pro residue in +1 is inhibitory (Figure R4). We have not included this analysis in the revised manuscript. We have not performed cellular assays with the INCENP P60A mutant as this mutation would prevent Cdk1 phosphorylation.

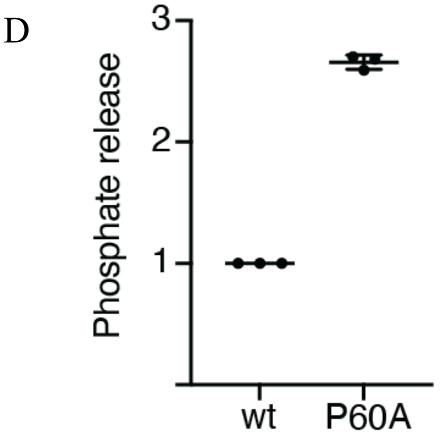

Figure R4. In vitro dephosphorylation assay with PP2A-B55 and either WT INCENP peptide or P60A

6. For Figure 3C, the S61E mutation is tested in cells, and it is suggested that Cdk1 can still phosphorylate this mutant. The figure legend indicates that the data shown are one representative dataset from three replicates. For the quantification, data from all three replicates, along with error bars, should be shown.

We have updated the figure and shown below for convenience.

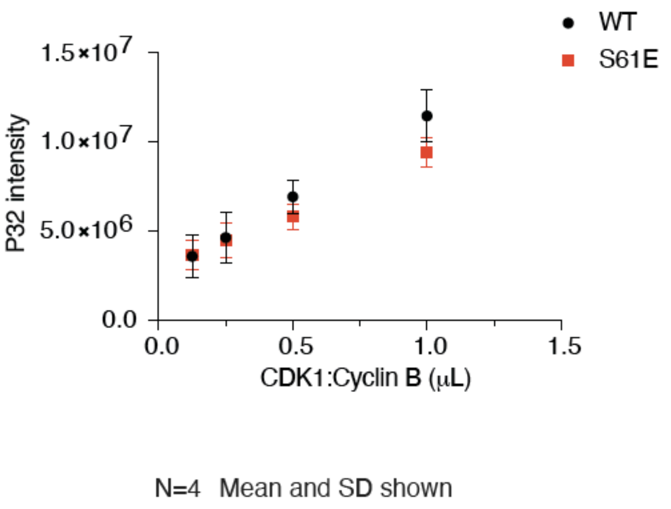

Figure R5. In vitro phosphorylation of INCENP WT or S61E. Mean with SD n=4.

7. For all of the proteomics analysis, the authors should include a full description of the statistical analysis used to identify significant phosphosites or interactors (specifying number of replicates, fold-change cutoffs, and p-value cutoffs). Related to this, it would be helpful to see volcano plots of all of the data, where relevant. And the full volcano plots corresponding to the data in figure 4A,B should be shown in the SI.

We have provided this information and provided volcano plots of Fig. 4A and B in the main figure 4 of the revised manuscript. Shown below for convenience.

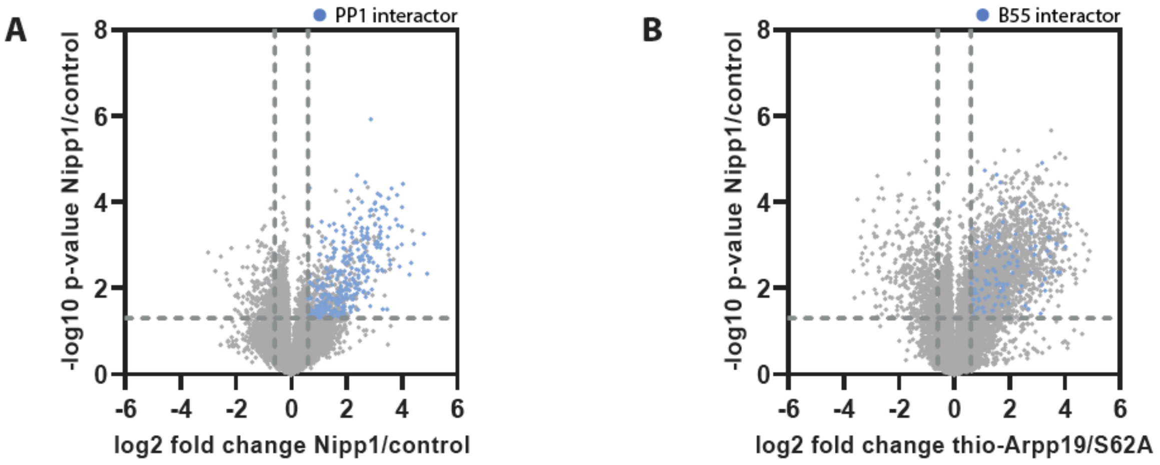

Figure R6. Volcano plots showing regulated sites and interactors.

8. For the analysis in Figure 4D, the authors are asserting that phosphosites in Ki67 near the RVxF motif more frequently have the SP motif than the TP motif, whether regulated by PP1 or not. By contrast, sites away from Ki67 that are PP1-regulated more frequently have a TP motif instead of SP motif. What is unclear from this analysis, and from the figure, is whether this bias actually reflects an effect of RVxF-mediated localization vs. intrinsic catalytic domain preferences, as suggested, or if this can be explained by the frequency of TP vs SP phosphosites in the protein. What might help make this distinction is to know how many sequences were used to make up each logo.

This is a relevant point raised by the reviewer. We have provided these numbers in the revised manuscript (in figure legend to Fig. 4) and also below.

Regulated sites 1-1000: 17 localized single phosphorylation sites (12 S, 5 T of which 2 SP, 5 TP)
Regulated sites 1001-end: 51 localized single phosphorylation sites (15 S, 36 T of which 0 SP, 33 TP)

Not regulated sites 1-1000: 7 localized single phosphorylation sites (5 S, 2 T of which 4 SP, 1 TP)
Not regulated sites 1001-end: 42 localized single phosphorylation sites (31 S, 11 T of which 16 SP, 6 TP)

9. Related to the previous point, a similar analysis could be done for the data in Figures S7 and S8 - The sequence preferences for PP1- and PP2A-regulated sites across the proteome (with and without an associated RVxF site) should be normalized to the all phosphosites on RVxF-associated vs not-associated sites. This would really tell illustrate whether presence of the RVxF motif impacts the substrate sequence tolerance of PP1- and PP2A. In other words, is the dephosphorylation of pSer residues by PP1 actually due to the presence of an RVxF motif nearby, or is it the case that phosphorylation sites near RVxF tend to be pSer sites rather than pThr sites? Same goes for net charge around the phosphosite. What is the distribution of net charge around phosphosites near RVxF vs away from RVxF sites?

Looking at proteins beyond KI67, we did not detect a difference in pSer versus pThr sites presence on proteins with RVxF motifs compared to sites on proteins without RVxF motifs that are PP1 substrates through indirect recruitment.

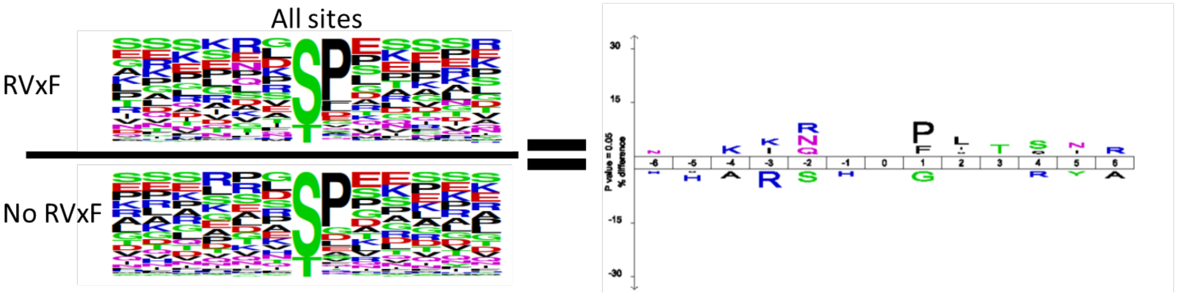

Figure R7.

However, as observed for KI67, pSer sites on proteins away from RVxF or on proteins without RVxF sites that are indirectly recruited to PP1 are less likely to be dephosphorylated compared to pThr, while there was no difference for pSer sites on RVxF-containing proteins.

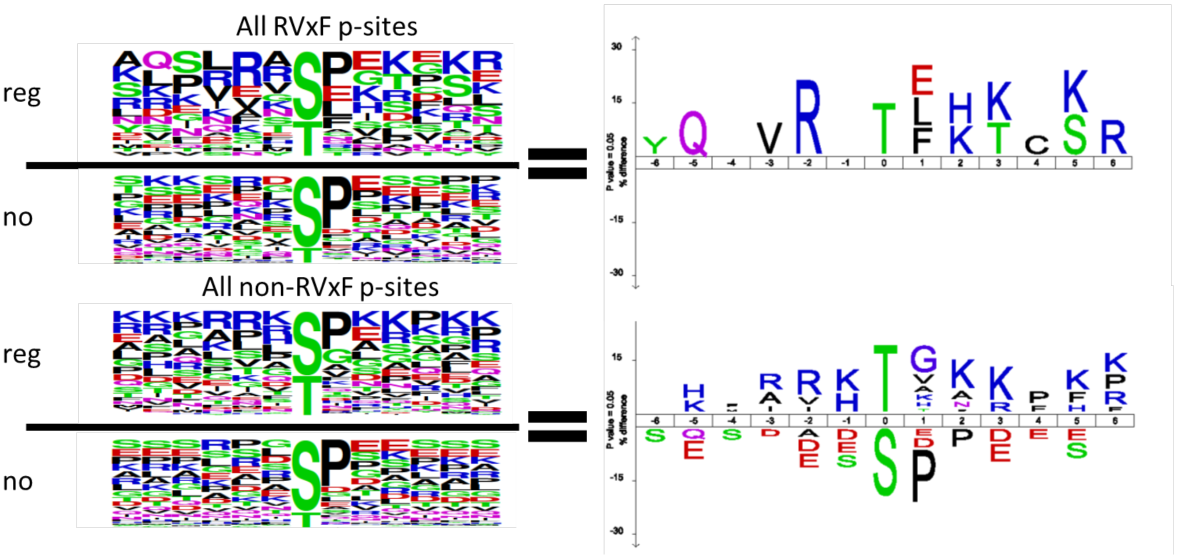

Figure R8.

There is no significant difference in the net charge. However, negatively charged amino acids (D and E) seem to be better tolerated in the +1 and +4 positions of phosphorylation sites on RVxF-containing proteins compared to non-RVxF proteins.

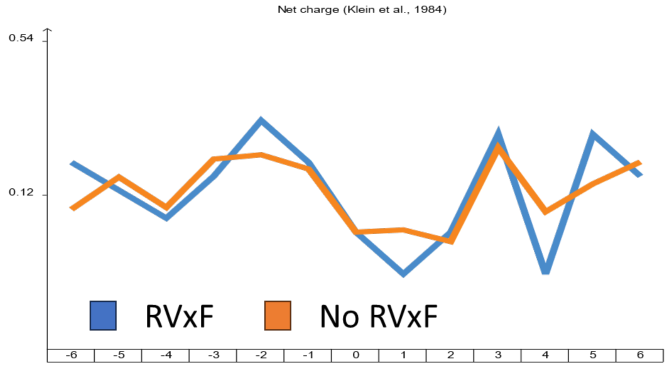

Figure R9.

This analysis supports the conclusion that RVxF motifs can modulate substrate sequence preferences. We have included a line on page 15 in the revised manuscript to summarize this.

10. Were any peptides in the RVxF MRBLE experiment also characterized by ITC or any other classical titration binding experiment? It would be helpful to see how well the Kd values from two orthogonal methods correlate.

In previous work with the B56 regulatory subunit we have found very strong correlation between MRBLE-Pep measurements and ITC data (Hein et al. 2021, PMID 35128539).

In this work, we have not extensively compared the MRBLE-Pep measurements to ITC but there are two peptides from NIPP1 where we have both MRBLE-Pep measurements and ITC data:

PP1γ/Nipp1197-209 AKNSRVTFSEDDEIW: K_d = 0.19 ± 0.01 µM
MRBLE:pep AKNSRVTFSEDDEII: K_d = 3.4 ± 0.7 µM

PP1γ/Nipp1197-209 pT202 AKNSRV(pT)FSEDDEIW: K_d = 4.51 ± 0.20
MRBLE:pep : K_d = 18.5 ± 5 µM

ΔΔG ITC: 1.7 kcal/mol
ΔΔG MRBLE:pep: 0.95 kcal/mol

For both peptides, the K_d value is substantially lower (i.e. the binding is tighter) in ITC measurements than for MRBLE:pep measurements, but the relative differences in binding energies are approximately the same. This difference in absolute affinities is likely due to differences at the C terminus of the peptides. One possibility for the difference in Kd values can be that the C-terminal Ile residues (Ile209 and Ile210 in Nipp1) bind to a second pocket on PP1 (O'Connell et al. Structure 2012). In the ITC experiments the C-terminal IW sequence has the possibility to bind this pocket while in MRBLE-Pep this is the end attached to the beads so might not be able to bind PP1.

11. It is unclear

Suggested minor revisions:
1. Very minor point: In the intro, paragraph 2, it might be useful to clarify whether PP1 and PP2A are the primary PPPs in just eukaryotic cells, or also organismal clades. The current text just says that they are responsible for the majority of Ser/Thr dephosphorylation in cells.

We have clarified this.

2. For the sequences in Figure 1D, it appears that the phospho-Ser and phospho-Thr residues are labeled as Sp

or Tp, whereas these phospho-residues are most conventionally written as pS/pT, which makes reading the sequences a bit confusing.

We have edited all panels of all figures to follow this convention as suggested.

3. It would be helpful if the logos in figure 2E were in the same color scheme as the logos in the rest of Figure

Corrected.

4. Similar to the previous point, the coloring of the sequences in Figure 2D is the opposite of what is in the logos: in the logos (as is often conventional), D/E are red, and K/R are blue, but in the listed sequences the opposite color scheme is used.

We have edited the colors of these sequences to reflect the standard convention.

5. This may be a file processing issue, but Figure S7 is too blurry to read the labels on the nodes in the network diagram.

We have tried to improve quality.

6. In general, it is unclear what the reader stands to learn from the two interaction network diagrams. Related to this, it is quite difficult to discern where PP1 and PP2A-B55 are in these maps. Perhaps the authors could highlight/explain parts of the interaction maps that would be of significance/value to the reader?

We aimed at providing these networks as a resource for the scientific community. We think these networks will be relevant for many researchers working on different biological pathways regulated by these phosphatases. We have now highlighted a few examples of information that can be extracted from these networks. We write on page 12:

Our networks revealed that both PP1 and PP2A-B55 dephosphorylate a diverse set of proteins regulating core biological processes involved in mitotic exit as well as interphase functions. Several known RVxF containing proteins such as KNL1, RIF1 and RepoMan (CDCA2) were substrates of PP1 as expected but our network analysis also suggested that these proteins coordinate dephosphorylation of associated proteins. As an example, KNL1 could scaffold PP1 dephosphorylation of CENPC S261, a phosphorylation site in a nuclear localization sequence. In agreement with previous work PP2A-B55 regulated dephosphorylation of several nucleoporins at mitotic exit consistent with Nup153 and TPR being present in the interactomes. These networks will be useful to dissect PP1 and PP2A-B55 regulation in the future.

7. For non-specialists, it may be helpful to define INCENP and explain its role in mitosis.

We have provided a brief description of INCENP function during mitosis for the non-specialist. We write on page 12:

During anaphase, the chromosomal passenger complex (CPC) translocates from chromatin to the central spindle. The CPC is composed of Aurora B, Borealin, Survivin and INCENP and binding to Mklp2 allows translocation of the complex to the central spindle at anaphase hereby facilitating cytokinesis. INCENP T59, a Cdk1 phosphorylation site, must be dephosphorylated to allow binding to Mklp2 and timely translocation (Fig. 3A) [29].

8. Minor question: is "dephosphorylome" a common term? If not, while this is a purely stylistic consideration, it may by simpler for the readers if some more common phrasing is used.

We have used more common phrasing.

9. In the last sentence of the first paragraph, there is a typo: "phosphoyraltion"

Corrected.

Reviewer #2:

In this study, Hein and co-workers address the complex topic of cellular protein phosphatase selectivity. To do this they use mass spectrometry approaches to analyse dephosphorylation of peptide libraries in vitro by PP1 and PP2A-B55, coupled with global identification of cellular substrates for these enzymes as an in vivo correlate. In summary, there is a considerable amount of high-quality work in the study, and the new data will provide a valuable additional resource for the field.

Specific points:

In Figure 3, INCENP is taken as an example to test a key feature of the dephosphorylation motif. Previous work has shown that INCENP relocalisation from pericentromeric chromatin to the anaphase spindle requires dephosphorylation at T59, and can be prevented with a T59E mutant. This forms a switch controlling binding to a motor protein KIF20A at the onset of anaphase. The charge introduced in the S61E mutant may directly prevent binding to KIF20A. Additional controls are necessary for Figure 3D-E, where a T59A/S61E mutant should be tested. If the authors are correct, then this should to the anaphase spindle since it cannot be phosphorylated. If it fails to localise then defective anaphase localisation is due to a blockade of KIF20A binding and not a dephosphorylation defect.

We agree with the reviewer that this is an important experiment. We have analyzed translocation of INCENP T59V S61E and similar to the INCENP S61E this protein does not translocate at anaphase (see below). Thus, we cannot ascribe the molecular phenotype solely to a defect in T59 dephosphorylation upon introduction of S61E. We suspect that two things are affected by the S61E mutation – firstly T59 dephosphorylation is affected as shown by our in vitro assays and secondly that the binding to KIF20A is likely affected by the mutation or that S61 is also a site of phosphorylation. Based on these new results we have carefully rewritten this section of the paper and pointed this caveat out clearly. However, we still think that the results provides insight into how the surrounding sequences of a phosphorylation site shapes dephosphorylation to potentially regulate biological processes.

It might be possible to identify INCENP mutations that affect T59 dephosphorylation without affecting KIF20A binding but given that we have limited insight into how INCENP and KIF20A bind it is beyond the scope of this manuscript to identify such mutants.

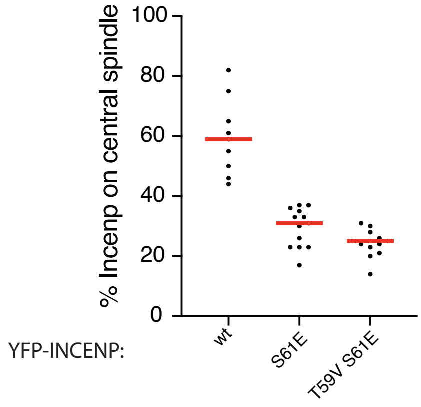

Figure R10. Incenp translocation during anaphase.

We now write on page 13:

To determine if the cellular effects of the S61E mutation was purely attributed to a lack of T59 dephosphorylation we investigated if the T59V mutation could rescue the S61E phenotype. However, the INCENP T59V S61E mutant was unable to translocate to the central spindle. This suggests that the S61E mutation both affect T59 dephosphorylation and possibly Mklp2 binding. We note that S61 might constitute a phosphorylation site although it has not been reported in phosphoproteomic screens.

Our results argue that INCENP T59 dephosphorylation kinetics are affected by the surrounding sequence and that this could contribute to timely INCENP translocation.

The authors note that while the overlap between PP2A-B55 substrates in vivo in their data and previous work is

good, the overlap for PP1 is less clear. There are good reasons for this which need a little more explanation. This also highlights some caveats to the peptide approach, which is important since PP1 is generally thought to act in complex with other regulatory subunits that modify substrate selection. Using PP1 alone in the in vitro approach, and taking Nipp1 as the sole method to modulate PP1 in vivo may not address the full range of PP1 substrates. PP2A-B55 is probably only regulated by Arpp19/ENSA, so the approach would be more general in terms of the range of substrates.

We fully agree with the reviewer on these points. Firstly, we think that previous work using long term RNAi depletion of PP1 are causing too many indirect effects in contrast to our short term inhibition experiments. Secondly previous work adding purified PP1 catalytic subunits to extract might not recapitulate the complexity of PP1 holoenzyme specificity which is why we opted for a strategy to block endogenous PP1 holoenzyme formation through RVxF competition. We think that it is not suprising that these very different approaches would give completely different results. We favor that our strategy in many ways are superior to prior work underscored by the strong number of PP1 regulated sites found in PP1 interactors. However, we phrased our wording more diplomatically in the paper. We have now more carefully explained the differences in the approaches and also pointed out that ARPP19 data is more straightforward to interpret as the active site is inhibited.

We write on page 10:

Our short-term inhibition of PP1 using Nipp1 is quite distinct from a more long-term inhibition using RNAi of PP1 potentially explaining the limited overlap. In contrast asynchronous cell lysate experiments with purified PP1 addition is different from a mitotic lysate where we inhibit RVxF binding and it is not surprising that this will unravel different substrates. As shown later we see a good overlap between PP1 mitotic interactors and PP1 substrates giving us confidence in our ability to identify PP1 regulated sites.

And on page 9:

We used the central domain of Nipp1 and thiophosphorylated Arpp19 (thio-Arpp19) as specific natural inhibitors of PP1 and PP2A-B55, respectively [23-25]. Nipp1 prevents binding of RVxF-containing proteins to PP1, in this way preventing a large subset of PP1 holoenzymes from forming and thus blocking PP1 dephosphorylation. In contrast, thio-Arpp19 blocks the active site of PP2A-B55, effectively blocking activity. It is important to keep in mind that the inhibitors used here act by distinct mechanisms but a present no specific inhibitors of the PP1 active site exists.

Figure 4 is confusing and it is difficult to extract a clear message. Is there some other way to represent the choice of network diagram is not obvious to me, since the study isn't focussed on the interactions between proteins, but rather the sequences of individual phosphatase sites. Is there some way to strengthen the link to Figure 5 in terms of testing whether dephosphorylation alters behaviour of a key PP1 regulated protein or proteins. In that sense the data in Figure S10 is important and could be moved to a main figure.

We thank the reviewer for these comments. The reason for providing this network analysis is that it provides a useful resource for anyone studying PP1 as it integrates both regulated sites and interactions. Given the limited knowledge we have of PP1 regulated pathways this type of network analysis is in our view informative. Secondly, we were interested in understanding the link (if any) between binding of a phosphatase to a protein/substrate and how this might affect the amino acid sequences surrounding dephosphorylated residues. To do this analysis we needed to first obtain this network information as it forms the basis of subsequent analysis. For these reasons we prefer to keep the network but have highlighted in the text a few examples of what type of information can be extracted from it in the main text.

We write on page 14:

Our networks revealed that both PP1 and PP2A-B55 dephosphorylate a diverse set of proteins regulating core biological processes involved in mitotic exit as well as interphase functions. Several known RVxF containing proteins such as KNL1, RIF1 and RepoMan (CDCA2) were substrates of PP1 as expected but our network analysis also suggested that these proteins coordinate dephosphorylation of associated proteins. As an example, KNL1 could scaffold PP1 dephosphorylation of CENPC S261, a phosphorylation site in a nuclear localization

sequence. In agreement with previous work PP2A-B55 regulated dephosphorylation of several nucleoporins at mitotic exit consistent with Nup153 and TPR being present in the interactomes. These networks will be useful to dissect PP1 and PP2A-B55 regulation in the future.

Reviewer #3:

This manuscript describes an adaptation of the MRBLE peptide:protein interaction assay to a format (MRLBE-Dephos) that can be used to measure time-dependent peptide dephosphorylation by phosphatases. The authors have subsequently used this assay to compare the substrate selectivity of two key phosphatases (PP1 and PP2A-B55), either purified or as present in mitotic cell lysates. The MRLBE-Dephos assay is novel and generated interesting data, but needs to be better validated. Also, while the observed determinants of substrate selectivity of PP1 and PP2A-B55 are largely in agreement with previously established determinants, as derived from other experimental approaches, there are obvious differences that need to be further explored. My detailed comments are detailed below.

Specific comments

1. What are the concentrations of the peptides and phosphatases during the MRLBR-dephos assay? The method section only specifies volumes or amounts. This is essential information needed for for comparison with other phosphatase assays.

Each MRBLE:dephos assay reaction contains ~55 beads per code in a 100 µL volume. Given that each bead is estimated to display ~10^7 peptides, this translates into an effective concentration of 55E7 x 1/6.022E23/mol x 1/100E-6L = 83 pM for each peptide, and a total peptide concentration for all peptides of 80 nM.

For the phosphatases, each experiment used 600 ng of PP1, which translates to an effective concentration of 16 µM for a molecular weight of 36965 Da (in vast excess of the available peptide concentration). Each PP2A-B55 experiment used 5 µL of affinity purified PP2A-B55 which results in a final concentration of approximately 300 nM. Please note that the absolute activity between bacterial produced phosphatase and affinity purified phosphatase from cells likely differ.

To clarify, we have added this information to the Materials and Methods.

2. The MRLBE-Dephos assay measures remaining phosphate (high signal→ less high signal), which is less sensitive than classical assays that quantify phosphate release (no signal → signal). This should be commented upon.

While the MRBLE:dephos assay looks for a decrease in signal, the assay itself includes ~50 replicates per timepoint. As sensitivity is a function of assay modality and statistical power, it's not clear that MRBLE:dephos is necessarily less sensitive than classical assays. We further note that in direct comparisons between MRBLE:dephos assays and classical dephosphorylation assays, MRBLE:dephos appears to discriminate between small differences in dephosphorylation that are not resolved via classical assays (Fig. R2), suggesting that MRBLE:dephos may actually have <u>enhanced</u> sensitivity.

3. In all MRLBE dephosphorylation assays (Fig. S1), the dephosphorylation apparently stops before completion. The reason for this is not explained.

We thank the Reviewer for their careful reading and for bringing up this point. In response, we have carefully examined our raw intensity data and imposed an additional filter to prevent analysis of any bead codes for which the initial peptide synthesis was inefficient or unsuccessful, as explained below.

Each experiment contains a negative control peptide that does not include a phosphoresidue (code 96, sequence LGALGARALKRVSA). For both PP1 and PP2A, these beads exhibit a low but non-zero Cy5 intensity (~2500) that does not change with time, as expected. These beads therefore set a lower bound for the 'background' fluorescence intensity we expect from pImago even in the absence of phosphopeptides.

If we examine the raw intensity data over time for each bead code within each experiment, we see 3 main behaviors:

1. *Some codes start out with a very high signal intensity that decreases dramatically. This behavior is consistent with a bead that had large amounts of phosphorylated residues at the start of the experiment (time t=0, prior to the addition of phosphatase) that were then removed by the enzyme. Most of these curves for PP1 end up decreasing down to a Cy5 intensity that is within 2-fold of the expected background signal, suggesting that these dephosphorylation reactions have gone to completion.*
2. *Some codes start out with a very high signal intensity that stays pretty high throughout the course of the experiment. This behavior is consistent with a bead that had large amounts of phosphorylated residues at the start of the experiment and these phosphorylated residues were unaffected by the addition of the enzyme. In this case, no dephosphorylation takes place because the peptide sequence is not a substrate for the phosphatase and the failure to go to completion is not a concern.*
3. *A small number of codes start out with a fairly low signal intensity that is similar to background. This behavior is consistent with a bead for which the initial synthesis of phosphorylated peptide was inefficient or unsuccessful, and these data should not be included in downstream analyses.*
4. *A small number of codes (2-3 codes per experiment) start with a fairly high signal intensity that decreases but does not decrease all the way to background levels, consistent with the Reviewer's concern.*

To identify and filter out bead codes with unsuccessful phosphopeptide synthesis, we now impose a requirement that the median intensity for all beads from a given code at the start of the experiment must be greater than a threshold value (calculated as the median + 1 standard deviation of the negative control beads). This requirement eliminates a small number of codes throughout the experiments but ensures that the remaining values represent high quality data. We have edited the Materials and Methods to clarify this.

For the codes that decrease but not all the way to background levels, this behavior is consistent with a population of phosphorylated peptide that is not dephosphorylated by the added phosphatase.

To allow readers to easily view and consider data at every step of the analysis process, we now include a figure showing the raw data from a single experiment (Fig. S2) and provide the following files as Supplementary Data:
• Per-experiment files showing raw intensities for each code over time.
• Per-experiment files showing normalized intensities for each code over time.
• Per-code files showing raw intensities for all 3 experiments over time.
• Per-code files showing normalized intensities for all 3 experiments over time.
• Jupyter notebooks with all analysis code.

We'd like to again thank the Reviewer for their question – this type of question is a clear instance in which peer review allowed us to reconsider the data and change the data analysis to be significantly more robust.

Also, since the dephosphorylation is not linear with time (Fig. S1), it is not clear how the dephosphorylation score can be accurately derived from the first and last time points.

Beads either are dephosphorylated largely to completion or are not dephosphorylated at all. Thus, the final intensity divided by the starting intensity provides a reasonable metric for the degree of dephosphorylation that occurred over the course of the experiment.

4. A comparison of classical phosphatase assays (phosphate release) and the MRLBE-Dephos score (Fig. 1E) shows essentially no dephosphorylation for some peptides with the first assay but still very significant dephosphorylation with the second assay. This indicates that the MRLBE-Dephos assay not only measures phosphatase activity but, e.g., also loss of bead-associated peptide.

We again appreciate the Reviewer's careful reading and consideration of the data. From looking at the raw data, it's clear that many of the codes do not show any loss of signal over the course of the experiment. This rules out any significant loss of bead-associated peptide over the course of the experiment.

However, the Reviewer is correct that the original figure showed more significant dephosphorylation of some peptides with the second assay. Going back to the original intensities, it is clear that 1 of the 5 peptides in the MRBLE:dephos assays had low signal intensities at the start of the experiment that did not differ significantly from the negative control intensities, suggesting poor synthesis of phosphorylated peptides on those samples. As described above, we now filter out sequences for which phosphopeptide synthesis was not successful, which eliminates 1 sequence from consideration. We present a revised version of Figure 1F that shows strong correlation between the 2 assays.

5. Since the peptides were synthesized on the beads, it should be explored whether binding to the beads affects their properties as substrates.

While this is a valid concern, it is (unfortunately) beyond the scope of what is possible at this time. As we are able to measure many peptides in parallel in a single experiment, the most relevant information is the information about how specific amino acid changes change the relative binding affinity. We have indicated these points in the revised manuscript.

We are by no means claiming that MRBLE-Dephos fully represents the complex biological dephosphorylation reactions occurring in cells with complex substrates and holoenzymes, but it provides an approach for high-throughput characterization of dephosphorylation reactions not possible by any other current method.

6. It is biologically not relevant to compare the substrate selectivity of the PP1 monomer, which does not exist in vivo, with a PP2A-B55 holoenzyme. Also, bacterially expressed PP1 has a substrate selectivity that is very different from that of native PP1 (https://doi.org/10.1111/j.1432-1033.1993), making a direct comparison even more irrelevant.

We do not disagree with the reviewer on these concerns and are not claiming that the results from MRBLE-Pep explains the complexity of in vivo dephosphorylation which is clearly controlled by multiple protein-protein interactions (as also highlighted in the second part of the paper). However, as part of developing new methods for the field we think that using recombinant PP1 and purified PP2A-B55 holoenzyme is an appropriate test/comparison.

We now write on page 8-9:

In conclusion, we develop a generally applicable high throughput dephosphorylation assay that allows quantitative mapping of protein phosphatase dephosphorylation motifs using only very small amounts of material. We use this to show the existence of optimal dephosphorylation site signatures for PP1 and PP2A-B55, providing a set of rules for accessing whether a phosphorylation site is optimal for one of these phosphatases. In vivo these rules will be further shaped by the PP1 holoenzyme composition as well as substrate interactions.

7. The most striking feature of the comparison between purified PP1 and PP2A-B55 is their similar activity on a broad range of peptide substrates (Fig. 1D; Fig. 2A-D). It is difficult to grasp how the observed minor differences can explain the clearly distinct, albeit somewhat overlapping functions, of these phosphatases in vivo. The differences in substrate specificity would most likely have been much more obvious if a specific PP1 holoenzyme and PP2A holoenzyme were compared, with the free catalytic subunits as reference points (see previous comment).

See our comment above. Indeed, specific holoenzymes of PP1 and binding of PP2A-B55 to specific proteins are major determinants of specificity and we have in our view not ignored this but rather in the second half of the manuscript explored this aspect extensively. However, our data do suggest that there are also inherent preferences in the active site of phosphatases (consistent with previous work) that can contribute.

8. It is not correct to refer to 'benchmark in vitro data with in vivo substrates' (p8, line 1) when the actual comparison is purified phosphatases versus phosphatases present in mitotic lysates.

We have rephrased the sentence.

9. While thio-phosphorylated proteins are somewhat resistant to dephosphorylation, they will eventually also be dephosphorylated. Hence, the authors should examine to which extent thiophosphorylated Arpp19 is dephosphorylated in the adopted timeframe(s). Nipp1 is indeed a highly selective inhibitor of PP1 but it only prevents the dephosphorylation of a subset of substrates. This limitation should be indicated.

We are always comparing thio-Arpp19 to Arpp19 S62A ensuring that the regulated sites observed are due to PP2A-B55 inhibition. We have not been able to detect thio-Arpp19 by western blot with our phosphoantibody so we cannot tell whether there is dephosphorylation of thio-Arpp19 occuring during the 5-minute incubation. However, even if thio-Arpp19 was getting dephosphorylated and PP2A-B55 not fully inhibited we are comparing to a sample treated with Arpp19 S62A.
We agree that Nipp1 only blocks a subset and have indicated this in the revised manuscript.

We now write on page 9:

We used the central domain of Nipp1 and thiophosphorylated Arpp19 (thio-Arpp19) as specific natural inhibitors of PP1 and PP2A-B55, respectively [23-25]. Nipp1 prevents binding of RVxF-containing proteins to PP1, in this way preventing a large subset of PP1 holoenzymes from forming and thus blocking PP1 dephosphorylation. In contrast, thio-Arpp19 blocks the active site of PP2A-B55, effectively blocking activity. It is important to keep in mind that the inhibitors used here act by distinct mechanisms but a present no specific inhibitors of the PP1 active site exists. As controls, we used Nipp1 with a mutated RVxF motif preventing PP1 binding and Arpp19 S62A that cannot inhibit PP2A-B55. An antibody detecting all proline-directed threonine phosphorylation (TpP) as well as H3S10 confirmed the specific inhibition of phosphatases upon addition of inhibitors (Fig. S5). We do not know the extent of inhibition but the relative comparison to appropriate controls allows us to identify regulated sites.

10. Less than 10% of the identified mitotic phosphorylation sites (> 38,000) were affected by PP1 or PP2A-B55 inhibition. This indicates that the inhibition of these phosphatases was incomplete and/or that it is incorrect to that these phosphatases represent the major mitotic-exit phosphatases.

Both options are a possibility. Since we are only inhibiting for 5 minutes it can also be that certain substrates need longer time to get dephosphorylated. Finally, we cannot exclude residual kinase activity. What is important is the clear difference in regulated sites we observe – we cannot make and are not making any claims on the fact that less than 10% of sites are regulated.

11. p9, first line: H3S29 does not exist. Did the authors aim to refer to H3S28?

The reviewer is correct. We have changed this in the revised manuscript.

12. The limited overlap between PP1 substrates in mitotic lysates and previous substrate-mapping studies is worrysome, and is further evidence that PP1 was only partially blocked in the mitotic lysates.

See also our comment to point 2 by reviewer 2. There is no way that we can make any claims on full versus partial inhibition of PP1 based on limited overlap to previous studies given how different they are in experimental setup. We favor that our strategy is a reasonable strategy given the short time of inhibition of endogenous PP1 holoenzymes which is supported by the strong overlap with PP1 interactors (Fig. 4A).

We now write on page 10:

Our short-term inhibition of PP1 using Nipp1 is quite distinct from a more long-term inhibition using RNAi of PP1 potentially explaining the limited overlap. In contrast asynchronous cell lysate experiments with purified PP1 addition is different from a mitotic lysate where we inhibit RVxF binding, and it is not surprising that this will unravel different substrates. As shown later we see a good overlap between PP1 mitotic interactors and PP1 substrates giving us confidence in our ability to identify PP1 regulated sites.

13. The identified PP1 and B55 interactors (Table S2) are poorly described and not independently validated. Are they regulatory subunits and/or substrates? How much overlap is there with known interactors?

Table S2 contains proteins from mass spectrometry analysis of affinity purified PP1 and B55 subunits or from proximity-dependent ligation assays with PP1 and B55. This type of data does not allow us to discriminate between regulatory subunits or substrates while integration with the phosphoproteomic data can provide this insight.

We now write on page 14:

We defined interactomes of PP1g and B55a during mitosis using affinity purification of YFP-tagged subunits and also proximity-dependent ligation using miniTurbo to capture interactions at mitotic exit (Fig. S6) [30, 31]. This resulted in the identification of 923 PP1 interactors and 141 B55 interactors (Dataset EV2). These identified proteins can constitute both regulators and substrates.

14. The interaction of PP1 with RVxF motifs and its regulation by mitotic phosphorylation of residues within or next to the RVxF motif has already been studied in some detail by various research groups and is not properly referred to in the manuscript.

We have provided additional references for the known phosphorylation dependent regulation of PP1 binding to RVxF motifs.

9th Oct 2023

Manuscript Number: MSB-2023-11782R
Title: Phosphatase specificity principles uncovered by MRBLE-Dephos and global substrate identification

Dear Jakob,

Thank you for sending us your revised manuscript. We have now heard back from the two reviewers who were asked to evaluate your revised study. As you will see below, the reviewers think that the study has improved after the performed revisions. They raise however a series of remaining concerns, which we would ask you to address in a final round of revision. We would also ask you to address some remaining editorial issues listed below.

- Our data editors have noticed some unclear or missing information in the figure legends, please see the attached .doc file. We have also made some minor formatting edits. Please make all requested text changes using the attached file and *keeping the "track changes" mode* so that we can easily access the edits made.

- There are callouts for Table S1-S3, but no such tables are uploaded, please fix these callouts.

- The number of EV Figures is large (typically we allow only 5). We would ask you to provide all these figures in in a PDF called Appendix. Appendix figures should be labeled and called out as: "Appendix Figure S1, Appendix Figure S2... Appendix Table S1..." etc. Each legend should be below the corresponding Figure/Table in the Appendix. Please include a Table of Contents in the beginning of the Appendix. For detailed instructions regarding expanded view please refer to our Author Guidelines: .

- Source data files need to be reorganized to one file (or zip folder per figure). For Appendix figures, all source data should be provided in a single zip folder.

Please resubmit your revised manuscript online, with a covering letter listing amendments and responses to each point raised by the referees. Please resubmit the paper **within one month** and ideally as soon as possible. If we do not receive the revised manuscript within this time period, the file might be closed and any subsequent resubmission would be treated as a new manuscript. Please use the Manuscript Number (above) in all correspondence.

Click on the link below to submit your revised paper.

https://msb.msubmit.net/cgi-bin/main.plex

Thank you for submitting this paper to Molecular Systems Biology.

Kind regards,

Maria

Maria Polychronidou, PhD
Senior Editor
Molecular Systems Biology

If you do choose to resubmit, please click on the link below to submit the revision online before 8th Nov 2023.

https://msb.msubmit.net/cgi-bin/main.plex

IMPORTANT:
Please note that corresponding authors are required to supply an ORCID ID for their name upon submission of a revised manuscript (EMBO Press signed a joint statement to encourage ORCID adoption).
(https://www.embopress.org/page/journal/17444292/authorguide#editorialprocess)
Currently, our records indicate that the ORCID for your account is 0000-0003-4100-1125.

Please click the link below to modify this ORCID:
Link Not Available

The system will prompt you to fill in your funding and payment information. This will allow Wiley to send you a quote for the

article processing charge (APC) in case of acceptance. This quote takes into account any reduction or fee waivers that you may be eligible for. Authors do not need to pay any fees before their manuscript is accepted and transferred to the publisher.

As a matter of course, please make sure that you have correctly followed the instructions for authors as given on the submission website.

*** PLEASE NOTE *** As part of the EMBO Press transparent editorial process initiative (see our Editorial at https://dx.doi.org/10.1038/msb.2010.72 , Molecular Systems Biology will publish online a Review Process File to accompany accepted manuscripts. When preparing your letter of response, please be aware that in the event of acceptance, your cover letter/point-by-point document will be included as part of this File, which will be available to the scientific community. More information about this initiative is available in our Instructions to Authors. If you have any questions about this initiative, please contact the editorial office (msb@embo.org).

Reviewer #1:

Overall, this revised manuscript is substantially improved, and the revisions/responses are extremely thorough. This work will be an important addition to the phosphatase field, both in terms of new methods for in vitro substrate specificity characterization, as well as signaling network information for PP1 and PP2A.

A few minor corrects are suggested below:

1. For Figure 2C, it seems that not all of the C-terminal sequences in the diagram have residue coloring, as the N-terminal ones do. Also, it was a bit confusing to have the +1 P listed as part of the N-terminal sequence, given that it is C-terminal to the pT residue.

2. It's unclear what sets of peptides in 2C this sentence is referring to, "For PP1 N-terminal basic residues could compensate for the negative impact of an acidic or glycine residue in pos +2 (Fig. 2C) ." Is there potentially a typo here?

3. Pg. 9: "distinct mechanisms but a present no specific inhibitors of the PP1 active site exists" should be, "but at present..."

4. Pg. 10: "proline-directed threonine phosphorylation (TpP)" should be changed to "pTP" for consistency with the changes in the initial revision.

5. Figure 4D, label on the right says "none regulated sites" but should say "non-regulated sites"

6. The network diagram in 4C is no longer blurry, but the one in EV9 is still blurry.

Reviewer #2:

The authors have carried out a thorough revision of the manuscript and responded to most of the questions raised in a clear fashion. in my initial review, I raised a question about the cell biology experiments studying INCENP dephosphorylation, specifically did the mutations preventing B55 recognition also have an effect on the interaction with the kinesin motor needed to carry INCENP in anaphase. This has been addressed in part, but the new data seem to indicate that the B55 mutation does in fact perturb the interaction with the kinesin KIF20A. This means localisation of these INCENP mutants to the anaphase spindle is not possible, irrespective of phosphorylation state. I recommend this imaging data and the accompanying graphs are removed. The biochemical and mass spectrometry analysis is clear and tests the role of B55, however the caveat is the mutant isn't suitable for in vivo functional studies.

Reviewer #3:

Reviewer 3

In their reply to comment 1, the authors reported that in their assays the phosphatases concentrations (300 nM for PP2A-B56 and 16,000 nM for PP1) were several orders of magnitude higher than the (peptide) substrates (80 nM in total). This is very worrysome, as enzyme assays need to be performed with substrates in a large molar excess. Even with a turnover number (reactions catalyzed/sec/molecule enzyme) for these phosphatases of only 1 -in reality the turnover number is way higher-, all substrate would be hydrolyzed within 0.5 (PP1) to 20 (PP2A) sec, unless the used phosphatase preparations are largely inactive. Yet, the authors adopted an assay time of 14,400 sec (5h). Moreover, enzyme activities can only be properly derived from the rate of substrate-product conversion up to 30% of substrate depletion because the curve flattens at later time points. It

therefore remains incomprehensible for this reviewer (see comment 3) how phosphatase activities can be derived from the first and last time points of 5h-assays, when all substrate appears to be hydrolyzed at the early time points. In conclusion, the adopted assay does not appear to meet the minimal standards for enzyme assays and does not yield conclusive data.

The authors agree with several other criticisms (points 5, 6, 7, 10, 12), but do not seem to grasp the implications for the interpretation and value of their data.

Reviewer #1:

Overall, this revised manuscript is substantially improved, and the revisions/responses are extremely thorough. This work will be an important addition to the phosphatase field, both in terms of new methods for in vitro substrate specificity characterization, as well as signaling network information for PP1 and PP2A.

A few minor corrects are suggested below:

1. For Figure 2C, it seems that not all of the C-terminal sequences in the diagram have residue coloring, as the N-terminal ones do. Also, it was a bit confusing to have the +1 P listed as part of the N-terminal sequence, given that it is C-terminal to the pT residue.

Our response:
We have corrected this.

2. It's unclear what sets of peptides in 2C this sentence is referring to, "For PP1 N-terminal basic residues could compensate for the negative impact of an acidic or glycine residue in pos +2 (Fig. 2C) ." Is there potentially a typo here?

Our response:
We have corrected the text to clarify that Fig. 2C only shows this for acidic residues.

3. Pg. 9: "distinct mechanisms but a present no specific inhibitors of the PP1 active site exists" should be, "but at present..."

Our response:
Corrected

4. Pg. 10: "proline-directed threonine phosphorylation (TpP)" should be changed to "pTP" for consistency with the changes in the initial revision.

Our response:
Corrected

5. Figure 4D, label on the right says "none regulated sites" but should say "non-regulated sites"

Our response:

Corrected

6. The network diagram in 4C is no longer blurry, but the one in EV9 is still blurry.

Our response:
Corrected in the new appendix file.

Reviewer #2:

The authors have carried out a thorough revision of the manuscript and responded to most of the questions raised in a clear fashion. in my initial review, I raised a question about the cell biology experiments studying INCENP dephosphorylation, specifically did the mutations preventing B55 recognition also have an effect on the interaction with the kinesin motor needed to carry INCENP in anaphase. This has been addressed in part, but the new data seem to indicate that the B55 mutation does in fact perturb the interaction with the kinesin KIF20A. This means localisation of these INCENP mutants to the anaphase spindle is not possible, irrespective of phosphorylation state. I recommend this imaging data and the accompanying graphs are removed. The biochemical and mass spectrometry analysis is clear and tests the role of B55, however the caveat is the mutant isn't suitable for in vivo functional studies.

Our response:
We clearly indicated this in the text of the revised manuscript and prefer to keep data as the effect of S61E is two-fold: it affects T59 dephosphorylation but also KIF20A binding. As we clearly state this caveat, we think the data are still relevant to keep.

Reviewer #3:

In their reply to comment 1, the authors reported that in their assays the phosphatases concentrations (300 nM for PP2A-B56 and 16,000 nM for PP1) were several orders of magnitude higher than the (peptide) substrates (80 nM in total). This is very worrysome, as enzyme assays need to be performed with substrates in a large molar excess. Even with a turnover number (reactions catalyzed/sec/molecule enzyme) for these phosphatases of only 1 -in reality the turnover number is way higher-, all substrate would be hydrolyzed within 0.5 (PP1) to 20 (PP2A) sec, unless the used phosphatase preparations are largely inactive. Yet, the authors adopted an assay time of 14,400 sec (5h). Moreover, enzyme activities can only be properly derived from the rate of substrate-product conversion up to 30% of substrate depletion because the curve flattens at later time points. It therefore remains incomprehensible for this reviewer (see

comment 3) how phosphatase activities can be derived from the first and last time points of 5h-assays, when all substrate appears to be hydrolyzed at the early time points. In conclusion, the adopted assay does not appear to meet the minimal standards for enzyme assays and does not yield conclusive data.

Our response:

We agree completely with the Reviewer that careful control and quantification of enzyme and substrate concentrations are essential for reporting accurate kinetic parameters and inhibition constants (e.g. k_{cat}, K_M, k_{cat}/K_M, and K_i). This is typically done by varying substrate concentrations, measuring initial rates during the linear regime of substrate turnover, plotting these rates as a function of substrate concentration, and fitting Michaelis-Menten curves to the data to extract best fit values for these parameters. In the Reviewer's discussion of minimal guidelines and standards for enzyme assays, they appear to be suggesting conditions used to derive these kinetic and thermodynamic constants. We have done these types of analyses extensively in prior work for alkaline phosphatases (see, for example, Markin, Mokhtari* et al., Science 2021; Markin et al., PNAS 2023).*

However, these concerns are not relevant here. We are not attempting to quantify kinetic or thermodynamic constants in MRBLE-dephos assays – and we do not claim to do so. The MRBLE-dephos assay incubates a phosphatase of interest with a selection of bead-bound peptides in a single volume; imaging these beads reveals sequence-specific differences in phosphorylation efficiency over time. We report these relative sequence-specific differences in phosphorylation activity as a "dephosphorylation score" that compares the percentage of remaining phosphates on peptides of each sequence by the end of the assay. This score has no physical meaning (it is not a thermodynamic or kinetic constant); instead, the "dephosphorylation score" simply provides a convenient scalar number that reports on the fact that while some sequences are dephosphorylated efficiently by a given phosphatase, others are unaffected. Peptides with a high "dephosphorylation score" are preferentially desphosphorylated in this in vitro assay relative to other peptides and are therefore also likely to be preferentially dephosphorylated in cells; thus, the "dephosphorylation score" carries useful information about relative sequence preferences.

The authors agree with several other criticisms (points 5, 6, 7, 10, 12), but do not seem to grasp the implications for the interpretation and value of their data.

Our response:

The reviewer pointed to certain limitations of MRBLE-Pep (point 5-7) and the extension of its results to in vivo regulation of phosphatases. Although we have acknowledged these limitations in the revised manuscript, we still find that MRBLE-Dephos provides an important technical advancement and that its results provide important insight into inherent phosphatase specificity. We have been very clear in the manuscript on the role of binding partners in controlling phosphatase specificity. Furthermore, the concerns raised by the reviewer regarding the mitotic lysate data (points 10,12) and approach we do not share. The fact that only 10% of sites are found to be regulated by our approach and for PP1 we see a limited overlap with previous screens was clearly addressed in our previous rebuttal.

16th Oct 2023

Manuscript number: MSB-2023-11782RR
Title: Phosphatase specificity principles uncovered by MRBLE-Dephos and global substrate identification

Dear Jakob,

Thank you again for sending us your revised manuscript. We are now satisfied with the modifications made and I am pleased to inform you that your paper has been accepted for publication.

*** PLEASE NOTE *** As part of the EMBO Publications transparent editorial process initiative (see our Editorial at https://dx.doi.org/10.103/msb.2010.72), Molecular Systems Biology publishes online a Review Process File with each accepted manuscripts. This file will be published in conjunction with your paper and will include the anonymous referee reports, your point- by-point response and all pertinent correspondence relating to the manuscript. If you do NOT want this File to be published, please inform the editorial office at msb@embo.org within 14 days upon receipt of the present letter.

LICENSE AND PAYMENT:
All articles published in Molecular Systems Biology are fully open access: immediately and freely available to read, download and share.

Molecular Systems Biology charges an article processing charge (APC) to cover the publication costs. You, as the corresponding author for this manuscript, should have already received a quote with the article processing fee separately. Please let us know in case this quote has not been received.

Once your article is at Wiley for editorial production, you will receive an email from Wiley's Author Services system, which will ask you to log in and will present you with the publication license form for completion. Within the same system the publication fee can be paid by credit card, an invoice or pro forma can be requested.

Payment of the publication charge and the signed Open Access Agreement form must be received before the article can be published online.

Upon acceptance it is mandatory for you to return the completed payment form. Failure to send back the form may result in a delay in the publication of your paper.

Molecular Systems Biology articles are published under the Creative Commons licence CC BY, which facilitates the sharing of scientific information by reducing legal barriers, while mandating attribution of the source in accordance to standard scholarly practice.

Proofs will be forwarded to you within the next 2-3 weeks.

Thank you very much for submitting your work to Molecular Systems Biology.

Kind regards,

Maria

Maria Polychronidou, PhD
Senior Editor
Molecular Systems Biology

EMBO Press Author Checklist

Corresponding Author Name: Jakob Nilsson
Journal Submitted to: Molecular Systems Biology
Manuscript Number: MSB-2023-11782

USEFUL LINKS FOR COMPLETING THIS FORM
The EMBO Journal - Author Guidelines
EMBO Reports - Author Guidelines
Molecular Systems Biology - Author Guidelines
EMBO Molecular Medicine - Author Guidelines

Reporting Checklist for Life Science Articles (updated January
This checklist is adapted from Materials Design Analysis Reporting (MDAR) Checklist for Authors. MDAR establishes a minimum set of requirements in transparent reporting in the life sciences (see Statement of Task: 10.31222/osf.io/9sm4x). Please follow the journal's guidelines in preparing your
Please note that a copy of this checklist will be published alongside your article.

Abridged guidelines for figures
1. Data
The data shown in figures should satisfy the following conditions:
- the data were obtained and processed according to the field's best practice and are presented to reflect the results of the experiments in an accurate and unbiased manner.
- ideally, figure panels should include only measurements that are directly comparable to each other and obtained with the same assay.
- plots include clearly labeled error bars for independent experiments and sample sizes. Unless justified, error bars should not be shown for technical
- if n<5, the individual data points from each experiment should be plotted. Any statistical test employed should be justified.
- Source Data should be included to report the data underlying figures according to the guidelines set out in the authorship guidelines on Data

2. Captions
Each figure caption should contain the following information, for each panel where they are relevant:
- a specification of the experimental system investigated (eg cell line, species name).
- the assay(s) and method(s) used to carry out the reported observations and measurements.
- an explicit mention of the biological and chemical entity(ies) that are being measured.
- an explicit mention of the biological and chemical entity(ies) that are altered/varied/perturbed in a controlled manner.
- the exact sample size (n) for each experimental group/condition, given as a number, not a range;
- a description of the sample collection allowing the reader to understand whether the samples represent technical or biological replicates (including how many animals, litters, cultures, etc.).
- a statement of how many times the experiment shown was independently replicated in the laboratory.
- definitions of statistical methods and measures:
  - common tests, such as t-test (please specify whether paired vs. unpaired), simple χ^2 tests, Wilcoxon and Mann-Whitney tests, can be unambiguously identified by name only, but more complex techniques should be described in the methods section;
  - are tests one-sided or two-sided?
  - are there adjustments for multiple comparisons?
  - exact statistical test results, e.g., P values = x but not P values < x;
  - definition of 'center values' as median or average;
  - definition of error bars as s.d. or s.e.m.

Please complete ALL of the questions below.
Select "Not Applicable" only when the requested information is not relevant for your study.

Materials

Newly Created Materials	Information included in the manuscript?	In which section is the information available? (Reagents and Tools Table, Materials and Methods, Figures, Data Availability Section)
New materials and reagents need to be available; do any restrictions apply?	Not Applicable	

Antibodies	Information included in the manuscript?	In which section is the information available? (Reagents and Tools Table, Materials and Methods, Figures, Data Availability Section)
For **antibodies** provide the following information:<br>- Commercial antibodies: RRID (if possible) or supplier name, catalogue number and or/clone number<br>- Non-commercial: RRID or citation	Yes	Materials and methods

DNA and RNA sequences	Information included in the manuscript?	In which section is the information available? (Reagents and Tools Table, Materials and Methods, Figures, Data Availability Section)
**Short novel DNA or RNA including primers, probes**: provide the sequences.	Yes	Materials and methods

Cell materials	Information included in the manuscript?	In which section is the information available? (Reagents and Tools Table, Materials and Methods, Figures, Data Availability Section)
**Cell lines:** Provide species information, strain. Provide accession number in repository **OR** supplier name, catalog number, clone number, and/**OR** RRID.	Yes	Materials and methods
**Primary cultures:** Provide species, strain, sex of origin, genetic modification status.	Not Applicable	
Report if the cell lines were recently **authenticated** (e.g., by STR profiling) and tested for mycoplasma contamination.	Yes	Materials and methods

Experimental animals	Information included in the manuscript?	In which section is the information available? (Reagents and Tools Table, Materials and Methods, Figures, Data Availability Section)
**Laboratory animals or Model organisms:** Provide species, strain, sex, age, genetic modification status. Provide accession number in repository **OR** supplier name, catalog number, clone number, **OR** RRID.	Not Applicable	
**Animal observed in or captured from the field:** Provide species, sex, and age where possible**.**	Not Applicable	
Please detail **housing and husbandry conditions**.	Not Applicable	

Plants and microbes	Information included in the manuscript?	In which section is the information available? (Reagents and Tools Table, Materials and Methods, Figures, Data Availability Section)
**Plants:** provide species and strain, ecotype and cultivar where relevant, unique accession number if available, and source (including location for collected wild specimens).	Not Applicable	
**Microbes:** provide species and strain, unique accession number if available, and source.	Not Applicable	

Human research participants	Information included in the manuscript?	In which section is the information available? (Reagents and Tools Table, Materials and Methods, Figures, Data Availability Section)
If collected and within the bounds of privacy constraints report on age, sex and gender or ethnicity for all study participants.	Not Applicable	

Core facilities	Information included in the manuscript?	In which section is the information available? (Reagents and Tools Table, Materials and Methods, Figures, Data Availability Section)
If your work benefited from core facilities, was their service mentioned in the acknowledgments section?	Not Applicable	

Design

Study protocol	Information included in the manuscript?	In which section is the information available? (Reagents and Tools Table, Materials and Methods, Figures, Data Availability Section)
If study protocol has been **pre-registered, provide DOI in the manuscript**. For clinical trials, provide the trial registration number **OR** cite DOI.	Not Applicable	
Report the **clinical trial registration number** (at ClinicalTrials.gov or equivalent), where applicable.	Not Applicable	

Laboratory protocol	Information included in the manuscript?	In which section is the information available? (Reagents and Tools Table, Materials and Methods, Figures, Data Availability Section)
Provide DOI OR other citation details if **external detailed step-by-step protocols** are available.	Not Applicable	

Experimental study design and statistics	Information included in the manuscript?	In which section is the information available? (Reagents and Tools Table, Materials and Methods, Figures, Data Availability Section)
Include a statement about **sample size** estimate even if no statistical methods were used.	Yes	Materials and methods
Were any steps taken to minimize the effects of subjective bias when allocating animals/samples to treatment (e.g. **randomization procedure**)? If yes, have they been described?	Not Applicable	
Include a statement about **blinding** even if no blinding was done.	Yes	Materials and methods
Describe **inclusion/exclusion criteria** if samples or animals were excluded from the analysis. Were the criteria pre-established?<br><br>If sample or data points were omitted from analysis, report if this was due to attrition or intentional exclusion and provide justification.	Yes	Materials and methods
For every figure, are **statistical tests** justified as appropriate? Do the data meet the assumptions of the tests (e.g., normal distribution)? Describe any methods used to assess it. Is there an estimate of variation within each group of data? Is the variance similar between the groups that are being statistically compared?	Yes	Figure legends

Sample definition and in-laboratory replication	Information included in the manuscript?	In which section is the information available? (Reagents and Tools Table, Materials and Methods, Figures, Data Availability Section)
In the figure legends: state number of times the experiment was **replicated** in laboratory.	Yes	Figure legend
In the figure legends: define whether data describe **technical or biological replicates**.	Yes	Figure legend

Ethics

Ethics	Information included in the manuscript?	In which section is the information available? (Reagents and Tools Table, Materials and Methods, Figures, Data Availability Section)
Studies involving **human participants**: State details of **authority granting ethics approval** (IRB or equivalent committee(s), provide reference number for approval.	Not Applicable	
Studies involving **human participants**: Include a statement confirming that **informed consent** was obtained from all subjects and that the experiments conformed to the principles set out in the WMA Declaration of Helsinki and the Department of Health and Human Services Belmont Report.	Not Applicable	
Studies involving **human participants:** For publication of **patient photos**, include a statement confirming that consent to publish was obtained.	Not Applicable	
Studies involving experimental **animals**: State details of **authority granting ethics approval** (IRB or equivalent committee(s), provide reference number for approval. Include a statement of compliance with ethical regulations.	Not Applicable	
Studies involving **specimen and field samples:** State if relevant **permits** obtained, provide details of authority approving study; if none were required, explain why.	Not Applicable	

Dual Use Research of Concern (DURC)	Information included in the manuscript?	In which section is the information available? (Reagents and Tools Table, Materials and Methods, Figures, Data Availability Section)
Could your study fall under dual use research restrictions? Please check biosecurity documents and list of **select agents and toxins** (CDC): https://www.selectagents.gov/sat/list.htm	Not Applicable	
If you used a select agent, is the security level of the lab appropriate and reported in the manuscript?	Not Applicable	
If a study is subject to dual use research of concern regulations, is the name of the **authority granting approval and reference number** for the regulatory approval provided in the manuscript?	Not Applicable	

Reporting

The MDAR framework recommends adoption of discipline-specific guidelines, established and endorsed through community initiatives. Journals have their own policy about requiring specific guidelines and recommendations to complement MDAR.

Adherence to community standards	Information included in the manuscript?	In which section is the information available? (Reagents and Tools Table, Materials and Methods, Figures, Data Availability Section)
State if relevant guidelines or checklists (e.g., **ICMJE, MIBBI, ARRIVE, PRISMA**) have been followed or provided.	Not Applicable	
For **tumor marker prognostic studies,** we recommend that you follow the **REMARK** reporting guidelines (see link list at top right). See author guidelines, under 'Reporting Guidelines'. Please confirm you have followed these guidelines.	Not Applicable	
For **phase II and III randomized controlled trials**, please refer to the **CONSORT** flow diagram (see link list at top right) and submit the CONSORT checklist (see link list at top right) with your submission. See author guidelines, under 'Reporting Guidelines'. Please confirm you have submitted this list.	Not Applicable	

Data Availability

Data availability	Information included in the manuscript?	In which section is the information available? (Reagents and Tools Table, Materials and Methods, Figures, Data Availability Section)
Have **primary datasets** been deposited according to the journal's guidelines (see 'Data Deposition' section) and the respective accession numbers provided in the Data Availability Section?	Yes	Data availability section
Were **human clinical and genomic datasets** deposited in a public access-controlled repository in accordance to ethical obligations to the patients and to the applicable consent agreement?	Not Applicable	
Are **computational models** that are central and integral to a study available without restrictions in a machine-readable form? Were the relevant accession numbers or links provided?	Yes	Data availability section
If publicly available data were reused, provide the respective **data citations in the reference list**.	Yes	Reference list
